# Repressive chromatin modification underpins the long-term expression trend of a perennial flowering gene in nature

**DOI:** 10.1038/s41467-020-15896-4

**Published:** 2020-05-01

**Authors:** Haruki Nishio, Diana M. Buzas, Atsushi J. Nagano, Koji Iwayama, Masayuki Ushio, Hiroshi Kudoh

**Affiliations:** 1 Center for Ecological Research, Kyoto University, Otsu, 520-2113 Japan; 2 Tsukuba-Plant Innovation Research Center and Faculty of Life and Environmental Sciences, University of Tsukuba, Tsukuba, 305-8572 Japan; 3 Faculty of Agriculture, Ryukoku University, Seta Oe-cho, Otsu, 520-2194 Japan; 4 Faculty of Data Science, Shiga University, Hikone, 522-8522 Japan; 5 PRESTO, Japan Science and Technology Agency, Kawaguchi, 332-0012 Japan; 6 Hakubi Center, Kyoto University, Yoshida-honmachi, Kyoto, 606-8501 Japan

**Keywords:** Epigenetics, Plant ecology, Plant molecular biology, Flowering

## Abstract

Natural environments require organisms to possess robust mechanisms allowing responses to seasonal trends. In *Arabidopsis halleri*, the flowering regulator *AhgFLC* shows upregulation and downregulation phases along with long-term past temperature, but the underlying machinery remains elusive. Here, we investigate the seasonal dynamics of histone modifications, H3K27me3 and H3K4me3, at *AhgFLC* in a natural population. Our advanced modelling and transplant experiments reveal that H3K27me3-mediated chromatin regulation at *AhgFLC* provides two essential properties. One is the ability to respond to the long-term temperature trends via bidirectional interactions between H3K27me3 and H3K4me3; the other is the ratchet-like character of the *AhgFLC* system, i.e. reversible in the entire perennial life cycle but irreversible during the upregulation phase. Furthermore, we show that the long-term temperature trends are locally indexed at *AhgFLC* in the form of histone modifications. Our study provides a more comprehensive understanding of H3K27me3 function at *AhgFLC* in a complex natural environment.

## Abstract (web summary)

The flowering regulator *FLC* shows upregulation and downregulation phases along with long-term past temperature in *Arabidopsis* *halleri*. Here, the authors reveal that H3K27me3-mediated chromatin regulation at *AhgFLC* provides the ability to respond to both the seasonal temperature trends and the perennial life cycle.

## Introduction

Organisms precisely operate seasonal biological programs even when short-term environmental fluctuations are intercalated into the long-term change^1–4^. Plants are suitable and ideal systems to study such programs. Not only are most plants long-lived (i.e. perennial) but also, unlike animals, plants are sessile and are composed of repeating, readily replaceable, units. Precision in timing the reproductive transition is critical in plants and it is potentiated by the ubiquitous flowering repressor *FLOWERING LOCUS C* (*FLC*) in Brassicaceae^5–9^. We previously showed that the flowering repressor *FLOWERING LOCUS C* (*FLC*) orthologue in perennial *Arabidopsis halleri* subsp. *gemmifera* (hereafter *AhgFLC*) responds to low temperatures of winter and controls perennial phenology — the *AhgFLC* expression pattern in a natural habitat is seasonal and can be explained by the past 6-week temperature^10^. Furthermore, temperatures greatly fluctuate in the natural environment of *A*. *halleri*, and often drop to the vernalisation-effective range (estimated to be less than 10.5 °C in *A*. *halleri*^10^; 0–16 °C in *A*. *thaliana*^3,11,12^) in both spring and autumn^1^. However, *AhgFLC* is reactivated in spring as if it is insensitive to the cold, although it is repressed in autumn^10^. Thus, we speculated that unexplored properties of *AhgFLC* regulation would permit the identification of long-term trends after short-term fluctuations were filtered out, allowing spring and autumn to be distinguished and preventing springtime *AhgFLC* repression.

*Arabidopsis thaliana FLC* (hereafter *AtFLC*) undergoes epigenetic silencing during vernalisation — a process in which plants become competent to flower after experiencing a period of prolonged cold of winter — in its life cycle as an annual plant. Prior to experiencing the cold, the transcription start site (TSS) of *AtFLC* is marked by active histone modifications such as histone H3 lysine 4 trimethylation (H3K4me3) and histone H3 lysine 36 trimethylation (H3K36me3), and the gene is actively transcribed^13,14^. In response to cold, *AtFLC* is repressed by at least two temperature-sensitive mechanisms in its regulatory network. In an early phase of cold exposure, the long non-coding antisense transcripts, *AtCOOLAIR*, are transcribed immediately downstream of the poly-A site of the *AtFLC* sense transcript, and *AtFLC* silencing is induced^15–17^; but see Helliwell et al.^18^. The H3K4me3 peak at the TSS of *AtCOOLAIR* correlates with the *AtCOOLAIR* transcription peak^14^. Cold exposure also induces the expression of *VERNALIZATION INSENSITIVE 3* (*VIN3*), the product of which contains a plant homeodomain (PHD)^19^. AtVIN3 and other PHD proteins associate with the core polycomb repressive complex 2 (PRC2) to form the PHD − PRC2 complex at the first intron of *AtFLC*^20^. The accumulation of PHD−PRC2 results in the nucleation of the repressive histone H3 lysine 27 trimethylation (H3K27me3) at a region close to the *AtFLC* TSS^13,14,20–23^. These repression processes are likely to be shared among species with annual and perennial life cycles within Brassicaceae^8,24,25^.

Distinct transcriptional outcomes at the *FLC* locus between annual and perennial plants occur on return to the warm after a prolonged cold period. In *A. thaliana*, H3K27me3 spreads over the gene body of *AtFLC*^14,20,22^ in a DNA replication-dependent manner^13,23^, and the silencing is maintained to allow the plants to flower under favourable long-day conditions^6^. The silencing of *AtFLC* lasts during the rest of the life cycle — flowering and fruiting — in annual *A. thaliana*^6^. In contrast, the silencing of *FLC* orthologues is transient in plants with perennial life cycles^8,10,24–26^. For example, the cold-induced repression of *PEP1*, an *FLC* orthologue in perennial *Arabis alpina*, and H3K27me3 accumulation at the locus are reset after return to the warm^8^. However, the detailed dynamics of H3K27me3 at the perennial *FLC* orthologues in both the upregulation and downregulation phases and the involvement of H3K27me3 in the gene regulation have remained elusive. We hypothesized that H3K27me3 at *AhgFLC* would provide a molecular basis for robust gene regulation.

A combination of time-series data and mathematical modelling has been successfully used to elucidate properties of the molecular mechanisms underlying the vernalisation process^3,4,10,22,27–29^. In this study, we combine high-frequency molecular phenology data and advanced modelling approaches to elucidate how H3K27me3 is involved in the robust seasonal regulation of *AhgFLC*. We measure the biweekly seasonal dynamics of *AhgFLC* H3K27me3 and H3K4me3 levels over 2 years in a natural population of *A. halleri* in Hyogo, Japan, using a chromatin immunoprecipitation (ChIP) protocol optimised for field samples^30^. One remarkable advantage of the long-term time-series data obtained in the natural plant population is that it can capture both the upregulation and downregulation phases of *AhgFLC* expression. The other advantage is that it can capture *AhgFLC* dynamics in the context of complex natural environments where little is known regarding gene functions^2,10,27,31–33^. The data sets provide a unique opportunity to apply a nonlinear time series analysis to elucidate the dynamics of chromatin modifications in a natural environment — we investigate the causality between *AhgFLC* histone modifications and expression via empirical dynamic modelling (EDM). We also examine the function of H3K27me3 in the seasonal regulation of *AhgFLC* using mathematical modelling. These approaches allow us to circumvent the absence of manipulative experimental systems and to determine how histone modifications at different regions in the same locus interact with each other to finally produce the long-term expression trend. Combined with transplant experiments, we find that H3K27me3 at the posterior region of the *AhgFLC* locus may contribute to the ratchet-like character of the *AhgFLC* system — reversible during the entire perennial life cycle but irreversible during the upregulation phase in spring.

## Results

### Seasonal dynamics of *AhgFLC* mRNA and histone modifications

**Fig. 1:**
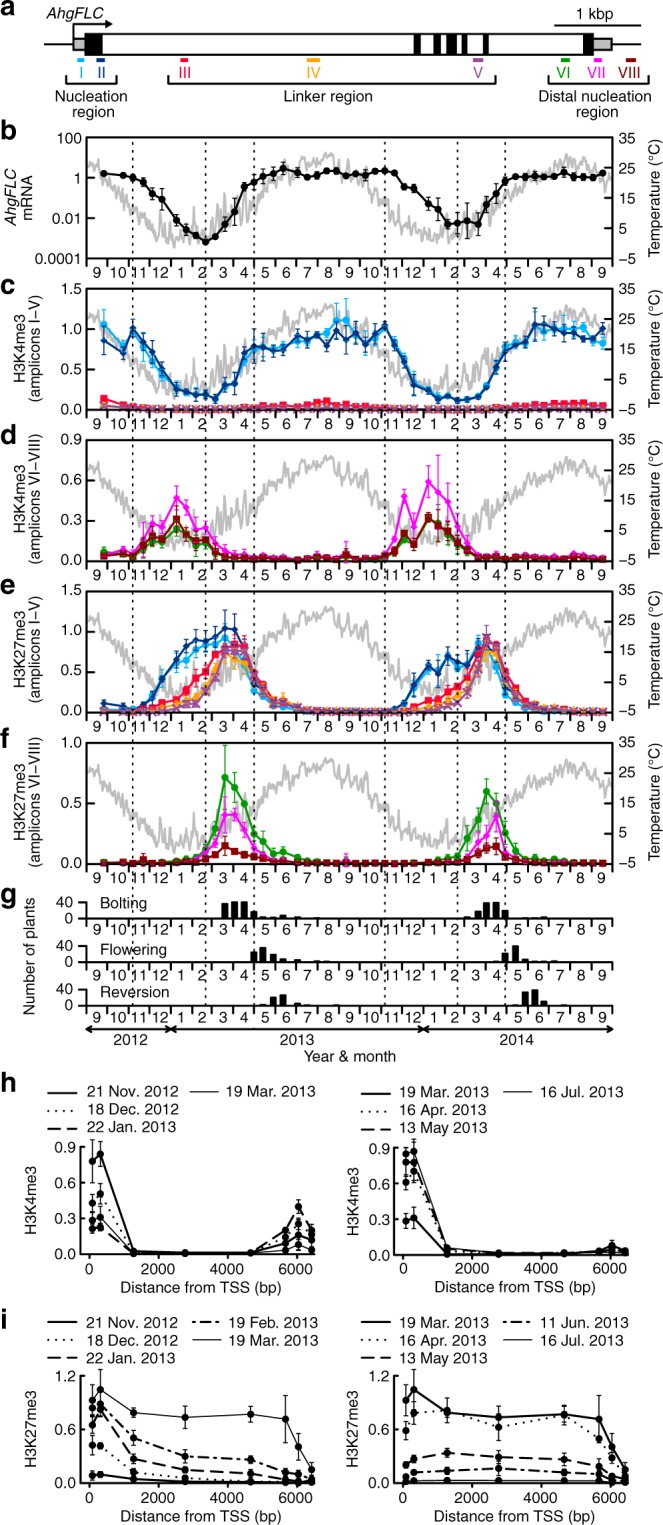
**Seasonal dynamics of*AhgFLC*mRNA and histone modification levels for 2 years in the natural habitat**. **a** Structure of the *AhgFLC* locus with untranslated regions (grey), exons (black) and introns (white); distribution of eight H3K4me3 and H3K27me3 ChIP amplicons in different colours and the definitions of the nucleation region, linker region, and distal nucleation region. **b**–**f** Relative quantification of the seasonal dynamics of *AhgFLC* mRNA (**b**), H3K4me3 at amplicons I–V (**c**), H3K4me3 at amplicons VI–VIII (**d**), H3K27me3 at amplicons I–V (**e**), and H3K27me3 at amplicons VI–VIII (**f**) in the natural population of *A. halleri* at 2-week intervals. The daily means of air temperature are plotted in grey. The colour code in **c**–**f** corresponds to that in **a**. **g** Flowering phenology of the study population (see Methods for the definition of the stages). Reversion, leaf formation at the reproductive shoot apical meristem. **h**, **i** The H3K4me3 (**h**) and H3K27me3 (**i**) levels against the distance from TSS along the *AhgFLC* locus in the downregulation (left) and upregulation (right) phases of expression. The qPCR data of *AhgFLC* are represented relative to those of *AhgACT2* (mRNA and H3K4me3) and *AhgSTM* (H3K27me3). The means and standard deviations of biological replicates are shown. *n* = 4 for mRNA, H3K4me3 and H3K27me3 at amplicons I–V. *n* = 3–4 (average, >3.9) for H3K4me3 and H3K27me3 at amplicons VI–VIII. For each replicate, a pool of leaves from ten plants (out of 40 plants) was analysed. Source data underlying Fig. 1b–g are provided as a Source Data file.

We quantitatively profiled *AhgFLC* steady-state mRNA, H3K4me3, and H3K27me3 levels across the entire locus at high temporal resolution over 2 years (Fig. 1a–f). We classified the *AhgFLC* locus into three regions based on previous reports of *A.thaliana*^14,22,23,28^, namely the nucleation region, the linker region, and the distal nucleation region (Fig. 1a). The nucleation region is known as the region registering quantitative increase in H3K27me3 with cold periods^13,14,22^. The distal nucleation region corresponds to the promoter and TSS regions of the antisense *AtCOOLAIR* transcripts, and the transient enrichment of H3K4me3 in this region correlates with the expression of *AtCOOLAIR*^14^. We confirmed the presence of *COOLAIR* transcripts in *A*. *halleri* (*AhgCOOLAIR*; Supplementary Fig. 1). We designated the region between the two nucleation regions as the linker region where transient H3K27me3 accumulation occurs, but H3K4me3 is absent^14^. We designed amplicons I and II in the nucleation region, amplicons III–V in the linker region, and amplicons VI–VIII in the distal nucleation region (Fig. 1a).

The seasonal dynamics of H3K4me3 at the nucleation region was similar to that of mRNA (Fig. 1b, c). At the distal nucleation region, H3K4me3 accumulated only during the decreasing phase of mRNA (November–March, Fig. 1d), which might be consistent with the putative role of *AhgCOOLAIR* in antagonising the *AhgFLC* sense transcription. The H3K27me3 levels at the nucleation region increased gradually from November to January–February and reached a plateau that lasted until March (Fig. 1e). The more distal a chromatin fragment was from the nucleation region, the longer was the delay in the increase of H3K27me3 levels (Fig. 1e, f). Especially, there was a prominent delay in the H3K27me3 accumulation between amplicons II and III. Therefore, we increased the ChIP resolution in this region during the critical period from November 2013 to March 2014 to reveal how H3K27me3 spreads within this region (amplicons A–D; Supplementary Fig. 2). The H3K4me3 level became lower toward amplicon III (Supplementary Fig. 2b, right). The first delay in the increase of H3K27me3 (Supplementary Fig. 2b, left) also lies within this region. Thus, while H3K27me3 spreads linearly, the speed of the spreading decreases in the vicinity of amplicon III, where the border of the H3K4me3 region lies. This feature was not captured in *A*. *thaliana* studies, where the features of the fast H3K27me3 spreading might be masked during swift shift from cold to warm temperatures^14,22,23^. In contrast to this gradual transmission of H3K27me3 from the nucleation region to the distal nucleation region in the H3K27me3 accumulation phase, the entire *AhgFLC* locus experienced a more synchronised decrease in the H3K27me3 levels, both temporally and quantitatively (Fig. 1e, f). We confirmed that these seasonal patterns were reproducible with a second set of validated^30^ reference genes (Supplementary Fig. 3). Consistent with the function of *AhgFLC* as a floral repressor, bolting, which is a phenological stage representing the initiation of reproduction, started immediately after *AghFLC* mRNA reached a minimum value (February–March) and was followed by flowering. Reversion to vegetative growth started in May after the complete activation of *AhgFLC* (Fig. 1g).

When we plotted the H3K4me3 levels against the distance from TSS along the *AhgFLC* locus, H3K4me3 at the nucleation region decreased from November to March and increased from March to July; H3K4me3 at the distal nucleation region was present from November to March and absent in other months (Fig. 1h and Supplementary Fig. 4a). For H3K27me3 levels, the nucleation near the TSS and spreading to the rest of the locus occurred from November to March, and the synchronised decrease across the entire locus occurred from March to July (Fig. 1i and Supplementary Fig. 4b). In conclusion, each of the three *AhgFLC* chromatin regions registers a specific pattern through the perennial phenology during the seasonal progression.

### Dependency of mRNA and histone modifications on temperature

**Fig. 2:**
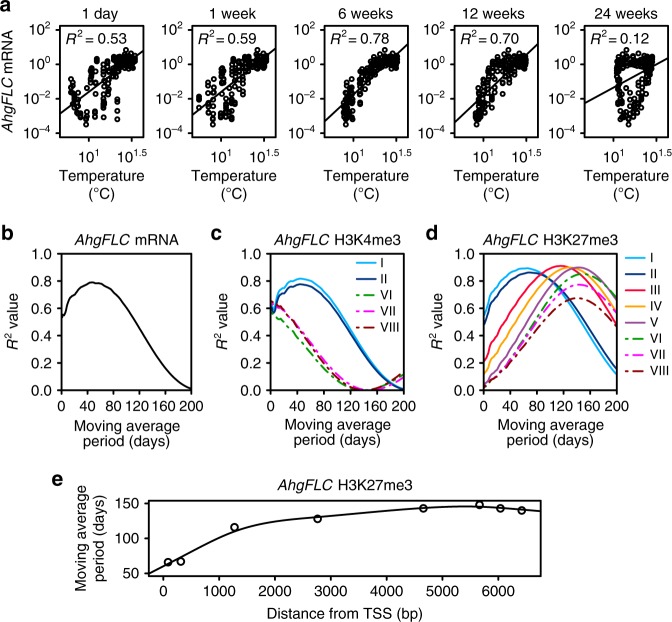
**Linear regression of*AhgFLC*mRNA and histone modification levels on the simple moving averages of past temperature**. **a** *AhgFLC* mRNA levels were plotted against the simple moving averages (SMAs) of the daily mean temperature with window lengths of 1 day, and 1, 6, 12, and 24 weeks. A regression line and coefficient of determination (*R*^2^) are shown in each diagram. **b**–**d** The results of linear regression analyses on the SMAs of the daily mean temperature with different window lengths. *R*^2^ values for *AhgFLC* mRNA (**b**), H3K4me3 (**c**) and H3K27me3 (**d**) levels are shown. Data are normalised against *AhgACT2* (mRNA and H3K4me3) and *AhgSTM* (H3K27me3) before regression analyses. **e** Plotted along the *AhgFLC* locus are the moving average periods of temperature that showed the highest *R*^2^ values for the H3K27me3 levels. Source data underlying Fig. 2a–d are provided as a Source Data file.

To determine the period of past temperature on which *AhgFLC* mRNA, H3K4me3, and H3K27me3 levels depend, we performed linear regression analyses against the simple moving averages (SMAs) of the daily mean temperature calculated for different window lengths (Fig. 2 and Supplementary Fig. 5). *AhgFLC* mRNA level was best explained by the temperature SMAs for the past 48 days, which is similar to the previously reported memory period of 6 weeks^10^ (Fig. 2a, b). *AhgFLC* H3K4me3 levels at the enriched amplicons were best explained by the temperature SMAs for the past 44 days at amplicons I and II — which is close to the past-temperature period for mRNA — and for the previous day at amplicons VI, VII and VIII (Fig. 2c). Thus, the past-temperature period for H3K4me3 was considerably shorter at the distal nucleation region than at the nucleation region, suggesting that these regions might possess different thermosensors. This result allowed us to predict that *AhgCOOLAIR* would respond to past temperature for shorter period than the sense *AhgFLC* transcript (also see the results of EDM). At all amplicons, the past-temperature periods for H3K27me3 were longer than those for H3K4me3 and mRNA (Fig. 2b–d). *AhgFLC* H3K27me3 levels were best explained by the temperature SMAs for the past 66, 67, 116, 128, 143, 148, 143 and 140 days at amplicon I, II, III, IV, V, VI, VII and VIII, respectively (Fig. 2d). Therefore, the past-temperature period for H3K27me3 positively depended on the distance from TSS (Fig. 2e). We performed the SMA analyses for the daily maximum and minimum temperatures and obtained similar results to those of the daily mean temperature (Supplementary Fig. 5b–g). In conclusion, the nucleation region and the distal nucleation region respond to different lengths of past temperature, as if they represent distinct functional units.

### Interrelation between *AhgFLC* mRNA and histone modifications

**Fig. 3:**
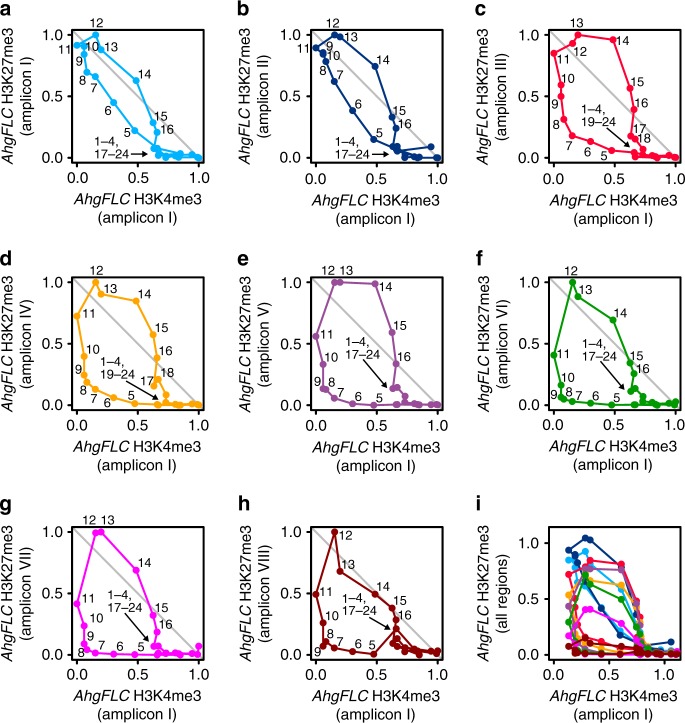
**Phase differences between the seasonal dynamics of*AhgFLC*** H3K27me3 and H3K4me3 for the first year measurement. **a**–**h** Lissajous curves, i.e. time-series trajectories of two sets of cyclical values, delineated by plotting *AhgFLC* H3K4me3 at amplicon I on the horizontal axis and H3K27me3 at amplicon I (**a**), amplicon II (**b**), amplicon III (**c**), amplicon IV (**d**), amplicon V (**e**), amplicon VI (**f**), amplicon VII (**g**), and amplicon VIII (**h**) on the vertical axis. *AhgFLC* H3K27me3 and H3K4me3 levels are shown as relative values, setting the minimum level to 0 and the maximum level to 1 in **a**–**h**. The numbers next to the data points are chronological ordinals (1: 25 September 2012, 24: 10 September 2013). **i** Lissajous curves drawn using the absolute values for all tested regions. The colour code corresponds to that in Fig. 1a. Data are normalised against *AhgSTM* (H3K27me3) and *AhgACT2* (H3K4me3) and shown as the means of four biological replicates. Source data are provided as a Source Data file.

To infer how *AhgFLC* mRNA, H3K4me3 and H3K27me3 dynamics might interrelate, we performed two analyses. First, we used Lissajous curves, which are time-series trajectories of two sets of cyclical values delineated by plotting one on the horizontal axis and the other on the vertical axis^34^ (Supplementary Fig. 6), to determine the temporal sequences of events. The shapes and trajectory directions of Lissajous curves represent the degree and direction of the phase differences between the two values, respectively^34^. We found that the Lissajous curves between H3K4me3 at amplicon I and H3K27me3 at all amplicons were elliptical, and the time-series trajectories of the Lissajous curves rotated clockwise, representing delayed H3K27me3 dynamics relative to H3K4me3 dynamics (Fig. 3 and Supplementary Fig. 7). The ellipses expanded at amplicons III–VIII relative to those at amplicons I and II, indicating a longer delay for H3K27me3 (Fig. 3 and Supplementary Fig. 7). The Lissajous curves between H3K27me3 and mRNA were elliptical and similar to those between H3K27me3 and H3K4me3 (Supplementary Fig. 8). The trajectories of the Lissajous curves between H3K4me3 and mRNA overlapped during their upward and downward phases at amplicons I and II, suggesting little phase difference in their seasonal dynamics (Supplementary Fig. 9). In contrast, H3K4me3 and mRNA exhibited elliptical shapes at amplicons VI–VIII, and the time-series trajectories of the Lissajous curves rotated anticlockwise, representing the phase advances of H3K4me3 at these amplicons relative to mRNA (Supplementary Fig. 9).

**Fig. 4:**
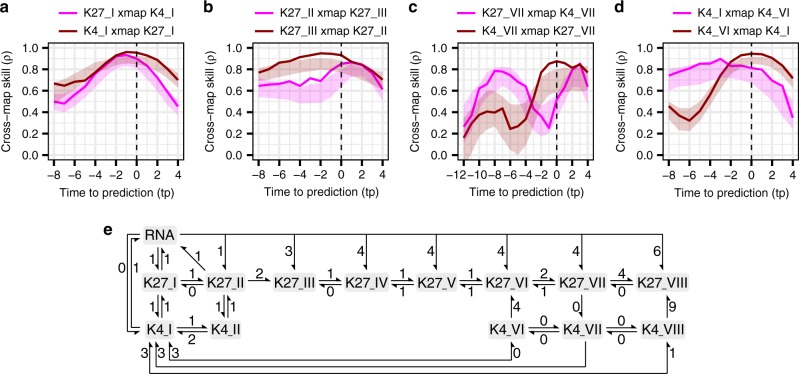
**Empirical dynamic modelling of*AhgFLC*histone modification and mRNA levels**. **a**–**d** Convergent cross-mapping (CCM) between *AhgFLC* H3K27me3 at amplicon I (K27_I) and K4_I (**a**), between K27_II and K27_III (**b**), between K27_VII and K4_VII (**c**), and between K4_I and K4_VI (**d**). The cross-map skill (*ρ*) is shown as the function of time to prediction (tp). In the top keys, e.g. ‘K27_I xmap K4_I’ represents that K27_I cross-map (or cross-predict) K4_I, indicating that the state of K4_I is predicted using the state of K27_I. Because CCM explores the signature of a causal variable in an effect variable, this prediction measures the effect of K4_I on K27_I. Solid lines represent the cross-map skill (*ρ*), and shaded regions represent the 95% intervals of 100 seasonal-surrogate time series. **a** Bidirectional causality between K27_I and K4_I was detected since both variables were the best predicted by each other at negative tp. **b** Unidirectional causality from K27_II to K27_III was detected since the best forecasting skill occurred at tp ≤0 only for ‘K27_III cross-maps K27_II’. **c** Unidirectional causality from K27_VII to K4_VII was detected for the same reason as in **b**. **d** Bidirectional causality between K4_I and K4_VI was detected for the same reason as in **a**. **e** The causal network of *AhgFLC* H3K27me3, H3K4me3, and mRNA, illustrated based on the results of CCM. Arrows represent the directions of causality. The numbers next to the arrows represent the time lags (1 time lag = 2 weeks). Source data are provided as a Source Data file.

Next, to investigate the causality between *AhgFLC* mRNA, H3K4me3 and H3K27me3, we performed an EDM causality test called convergent cross-mapping (CCM)^35,36^. First, we determined the optimal embedding dimensions by using simplex projection^35,37^ (Supplementary Fig. 10), and then applied CCM to determine the causality between the variables (Fig. 4 and Supplementary Figs. 11, 12). The results of CCM showed that the best cross-map skill (forecasting accuracy measured using correlation coefficients, *ρ*, which can be an index of causality strength; see Methods for more details)^35,38^ between H3K27me3 and H3K4me3 at amplicon I occurred at a lag of two weeks (i.e. the parameter tp = −1 in the EDM function) for both directions (Fig. 4a). The cross-map skills at the time lag were significantly higher than those of seasonal-surrogate time series for both directions (Fig. 4a and Supplementary Fig. 11a). These results suggest that the bidirectional causal interactions between H3K27me3 and H3K4me3 at amplicon I occur with the time delay of approximately two weeks. The same was true for the cross-mappings between H3K27me3 and mRNA (Supplementary Figs. 11b, 12b). In addition, we detected a unidirectional causality from H3K27me3 at amplicon II to that at amplicon III with a time delay of approximately four weeks (i.e. tp = −2; Fig. 4b and Supplementary Fig. 11c), implying that the backward propagation of the modification from amplicon III to II might be retarded.

At the distal nucleation region, the CCM results indicated causality between H3K27me3 and H3K4me3, from H3K27me3 to H3K4me3 at amplicon VII, and from H3K4me3 to H3K27me3 at amplicons VI and VIII (Fig. 4c and Supplementary Figs. 11a, 12a). We expected the causality from H3K27me3 to H3K4me3 at amplicon VII to be direct because no time delay was present (tp = 0). In contrast, the very large time delays in the causality from H3K4me3 to H3K27me3 at amplicons VI and VIII (tp = −4 for amplicon VI, tp = −9 for amplicon VIII) might predict the indirect causality. In addition, a causal link of H3K4me3 between the nucleation region (amplicons I and II) and the distal nucleation region (amplicons VI–VIII) was detected (Fig. 4d and Supplementary Figs. 11d, 12d). These results suggest that the two nucleation regions represent distinct regulatory units where H3K27me3 and H3K4me3 interact and that these units are causally linked via the H3K4me3 interaction.

We illustrated the entire causal network of mRNA, H3K4me3, and H3K27me3 based on the CCM results (Fig. 4e). The H3K27me3 interactions between adjacent amplicons were consistent with the previously assumed linear propagation of histone modifications between adjacent nucleosomes^39^ and did not contradict the chromatin looping-driven spreading model^40,41^.

Next, we detected causality from temperature to each of mRNA, H3K4me3, and H3K27me3, indicating that temperature affects these variables either directly or indirectly (Supplementary Fig. 13). Consistent with the results of the linear regression analyses against the temperature SMAs (Fig. 2c), we detected a direct causality (tp = 0) from temperature to H3K4me3 at the distal nucleation region (Supplementary Fig. 13d, g, h). Taking into consideration the results of the Lissajous curve analyses, change in the H3K4me3 level at the distal nucleation region would be the first event driven by an external driver (i.e. temperature) in the seasonal *AhgFLC* regulation. Given that we quantified the histone modification levels using pooled samples from multiple individuals (see Methods for more details), the common environmental driver would cause a coupled response between multiple plant individuals within the population.

### Function of the H3K27me3 and H3K4me3 interaction

**Fig. 5:**
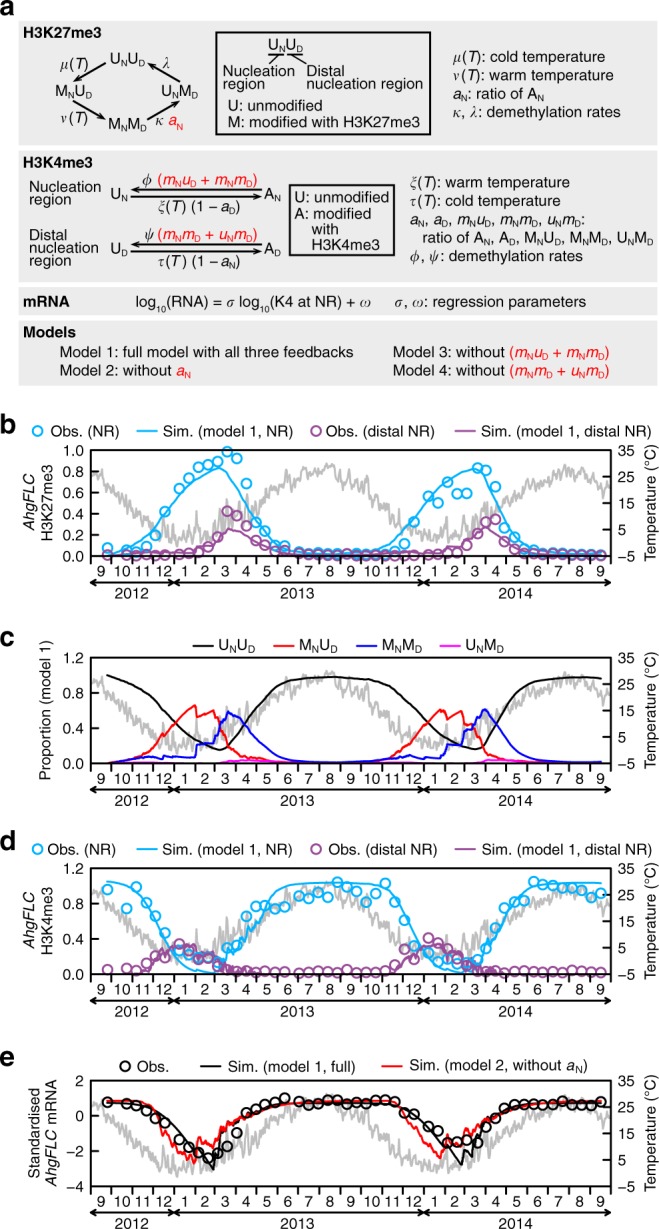
**Mathematical modelling of*AhgFLC*histone modification and mRNA levels**. **a** Schematic representation of the transitions between the four H3K27me3 states and the transitions of the H3K4me3 states at the two nucleation regions. The transition of the H3K27me3 states between U_N_U_D_, M_N_U_D_, M_N_M_D_ and U_N_M_D_ at the *AhgFLC* locus was assumed to be unidirectional. The U_N_U_D_ → M_N_U_D_ and M_N_U_D_ → M_N_M_D_ transitions are assumed to be induced by cold and warm temperatures represented by the temperature functions *μ*(*T*) and *ν*(*T*), respectively. H3K4me3 at the nucleation region and the distal nucleation region are assumed to accumulate in response to warm [*ξ*(*T*)] and cold [*τ*(*T*)] temperatures, respectively. Three feedback regulations between H3K27me3 and H3K4me3 [*a*_N_, (*m*_N_*u*_D_ + *m*_N_*m*_D_), (*m*_N_*m*_D_ + *u*_N_*m*_D_); red letters] are assumed. *AhgFLC* mRNA level is modelled by a linear regression with the H3K4me3 level at the nucleation region (K4 at NR). The definitions of all parameters are described in Supplementary Table 2. **b** The observed (obs.) and simulated (sim.; model 1) H3K27me3 levels at the nucleation region (NR; blue) and the distal nucleation region (distal NR; purple) of *AhgFLC*. **c** The predicted dynamics of the proportion of the four H3K27me3 states at *AhgFLC* in model 1. **d** The observed (obs.) and simulated (sim.; model 1) H3K4me3 levels at the nucleation region (NR; blue) and the distal nucleation region (distal NR; purple) of *AhgFLC*. **e** *AhgFLC* mRNA levels modelled by a linear regression are compared between model 1 (full model with all three feedbacks; black) and model 2 that lacks the feedback effect of H3K4me3 on the M_N_M_D_ → U_N_M_D_ transition (red) and are shown with the observed values. In **b**–**e**, the daily means of air temperature are plotted in grey. The observed values are shown as the means of four biological replicates and are represented by circles, whereas the simulated values are represented by lines. Source data are provided as a Source Data file.

To explore the function of H3K27me3 at *AhgFLC* in a natural environment, we constructed a mathematical model predicting the H3K27me3, H3K4me3 and mRNA dynamics at the tissue level from natural temperatures. We modelled H3K27me3 and H3K4me3 at the two nucleation regions because these regions are the distinct regulatory units where H3K27me3 and H3K4me3 interact (Fig. 4 and Supplementary Figs. 11, 12). We described the H3K27me3 states using combinations of unmodified (U) and modified (M) states (U_N_U_D_, M_N_U_D_, M_N_M_D_ and U_N_M_D_; the first letter with a subscript (N) indicates the state of the nucleation region, while the second letter with a subscript (D) indicates the state of the distal nucleation region). The probabilities that an *AhgFLC* locus in a single cell is in these four states were designated as *u*_N_*u*_D_, *m*_N_*u*_D_, *m*_N_*m*_D_ and *u*_N_*m*_D_, respectively (*u*_N_*u*_D_ + *m*_N_*u*_D_ + *m*_N_*m*_D_ + *u*_N_*m*_D_ = 1), which can be considered as the proportion of cells in these states in a tissue composed of a large number of cells. We derived differential equation models from stochastic models (Supplementary Note 1) without violating the assumption that *FLC* is regulated in cis^17,42^. For ease of derivation of differential equation models, we assumed that the H3K27me3 state, the H3K4me3 state at the nucleation region, and the H3K4me3 state at the distal nucleation region are stochastically independent (see the Methods section and Supplementary Note 1 for the evaluation of this assumption). We included the U_N_M_D_ state because we observed that H3K27me3 at the nucleation region began to decrease slightly (1–2 time points) earlier than that at the other regions, which was especially clear in the measurements conducted during the second year (Supplementary Fig. 7). Based on the seasonal dynamics of H3K27me3 at *AhgFLC* (Fig. 1e, f), we assumed unidirectional circular transitions between the four states: U_N_U_D_ → M_N_U_D_ → M_N_M_D_ → U_N_M_D_ → U_N_U_D_ (Fig. 5a). We also assumed that the U_N_U_D_ → M_N_U_D_ and M_N_U_D_ → M_N_M_D_ transitions are induced by cold and warm temperatures, respectively, in accordance with the vernalisation process in *A*. *thaliana*^13,14,22,23^. We described the H3K4me3 states at the two nucleation regions separately by unmodified (U) and modified (A) states because different sets of thermosensors were assumed to be present between these regions (Fig. 2c). We assigned U_N_ and A_N_ for the nucleation region [the proportions of cells: *u*_N_ and *a*_N_, respectively (*u*_N_ + *a*_N_ = 1)], and U_D_ and A_D_ for the distal nucleation region [the proportions of cells: *u*_D_ and *a*_D_, respectively (*u*_D_ + *a*_D_ = 1); Fig. 5a]. Based on the results of CCM (Fig. 4) and previous models^22,28,43,44^, we assumed three feedback regulations between H3K27me3 and H3K4me3 [*a*_N_, (*m*_N_*u*_D_ + *m*_N_*m*_D_), and (*m*_N_*m*_D_ + *u*_N_*m*_D_); red letters in Fig. 5a]. *AhgFLC* mRNA level was modelled by linear regression with the H3K4me3 level at the nucleation region (Fig. 5a). We compared the full model (model 1 with all three feedbacks) and the models without each of the three feedbacks (model 2, 3 and 4, respectively; Fig. 5a).

In model 1, the simulated *AhgFLC* H3K27me3 levels agreed well with the observed data (Fig. 5b), indicating that our simple model explains the major dynamics over multiple weeks. During the cold season (November–February), the M_N_U_D_ state was predominant, and M_N_M_D_ showed a slower increase depending on the M_N_U_D_ increase and intermittent warm temperatures (Fig. 5c) — temperature was occasionally higher than 10 °C even in the middle of winter (Supplementary Fig. 3a). In response to the temperature increase after March, M_N_M_D_ became predominant and transitioned swiftly into U_N_U_D_, without U_N_M_D_ ever reaching a high level (Fig. 5c), indicating that H3K27me3 is removed almost simultaneously at the two nucleation regions. In model 1, the simulated *AhgFLC* H3K4me3 levels agreed well with the observed data (Fig. 5d).

Next, we evaluated how the mutual regulation of H3K27me3 and H3K4me3 affects the seasonal *AhgFLC* mRNA dynamics by comparing the full model (model 1) with the other three models. First, model 1 was compared with model 2, which lacks the feedback effect of H3K4me3 on the transition from M_N_M_D_ to U_N_M_D_, assuming that the transition occurs at a constant rate and replacing ‘*κ a*_N_’ with ‘*κ*’ (Fig. 5a). Model 1 explained the long-term mRNA dynamics in the natural environment (Fig. 5e). However, model 2 failed to recapitulate the mRNA dynamics during autumn−spring, and an earlier decrease during December−January and an earlier increase during February−March were observed (Fig. 5e). In the model without the feedback from H3K27me3 to H3K4me3 at the nucleation region (model 3) and the distal nucleation region (model 4), the mRNA dynamics were similar to that in model 1 (Supplementary Fig. 14). These results suggested that the feedback from H3K4me3 to H3K27me3 at the nucleation region is required for *AhgFLC* mRNA to respond to long-term temperature trends.

### Ratchet function of H3K27me3 at the distal nucleation region

**Fig. 6:**
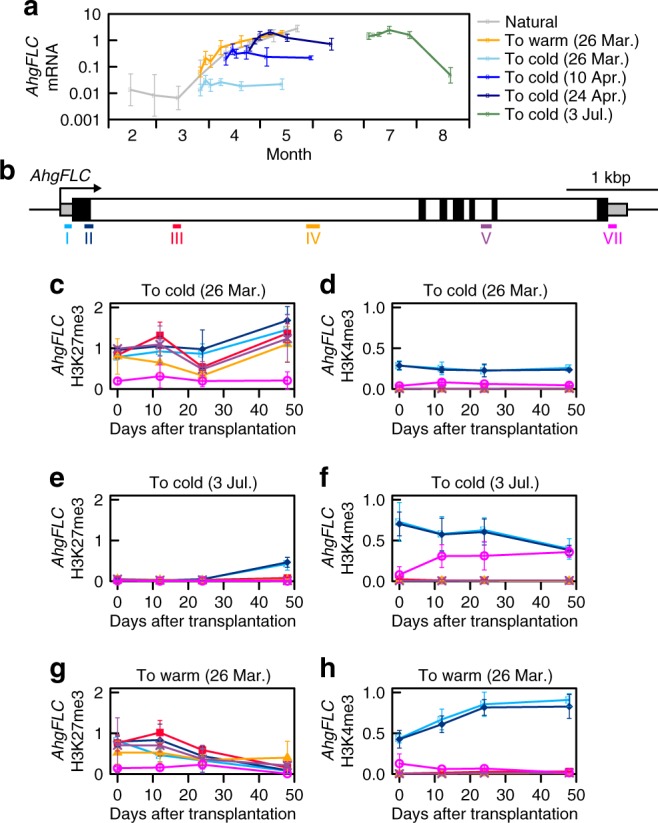
**Lack of*AhgFLC*cold responses in spring and the role of H3K27me3 at the distal nucleation region**. **a** *AhgFLC* mRNA levels in naturally growing plants and in plants transferred to warm or cold conditions on the indicated dates. Data are normalised against *AhgACT2*. The means and standard deviations of biological replicates are shown. *n* = 4 for naturally growing plants; *n* = 3 for transplanted plants. **b** The distribution of six H3K27me3 and H3K4me3 ChIP amplicons along the *AhgFLC* locus is shown in different colours. **c**, **d** *AhgFLC* H3K27me3 (**c**) and H3K4me3 (**d**) levels after transfer to cold on 26 March. **e**, **f** *AhgFLC* H3K27me3 (**e**) and H3K4me3 (**f**) levels after transfer to cold on 3 July. **g**, **h** *AhgFLC* H3K27me3 (**g**) and H3K4me3 (**h**) levels after transfer to warm on 26 March. Data are normalised against *AhgSTM* (H3K27me3) and *AhgACT2* (H3K4me3). In **c**–**h**, the colour code corresponds to that in **b**, and the means and standard deviations of biological replicates are shown (*n* = 3). For each replicate, a pool of leaves from two plants (out of six plants) was analysed. Source data underlying Fig. 6a, c–h are provided as a Source Data file.

Next, we investigated the mechanism required to distinguish spring from autumn despite the similarity of temperature profiles. To reveal whether *AhgFLC* transcription is insensitive to cold in spring, we transferred naturally growing plants to a constant 4 °C chamber during the springtime upregulation of the gene. In three-time transplantations in March and April, the mRNA level did not decrease over a period of 48 days, indicating that *AhgFLC* is insensitive to the cold (Fig. 6a). The entire *AhgFLC* locus was covered by H3K27me3 in spring (Fig. 1e, f), and a direct causality from H3K27me3 to H3K4me3 was detected at amplicon VII (Fig. 4c). Thus, we speculated that H3K27me3 at amplicon VII maintains the low level of H3K4me3 at amplicon VII (thus the repressed state of the antisense transcription) to prevent unseasonal downregulation of *AhgFLC*. In support of this idea, in the March transfer when the locus was fully covered by H3K27me3 (Fig. 6b, c), the H3K4me3 levels at the nucleation region (amplicons I and II) and the distal nucleation region (amplicon VII) remained steady (Fig. 6d). In contrast, in plants from the July transfer when the locus was devoid of H3K27me3 (Fig. 6e), the mRNA level decreased, indicating that *AhgFLC* eventually recovered the sensitivity to long-term cold (Fig. 6a). During this response, the H3K4me3 level at the nucleation region did not show a clear decrease, but that at the distal nucleation region (around TSS of antisense transcript, *AhgCOOLAIR*) increased (Fig. 6f). Interestingly, *AhgFLC* mRNA level increased for the first 3–4 days after the transfer to the cold chamber on 26 March and 10 April, and for the first 12 days after the cold transfer on 24 April and 3 July (Fig. 6a). This indicates that the *AhgFLC* locus retains the memory of warm temperatures for a period correlating with the amount of warm that plants have experienced. When plants were transferred to a 24 °C chamber in March for the control treatment, *AhgFLC* mRNA level gradually increased in a similar manner to that under the natural condition (Fig. 6a). During this transfer to the warm, the H3K27me3 levels decreased at all the amplicons, and the H3K4me3 level increased at the nucleation region (Fig. 6g, h), which was similar to the dynamics observed in the natural population (Fig. 1c–f). Taken together, these results suggest that H3K27me3 stabilises the H3K4me3 level at the distal nucleation region, and prevents unseasonal downregulation of *AhgFLC* in spring despite the presence of temperature fluctuations.

## Discussion

The sessileness and perenniality of our study species enabled us to obtain high-frequency molecular phenology data for gene expression and histone modifications in a natural environment. By integrating the data, advanced modelling approaches, and manipulative experiments, we showed that H3K27me3-mediated chromatin regulation at *AhgFLC* provides two properties that are required for robust gene regulation in a fluctuating natural environment.

The first property is the ability to filter out short-term temperature fluctuations by the feedback regulation from H3K4me3 to H3K27me3 at the nucleation region. The seasonal dynamics of *AhgFLC* H3K27me3 were delayed relative to those of mRNA and H3K4me3, and the CCM results indicated a causality from mRNA and H3K4me3 to H3K27me3 in addition to the typical consequence of H3K27me3 on transcriptional repression. Thus, the regulation of H3K27me3 depends on transcription at an endogenous *FLC* locus, consistent with the finding that experimental manipulations of transcription triggered changes in the H3K27me3 level at the *AtFLC* transgene^45^. Our mathematical modelling showed that the feedback regulation from H3K4me3 to H3K27me3 allows *AhgFLC* expression to follow the long-term temperature trends. Recent studies have shown that multiple thermosensors are distributed in the regulatory unit of *AtFLC* as well as that of *AtVIN3* (refs. ^3,4^). Our model that captured *AhgFLC* dynamics also had four temperature inputs. In this study, we analysed H3K4me3 as an active modification at the *AhgFLC* locus to model dynamics at the two nucleation regions. Given that H3K36me3 has been reported to be antagonistic to H3K27me3 at all locations across *AtFLC*^14^, modelling the seasonal dynamics of H3K36me3 at *AhgFLC* should be addressed in future studies.

The second property is the ratchet-like character of the *AhgFLC* system, i.e. reversible in the entire perennial life cycle, but irreversible during upregulation phase. Because upregulation of *FLC* after winter is essential for a perennial life cycle, this irreversibility is relevant to perennial rather than annual species of *Arabidopsis*. The transplant experiments showed that *AhgFLC* expression becomes insensitive to prolonged cold when the entire locus is covered by H3K27me3 in spring. Although the H3K4me3 level at the distal nucleation region increased after the plants were transferred to a cold condition in summer, the level remained low after the cold transfer in spring. We confirmed the presence of *AhgCOOLAIR* transcripts of which the TSS/promoter corresponds to the distal nucleation region. Thus, our results imply that the spreading of H3K27me3 to the distal nucleation region represses *AhgCOOLAIR* and renders the sense transcription insensitive to the prolonged cold in spring. In contrast, when H3K27me3 is absent from the distal nucleation region during summer–winter, H3K4me3 at the region responds to cold within a short period. Interestingly, the timing of H3K27me3 accumulation at the distal nucleation region of *AhgFLC* corresponds to that of *AhgFLC* upregulation (compare Fig. 1b, f), which might repress unseasonal *AhgCOOLAIR* transcription in spring. In any case, our data have suggested a role for H3K27me3 at the *FLC* posterior region — that allows the irreversible upregulation of perennial *FLC* in the presence of springtime temperature fluctuations — in addition to the maintenance of the silenced *FLC* state in annual plants^13,14,20,22,23^.

Furthermore, we have shown that long-term seasonal trend of temperature is indexed locally at *AhgFLC* in the form of histone modifications. The H3K27me3 levels were correlated with longer past temperatures when they were measured at a greater distance from the TSS along the locus. This suggests that temporal environmental information is transformed into the spatial pattern of H3K27me3 accumulation along the locus. The collinearity between seasons and consecutive chromatin segments observed at *AhgFLC* are remarkably similar to the collinearity between the spatiotemporal sequences of gene activation and the physical order of the genes observed at the *HOX* gene loci in *Drosophila*^46,47^.

In conclusion, our study provides a more comprehensive understanding of H3K27me3 function at the *AhgFLC* locus to achieve the robust seasonal expression in a complex natural environment. Our findings provide the basic background for future studies on ecological and agricultural aspects, e.g. predicting phenological shifts and developing robust crops in a changing climate. In animals, H3K27me3 mediates long-term gene regulation in organismal responses that occur over weeks, e.g. development^48–50^ and carcinogenesis^51–53^. Therefore, time-series analyses on a broad range of species — not only Brassicaceae but also other plant families and animals — will reveal the evolutionary conservation of the H3K27me3 functions in robust gene regulation under natural conditions.

## Methods

### Field sampling of leaves for seasonal analysis

Field sampling of leaves was conducted at 2-week intervals for 2 years (from 25 September 2012 to 16 September 2014) in a natural population of *A. halleri* at the Omoide-gawa River, Naka-ku, Taka-cho, Hyogo Prefecture, Japan (35°06′ N, 134°55′ E; altitude 190–230 m). On each date, sampling started at 12.00 and was completed within one and a half hours (by 13.30).

To evaluate the dynamics of histone modifications at the level of the entire population, we sampled 40 leaves from 40 plants (1 leaf per plant) and made four ChIP biological replicates by pooling ten leaves for each replicate (1 g per replicate) because a large amount of leaf tissue was needed for the ChIP experiments. Sampling was conducted along a stream in a sampling area of ~20 m × 300 m. On each sampling date, the area was divided into 10 sections, and 4 plants were sampled from each section (>2 m between plants). Pooled 10 leaves were from ten sections (one leaf per section). Forty plants were newly selected on each sampling date. The total number of seasonal ChIP samples was 200 (50 time points × 4 replicates).

We analysed leaf samples because they are the most accessible plant tissues that are available year-round in the field^10^. One fully expanded young leaf was harvested from each plant (~0.1 g per plant). During the vegetative phase, we sampled rosette leaves. During the flowering period (from 30 April 2013 to 11 June 2013 for the first year and from 28 April 2014 to 10 June 2014 for the second year), we sampled cauline leaves (leaves on a flowering stem) because the rosette leaves had started to senesce after the reproductive transition. Once all four replicates were obtained, samples were fixed with 1% formaldehyde in PBS in the field. Vacuum infiltration was conducted twice, for 5 min each, at ambient temperature. To quench the cross-linking reaction, glycine was added to a final concentration of 125 mM and vacuum infiltration was conducted for an additional 5 min.

For RNA samples, we obtained four biological replicates from additional individuals on each sampling date. One small young leaf (~0.01 g) was harvested for each replicate. Harvested leaves were preserved in RNA*later* Stabilization Solution (AM7021; Thermo Fisher Scientific, Waltham, MA, USA), kept on ice during transfer to the laboratory, and then stored at −20 °C.

### ChIP-qPCR

For ChIP experiments, we modified the protocol of Gendrel et al.^54^ as described below. Chromatin was extracted using extraction buffer 1–3, and sonicated eight times for 15 s each using a Q700 Sonicator (Qsonica, Newtown, CT, USA) at 10% power output. After centrifugation, the supernatant was diluted in ChIP dilution buffer (up to 3.0 ml per 1 g sample). For pre-clearing of this chromatin lysate, it was incubated with Dynabeads Protein G (Thermo Fisher Scientific) at 4 °C for 1 h with rotation. The chromatin lysate was then incubated with antibody for 5 h. Antibody dilutions were as follows: 1:500 for anti-H3K27me3 (07-449; Millipore, Billerica, MA, USA) and anti-H3K4me3 (07-473; Millipore) and 1:1000 for anti-histone H3 (ab1791; Abcam, Cambridge, UK). By the incubation with Dynabeads Protein G at 4 °C for 2 h, the immune complexes were collected. The beads were then washed with low salt, high salt, LiCl, and TE buffers in this order. By incubating the washed beads with elution buffer at 65 °C for 15 min, immunoprecipitated chromatin was eluted. Each sample was then heated at 65 °C for 12 h to reverse formaldehyde cross-linking, and incubated wih proteinase K at 45 °C for 1 h. After phenol/chloroform extraction, DNA was ethanol-precipitated, and resuspended in 50 μl of TE buffer. qPCR was performed in duplicate by using the appropriate primers (Supplementary Table 1). To collect data, 7300 System SDS Software v1.3 was used. We analysed eight regions along the *AhgFLC* locus: amplicons I–VIII. Amplicons I and II were selected within the previously defined nucleation region^14,22,23,28^; amplicons III–V were selected within the gene body with a distance of more than 1 kbp between them; amplicons VI–VIII were selected around the 3′ end of the locus. The absolute amount of H3K4me3/H3K27me3 ChIP DNA, expressed as percentage of input, was divided by the absolute amount of H3 ChIP DNA, expressed as percentage of input at the same region. In addition, as internal controls, *AhgSTM* and *AhgFUS3* were used for H3K27me3 ChIP, and *AhgACT2* and *AhgPP2AA3* were used for H3K4me3 ChIP^30^. Both *AhgFLC* H3K27me3 and H3K4me3 levels are presented on a linear scale.

### RNA extraction and RT-qPCR

RNA was extracted using an RNeasy Plant Mini Kit (Qiagen, Hilden, Germany) and quantified using Qubit Fluorometer and Qubit RNA HS Assay Kits (Thermo Fisher Scientific). cDNA was synthesised using a High-Capacity cDNA Reverse Transcription Kit (Thermo Fisher Scientific). qPCR was performed in duplicate by using the appropriate primers (Supplementary Table 1). To collect data, 7300 System SDS Software v1.3 was used. Normalisation was done using a standard cDNA sample produced from laboratory-grown non-vernalised plants. *AhgFLC* mRNA level was normalised with the mRNA level of either *AhgACT2* or *AhgPP2AA3* (ref. ^30^) and is presented on a common logarithmic scale.

### Plant phenology

On each sampling date for the seasonal analysis, we recorded the growth stages of the 40 plants whose leaves were harvested for ChIP samples. During the reproductive phase, we classified the plants into three sequential stages, i.e. bolting, flowering, and reversion, and recorded the number of plants in each stage. Bolting was defined as the stage when flowering stems were longer than 5 mm but did not present open flowers. Flowering was defined as the stage with visible white petals after the flowers had opened. Reversion was defined as the stage when leaves formed at the tip of the bolt at the end of flowering. During reversion, plants form aerial rosettes at the most distal end of the flowering stem and then generate roots at the same node, facilitating the establishment of vegetative (clonal) offspring. We defined the generation of roots at the aerial rosettes as the start of the next clonal generation and the end of the reversion stage.

### Cloning of *AhgCOOLAIR*

RNA extraction was performed as described above from the leaves of mature *A*. *halleri* plants grown for 7 and 21 days at 4 °C. We applied two cold periods to capture the transient expression of putative *AhgCOOLAIR*. Five micrograms of RNA were used for SuperScript III (18080044; Thermo Fisher Scientific) cDNA synthesis of polyA transcripts. PCR amplification was performed on 1:50 cDNA dilution using TaKaRa Ex Taq (RR001A; Takara Bio, Kusatsu, Shiga, Japan) using the primers listed in Supplementary Table 1. PCR products from the two stages were pooled, cleaned using a QIAEX II Gel Extraction Kit (20021; Qiagen), and then cloned into pGEM-T-Easy-Vector-Systems (A1360; Promega, Madison, WI, USA). Individual clones were sequenced by Eurofins Genomics (Tokyo, Japan).

### Dependency of mRNA and histone modifications on temperature

For linear regression analyses, we used the air temperature data recorded by the Japan Meteorological Agency at the meteorological station in Nishiwaki, Hyogo Prefecture, Japan (35°00′ N, 135°00′ E; altitude 72 m), which is the nearest station to our field site. To represent the past-temperature trend, we calculated the SMAs of the daily mean, maximum, or minimum air temperature with different window lengths. For example, for the 1-d SMA, we used the temperature of the day before each sampling date, and for the 1-week SMA, we averaged the temperature for the seven days before each sampling date. By using the temperature SMA as the explanatory variable and either *AhgFLC* H3K27me3, H3K4me3, or mRNA level as the response variable, we performed linear regression analyses by using the lm function of R v3.2.1. The coefficient of determination (*R*^*2*^ value) was calculated to estimate the goodness-of-fit of the linear regression models for the observed data. All variables were transformed into logarithmic scale (base 10) before the linear regression analyses.

### Phase shift analysis

We used the idea of Lissajous curve analysis^34^ to visualise phase differences between *AhgFLC* mRNA and histone modifications (H3K4me3 and H3K27me3). The observed values of *AhgFLC* mRNA and histone modifications were normalised, setting the minimum value to 0 and the maximum value to 1, each for the first year (25 September 2012–10 September 2013) and the second year (24 September 2013–16 September 2014).

### Empirical dynamic modelling

EDM is a nonlinear time series analysis used to recover the system dynamics from empirical time series without assuming any set of equations governing the system^35,36,55^. EDM is rooted in state space reconstruction (SSR), i.e. lagged coordinate embedding of time series^56^. The Takens’ theorem proves that reconstructing the system dynamics in a state space is possible by substituting the time lags of the observable variables for the unknown variables. The information in the unobserved variables is encoded in the observed time series (if they are dynamically coupled), and thus a single time series can be used to reconstruct the original state space. The number of time lags used in SSR is the number of dimensions that are necessary to resolve the original attractor (i.e. embedding dimension *E*)^55^. Before EDM, *AhgFLC* histone modifications and mRNA levels were standardised by setting the mean and standard deviation for the two-year data of each variable to 0 and 1, respectively. Air temperature was smoothened by the smooth.spline function of R setting spar, a smoothing parameter, to 0.5, because *AhgFLC* histone modification levels at most of the tested regions and the mRNA level were explained by long-term temperature trends (Fig. 2 and Supplementary Fig. 5).

CCM^35^, an EDM causality test, was used to infer the causal relationship between the histone modifications and mRNA levels of *AhgFLC*. A detailed algorithm of CCM is described elsewhere^35,57^. Briefly, CCM explores the signature of a causal variable in the time series of an effect variable by testing whether correspondence exists between their reconstructed state space. For example, if variable *X* unidirectionally affects variable *Y*, information of the causal variable *X* is encoded in the time series of the affected variable *Y*. Therefore, predicting the state of *X* is possible using the information encoded in *Y* (i.e. cross-mapping). In this study, cross-mapping from one variable to another was performed using simplex projection^37^. In the simplex projection, a set of neighbouring points of *Y* at time *t* in a reconstructed state-space are used to identify their time-corresponding points of *X*. If the time-corresponding points of the nearest neighbours of *Y* are also neighbours of *X*, then it is possible to predict *X* by using cross-mapping. Cross-map skill can be evaluated using the Pearson’s correlation coefficient (*ρ*) between predicted and observed values, and it is a proxy for causal relationships.

Time delays in causation can be considered by introducing a time lag into the cross-mapping^36^. In the case of unidirectional causality, an effect variable should predict the past values of a causal variable better than the future values because a causal variable cannot influence the past values of an effect variable^36^. By changing the time to prediction (tp) from −8 to 4 (where negative and positive tp values represent the past and future points, respectively), we determined the best tp value that showed the highest cross-map skill for each cross-mapping. For cross-mappings in which the best tp values were negative, indicating true causality, we adopted two criteria to identify the significant causal relationships for the determined tp. First, the cross-map skill for the observed data should be higher than those for its seasonal-surrogate time series (i.e. null models with the same level of seasonality) at the maximum library size (i.e. the number of points in a state space). Seasonal-surrogate time series were used to avoid the misidentification of causality owing to synchronization between the histone modifications and mRNA levels of *AhgFLC* driven by seasonality^58,59^. Second, cross-map skill should show convergence, i.e. it should be improved with an increase of library size. As the number of points in the state space increases, the trajectory defining the attractor fills in, which results in closer nearest neighbours in the state space. The convergence is an index of one-to-one correspondence between the attractors, which can be another practical criterion for causality. We set ‘(cross-map skill at final library size) – (cross-map skill at initial library size) > 0.1’ as a criterion of convergence.

The embedding dimension (*E*), i.e. how many time lags are used to reconstruct the state space, should be carefully determined because the results of CCM are sensitive to the choice of *E*. By changing the *E* value from 1 to 24, we determined the *E* value that showed the minimum values of the root mean squared error (RMSE) of the prediction by using univariate simplex projection for each variable (Supplementary Fig. 10 and 13a). The three processes, i.e. CCM, seasonal-surrogate time series generation, and the best *E* value determination, were performed using the rEDM v0.6.9 package of R using the rEDM::ccm, rEDM::make_surrogate_data, and simplex functions, respectively (10.5281/zenodo.1081784)^36^. The results were visualised using the ggplot2 package of R^60^.

### Mathematical modelling

We assumed that H3K27me3 at the *AhgFLC* locus can be classified into four states, i.e. U_N_U_D_ (the nucleation region and the distal nucleation region are not modified), M_N_U_D_ (only the nucleation region is modified), M_N_M_D_ (the nucleation region and the distal nucleation region are modified) and U_N_M_D_ (only the distal nucleation region is modified) — the proportions: *u*_N_*u*_D_, *m*_N_*u*_D_, *m*_N_*m*_D_, and *u*_N_*m*_D_, respectively (Fig. 5a). Based on the observed dynamics of H3K27me3 at the *AhgFLC* locus (Fig. 1e, f), we assumed the transition of H3K27me3 at the locus to be unidirectional, i.e. U_N_U_D_ → M_N_U_D_ → M_N_M_D_ → U_N_M_D_ → U_N_U_D_ (Fig. 5a). We described the H3K4me3 states by U_N_ and A_N_ for the nucleation region (the proportions: *u*_N_ and *a*_N_), and U_D_ and A_D_ for the distal nucleation region (the proportions: *u*_D_ and *a*_D_; Fig. 5a). To link the tissue-level observations in experiments with the locus-level H3K27me3 and H3K4me3 states, we derived differential equation models from stochastic models that assumes the *AhgFLC* regulation in cis (Supplementary Note 1). We confirmed that the results were similar between the original stochastic model and the differential equation model (Supplementary Fig. 15). We explain the derived differential equation models below.

Based on previous reports of vernalisation processes in *A. thaliana*^13,14,22,23^, we assumed that the transitions U_N_U_D_ → M_N_U_D_ and M_N_U_D_ → M_N_M_D_ are induced by cold and warm temperatures, represented by the functions of temperature, *μ*(*T*) and *ν*(*T*), respectively. These functions are given by the logistic equations of the form

1 $$\mu \left( T \right) = \frac{\zeta }{{1 + \exp \left( {\alpha \left( {T - \theta _1} \right)} \right)}}$$

2 $$\nu \left( T \right) = \frac{\eta }{{1 + \exp \left( { - \beta \left( {T - \theta _2} \right)} \right)}}$$

Based on the results of EDM (Fig. 4 and Supplementary Figs. 11, 12), we assumed that the M_N_M_D_ → U_N_M_D_ transition is induced by H3K4me3 at the nucleation region (*a*_N_), and that the U_N_M_D_ → U_N_U_D_ transition occurs at a constant rate (*λ*). Thus, the time derivatives of the H3K27me3 states at the *AhgFLC* locus are given by

3 $$\frac{{du_{\mathrm{N}}u_{\mathrm{D}}}}{{dt}} = \lambda \,u_{\mathrm{N}}m_{\mathrm{D}} - \mu \left( T \right)u_{\mathrm{N}}u_{\mathrm{D}}$$

4 $$\frac{{dm_{\mathrm{N}}u_{\mathrm{D}}}}{{dt}} = \mu \left( T \right)\,u_{\mathrm{N}}u_{\mathrm{D}} - \nu \left( T \right)m_{\mathrm{N}}u_{\mathrm{D}}$$

5 $$\frac{{dm_{\mathrm{N}}m_{\mathrm{D}}}}{{dt}} = \nu \left( T \right)\,m_{\mathrm{N}}u_{\mathrm{D}} - \kappa \,a_{\mathrm{N}}\,m_{\mathrm{N}}m_{\mathrm{D}}$$

6 $$\frac{{du_{\mathrm{N}}m_{\mathrm{D}}}}{{dt}} = \kappa \,a_{\mathrm{N}}\,m_{\mathrm{N}}m_{\mathrm{D}} - \lambda \,u_{\mathrm{N}}m_{\mathrm{D}}$$

The observed H3K27me3 levels at the nucleation region and the distal nucleation region were represented by the mean levels of amplicons I and II, and VI–VIII, respectively. Before the mathematical model was optimised, the summed proportion of M_N_U_D_ and M_N_M_D_ (*m*_N_*u*_D_ + *m*_N_*m*_D_) and that of M_N_M_D_ and U_N_M_D_ (*m*_N_*m*_D_ + *u*_N_*m*_D_) were multiplied by the two-year maximum of the H3K27me3 levels observed at the nucleation region and the distal nucleation region, respectively, to represent the simulated H3K27me3 levels at these regions.

Based on the seasonal dynamics, we assumed that H3K4me3 at the nucleation region and the distal nucleation region accumulates in response to warm [*ξ*(*T*)] and cold [*τ*(*T*)] temperatures, respectively. These functions are given by the logistic equations of the form

7 $$\xi \left( T \right) = \frac{\iota }{{1 + \exp \left( { - \gamma \left( {T - \theta _3} \right)} \right)}}$$

8 $$\tau \left( T \right) = \frac{\rho }{{1 + \exp \left( {\varepsilon \left( {T - \theta _4} \right)} \right)}}$$

Based on the results of EDM (Fig. 4 and Supplementary Figs. 11, 12), we assumed that H3K4me3 at the distal nucleation region negatively affects that at the nucleation region, and vice versa. We also assumed that H3K27me3 negatively affects H3K4me3 at the two nucleation regions. Thus, the time derivatives of the H3K4me3 states at the *AhgFLC* locus are given by

9 $$\frac{{da_{\mathrm{N}}}}{{dt}} = \xi \left( T \right)(1 - a_{\mathrm{D}})u_{\mathrm{N}} - \varphi \left( {m_{\mathrm{N}}u_{\mathrm{D}} + m_{\mathrm{N}}m_{\mathrm{D}}} \right)a_{\mathrm{N}}$$

10 $$\frac{{da_{\mathrm{D}}}}{{dt}} = \tau \left( T \right)\left( {1 - a_{\mathrm{N}}} \right)u_{\mathrm{D}} - \psi \left( {m_{\mathrm{N}}m_{\mathrm{D}} + u_{\mathrm{N}}m_{\mathrm{D}}} \right)a_{\mathrm{D}}$$

The observed H3K4me3 levels at the nucleation region and the distal nucleation region were represented by the mean levels of amplicons I and II, and VI–VIII, respectively. Before the mathematical model was optimised, the proportion of A_N_ (*a*_N_) and A_D_ (*a*_D_) were multiplied by the two-year maximum of the H3K4me3 levels observed at the nucleation region and the distal nucleation region, respectively, to represent the simulated H3K4me3 levels at these regions.

The simulation was performed by the ode function of the deSolve package of R. The parameters in the model were optimised by fitting the simulated H3K27me3 and H3K4me3 levels to the observed values. Fitting was conducted by minimising the residual sum of squares between the model output and the observed data in two steps. First, 1,000 parameter sets were randomly chosen from the uniform distribution, and the best parameter set which fitted the observed data was selected. Using the parameter set as the initial values, we then optimised the parameters by using the simulated annealing (SANN) method^61^ using the optim function of R. The first values of the observed data (25 September 2012) were used as the initial state of the H3K27me3 and H3K4me3 levels in the simulation. Explanations and the determined values of the parameters are listed in Supplementary Table 2. For the simulation, we used air temperature data recorded every 10 min at the meteorological station nearest to our field site (Nishiwaki, Hyogo; Supplementary Fig. 3a). The temperature data was interpolated by the approxfun function of R before the simulation.

In the optimisation of the parameters, we set the upper limits of all parameters to 100 to avoid divergence of the values. Based on the comparison between air temperature data (Supplementary Fig. 3a) and the seasonal dynamics of *AhgFLC* H3K27me3 and H3K4me3 at the nucleation region and the distal nucleation region (Fig. 1c–f), we set the threshold temperatures *θ*_1_−*θ*_4_ to 5, 10, 15 and 5 °C, respectively.

The observed *AhgFLC* mRNA level was modelled via linear regression with the observed H3K4me3 level at the nucleation region (Fig. 5a). The equation is given by

11 $${\mathrm{log}}_{10}\left( {RNA} \right) = \sigma \,{\mathrm{log}}_{10}\left( K4 \ at \ NR \right) + \omega$$

Using the determined regression coefficient and intercept, *AhgFLC* mRNA level in each model was reproduced from the simulated H3K4me3 level at the nucleation region. The mRNA level was standardised to make the seasonal amplitude even between models.

### Transplant experiments

We conducted transplant experiments on 26 March, 10 April, 24 April and 3 July in 2018 during seasonal increases in temperature (Supplementary Fig. 16a). On each date, six plants from the natural population were transferred to a cold growth chamber [4.5 °C ± 0.5 °C (SD) 12 h light/12 h dark cycles]. On 26 March, we transferred additional six plants to a warm growth chamber [23.3 °C ± 0.9 °C (SD) 12 h light/12 h dark cycles]. For RNA samples, we harvested one small young leaf (~0.01 g) from each plant at six different times (the sampling schedule is shown in Supplementary Fig. 16b, c) and obtained three biological replicates by pooling two leaves on each sampling date. The harvested leaves were preserved in RNA*later* Stabilization Solution (Thermo Fisher Scientific) on ice, kept at 4 °C for one day and then stored at −20 °C. For ChIP samples, we harvested one fully expanded young leaf (~0.1 g) from each plant at four different times (the sampling schedule is shown in Supplementary Fig. 16b, c) and obtained three biological replicates by pooling two leaves on each sampling date. Samples were fixed with 1% formaldehyde in PBS in the field on the first sampling date and later in the laboratory.

### Statistical analysis

All the descriptions of statistical analyses are provided in the figure legends and Methods section.

### Reporting summary

Further information on research design is available in the Nature Research Reporting Summary linked to this article.

## Supplementary information

Supplementary Information

Peer Review

Reporting summary

## Data Availability

Data supporting the findings of this work are available within the paper and its Supplementary Information file. A reporting summary for this Article is available as a Supplementary Information file. The data sets generated and analysed during the current study are available from the corresponding author upon request. The source data underlying Figs. 1b–g, 2a–d, 3–5a–d, 6a and c–h, as well as Supplementary Figs. 2b, 3c–g and 7 are provided as a Source Data file.

## Source Data

Source Data

## Code availability

The R codes used in this study are available at https://github.com/hnishio/Nishio_Nat.Commun_script.
