# Supplementary material for: Repressive chromatin modification underpins the long-term expression trend of a perennial flowering gene in nature
Source: Nat Commun. 2020 May 1;11:2065. doi: 10.1038/s41467-020-15896-4 (PMC7195410; doi:10.1038/s41467-020-15896-4)
Supplement: Supplementary file 1 — Supplementary Information [file 41467_2020_15896_MOESM1_ESM.pdf]

Supplementary Information for

Repressive chromatin modification underpins the long-term expression trend of a perennial flowering gene in nature

Nishio et al.

a

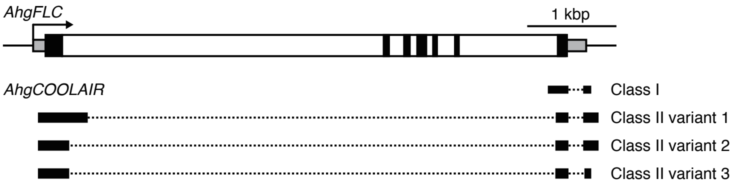

b

> *AhgCOOLAIR* Class I
GGTTGTTAGTCTTAATTGATGGTTTGAAATAGATATTCCTTGGATAGAAGACAAAAAGTAGAAAGTGAATAGTGATTTTGACCTATGATTATC
GTACAGATGGAGAAGAATCATCATGTGGGAGCAGAAGCTGAGATGGAGATTTCACCTGCAGGACAAATCTCCGACAATCTTCCGGTGACTCTC
CCGCTGCTTAATTAGCCACC *INTRON* TTATTCGCGAGAGAATTGCGTCGAGATCTTGTTTGTGTTCTTCTCT**TCTGTCCAAAACTTGTGT
TTGC**

> *AhgCOOLAIR* Class II variant 1
GTATCTCCGGCGACTTGAACCCAACCTGAGGATCAAACTAGGGAATAAAGCCCTCTCGGAGACAGAAGCCATGGGGAGAAAAAAACTAGAAAT
CAAGCGAATTGAGAACAAAAGTAGCCGACAAGTCACCTTCTCCAAACGTCGCAACGGTCTCATCGAGAAAGCTCGTCAGCTTTCTGTTCTCTG
TGACGCATCCGTCGCTCTTCTCGTCGTCTCTGCTTCCGGCAAGCTCTACAGCTTCTCCTCCGGCGATAAGTACGCCTCTTCCTTATCTGGGTT
TCCATTTATTTCCCCTTTAATCTTCAGTTTTGTGCTCTTTATTTTTCTGCAAAAATAAAAATAACAAACAATTAATAAACCGCTTGATTTCTT
TCCGGCGAATCTCTTGTTGTTTCCGTTGACACACAAAAAAAAAAAATAAAAAAAAAAATAAAATAAAAGAATCTCTTGTTGTTTCCCGGTTCGG
TGTTTTTAGTGTTTTTTCCTATGAGGGGCGGAGGTTGTAAAGCATCATGGGGTCATATGACACCTAT *INTRON* GATTATCGTACAGATGG
AGAAGAATCATCATGTGGGAGCAGAAGCTGAGATGGAGATTTCACCTGCAGGACAAATCTCCGACAATCTTCCGGTGACTCTCCCGCTGCTTA
ATTAGCCACC *INTRON* TTATTCGCGAGAGAATTGCGTCGAGATCTTGTTTGTGTTCTTCTCTTCTGTCCAAAACTTGTGTTTGCTTCACT
GTGAAGAAGCCTACGGCTTATTTATTTTTTTGGTAGANGAAGCCTACGGCTTATTG**TGCAAAGGAAACGTGGCTCT**

> *AhgCOOLAIR* Class II variant 2
GTATCTCCGGCGACTTGAACCCAACCTGAGGATCAAACTAGGGAATAAAGCCCTCTCGGAGACAGAAGCCATGGGGAGAAAAAAACTAGAAAT
CAAGCGAATTGAGAACAAAAGTAGCCGACAAGTCACCTTCTCCAAACGTCGCAACGGTCTCATCGAGAAAGCTCGTCAGCTTTCTGTTCTCTG
TGACGCATCCGTCGCTCTTCTCGTCGTCTCTGCTTCCGGCAAGCTCTACAGCTTCTCCTCCGGCGATAAGTACGCCTCTTCCTTATCTGGGTT
TCCATTTATTTCCCCTTTAATCTTCAGTTTTGTGCTCTTTATTTTT *INTRON* CTATGATTATCGTACAGATGGAGAAGAATCATCATGTG
GGAGCAGAAGCTGAGATGGAGATTTCACCTGCAGGACAAATCTCCGACAATCTTCCGGTGACTCTCCCGCTGCTTAATTAGCCACC *INTRON*
TTATTCGCGAGAGAATTGCGTCGAGATCTTGTTTGTGTTCTTCTCTTCTGTCCAAAACTTGTGTTTGCTTCACTGTGAAGAAGCCTACGGCT
TATTTATTTTTTTGGTAGAAGAAGCCTACGGCTTATTG**TGCAAAGGAAACGTGGCTCT**

> *AhgCOOLAIR* Class II variant 3
GTATCTCCGGCGACTTGAACCCAACCTGAGGATCAAACTAGGGAATAAAGCCCTCTCGGAGACAGAAGCCATGGGGAGAAAAAAACTAGAAAT
CAAGCGAATTGAGAACAAAAGTAGCCGACAAGTCACCTTCTCCAAACGTCGCAACGGTCTCATCGAGAAAGCTCGTCAGCTTTCTGTTCTCTG
TGACGCATCCGTCGCTCTTCTCGTCGTCTCTGCTTCCGGCAAGCTCTACAGCTTCTCCTCCGGCGATAAGTACGCCTCTTCCTTATCTGGGTT
TCCATTTATTTCCCCTTTAATCTTCAGTTTTGTGCTCTTTATTTTT *INTRON* CTATGATTATCGTACAGATGGAGAAGAATCATCATGTG
GGAGCAGAAGCTGAGATGGAGATTTCACCTGCAGGACAAATCTCCGACAATCTTCCGGTGACTCTCCCGCTGCTTAATTAGCCACCT *INTRO
N* ATTGCGTCGAGATCTTGTTTGTGTTCTTCTCT**TCTGTCCAAAACTTGTGTTTGC**

Supplementary Fig. 1 Partial structure of *AhgCOOLAIR* variants. **a** Structure of the *AhgFLC* locus (top: grey, untranslated regions; black, exons; white, introns) and partial *AhgCOOLAIR* variants (bottom: filled boxes, exons; dotted lines, introns). **b** Sequences of *AhgCOOLAIR* in the sense orientation of *AhgFLC* mRNA. Bold underlined letters represent the forward and reverse primers used for PCR. The locations of introns are shown.

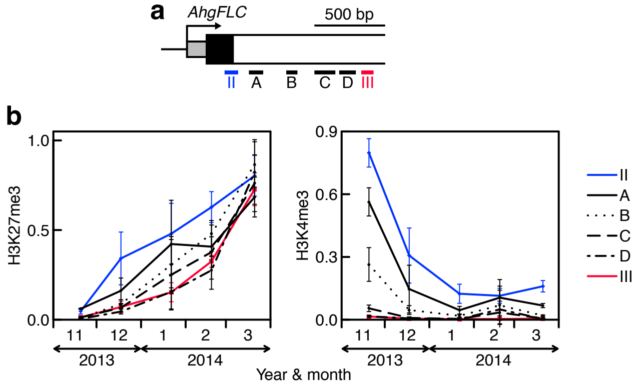

Supplementary Fig. 2 Seasonal dynamics of *AhgFLC* H3K27me3 and H3K4me3 levels at approximately 1 kbp region between amplicons II and III in the natural habitat. **a** Distribution of four additional ChIP amplicons (A–D) set between amplicons II and III. **b** Monthly quantification of *AhgFLC* H3K27me3 and H3K4me3 at amplicons II, III, and A–D in the natural population of *A. halleri* from November 2013 to March 2014. Data are represented relative to *AhgSTM* (H3K27me3) and *AhgACT2* (H3K4me3). The means and standard deviations of biological replicates are shown. $n = 3$ for H3K27me3 at amplicon A on 19 Nov. 2013, and H3K4me3 at amplicon A on 19 Nov. 2013 and at amplicon C on 17 Dec. 2013. $n = 4$ for the other data points. For each replicate, pool of leaves from ten plants (out of 40 plants) was analysed. Source data underlying Supplementary Figure 2b are provided as a Source Data file.

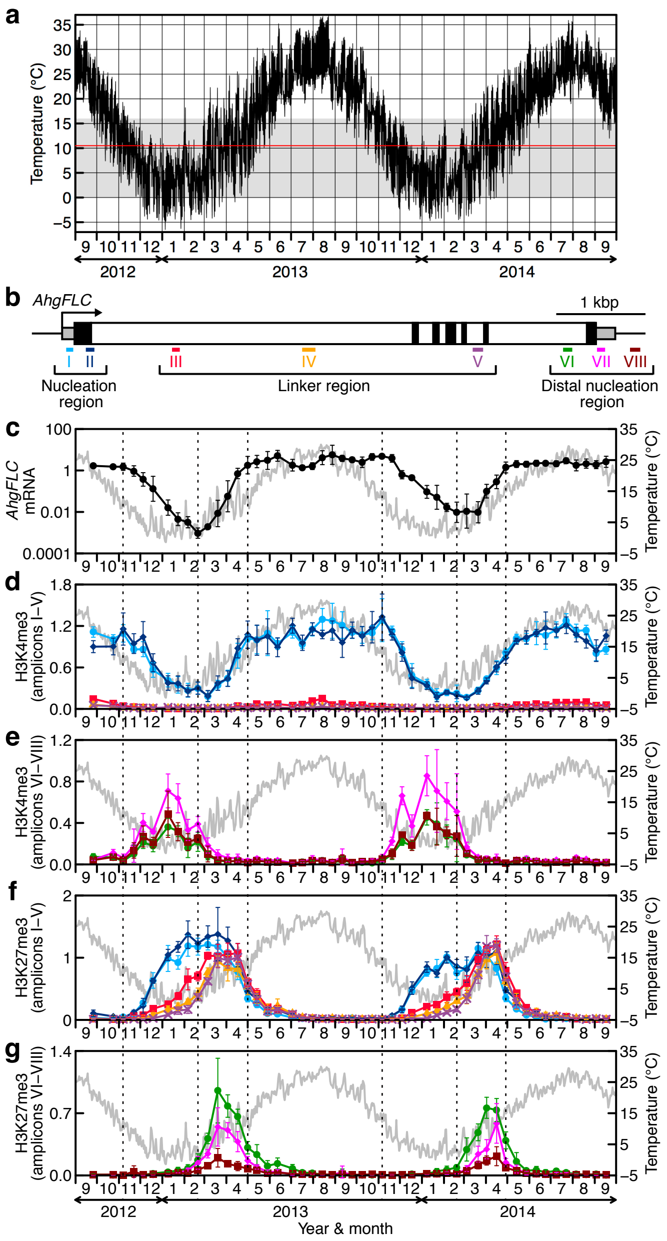

Supplementary Fig. 3 Seasonal dynamics of *AhgFLC* mRNA and histone modification levels for two years in the natural habitat, normalised by alternative reference genes. **a** Air temperature recorded every 10 min at the meteorological station nearest to our field site (Nishiwaki, Hyogo, Japan). A red line represents the vernalisation threshold in *A. halleri* (10.5°C) and a shaded region represents the vernalisation-effective range in *A. thaliana* (0−16°C). **b** Structure of the *AhgFLC* locus with untranslated regions (grey), exons (black), and introns (white); distribution of eight H3K4me3 and H3K27me3 ChIP amplicons in different colours and the definitions of the nucleation region, linker region, and distal nucleation region. **c**−**g** Relative quantification of the seasonal dynamics of *AhgFLC* mRNA (**c**), H3K4me3 at amplicons I−V (**d**), H3K4me3 at amplicons VI−VIII (**e**), H3K27me3 at amplicons I−V (**f**), and H3K27me3 at amplicons VI−VIII (**g**) in the natural population of *A. halleri* at two-week intervals. The daily means of air temperature are plotted in grey. The colour code in **d**−**g** corresponds to that in **b**. The qPCR data of *AhgFLC* are represented relative to those of *AhgPP2AA3* (mRNA and H3K4me3) and *AhgFUS3* (H3K27me3). The means and standard deviations of biological replicates are shown. n = 4 for mRNA, H3K4me3 and H3K27me3 at amplicons I−V. n = 3−4 (average, >3.9) for H3K4me3 and H3K27me3 at amplicons VI−VIII. For each replicate, a pool of leaves from ten plants (out of 40 plants) was analysed. Source data underlying Supplementary Figure 3c–g are provided as a Source Data file.

a

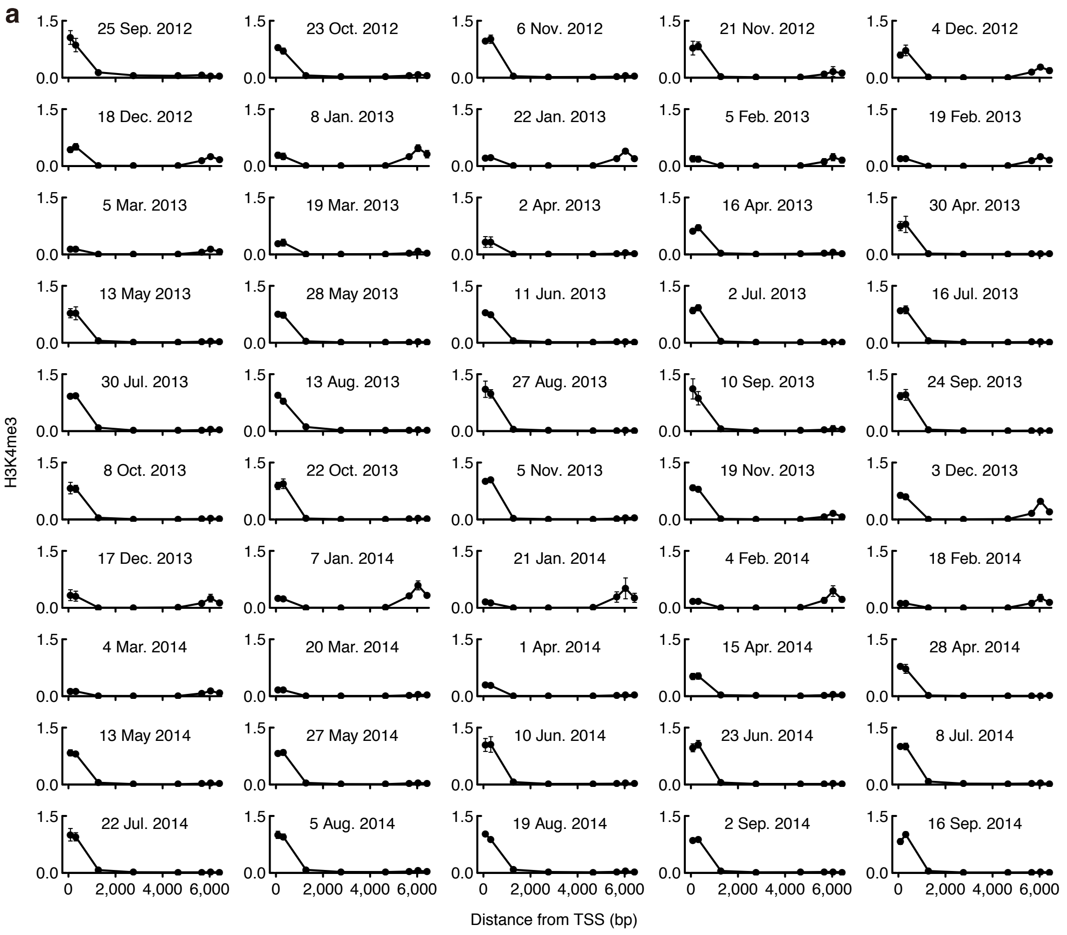
H3K4me3
Distance from TSS (bp)
25 Sep. 2012
23 Oct. 2012
6 Nov. 2012
21 Nov. 2012
4 Dec. 2012
18 Dec. 2012
8 Jan. 2013
22 Jan. 2013
5 Feb. 2013
19 Feb. 2013
5 Mar. 2013
19 Mar. 2013
2 Apr. 2013
16 Apr. 2013
30 Apr. 2013
13 May 2013
28 May 2013
11 Jun. 2013
2 Jul. 2013
16 Jul. 2013
30 Jul. 2013
13 Aug. 2013
27 Aug. 2013
10 Sep. 2013
24 Sep. 2013
8 Oct. 2013
22 Oct. 2013
5 Nov. 2013
19 Nov. 2013
3 Dec. 2013
17 Dec. 2013
7 Jan. 2014
21 Jan. 2014
4 Feb. 2014
18 Feb. 2014
4 Mar. 2014
20 Mar. 2014
1 Apr. 2014
15 Apr. 2014
28 Apr. 2014
13 May 2014
27 May 2014
10 Jun. 2014
23 Jun. 2014
8 Jul. 2014
22 Jul. 2014
5 Aug. 2014
19 Aug. 2014
2 Sep. 2014
16 Sep. 2014
1.5
0.0
0
2,000
4,000
6,000

b

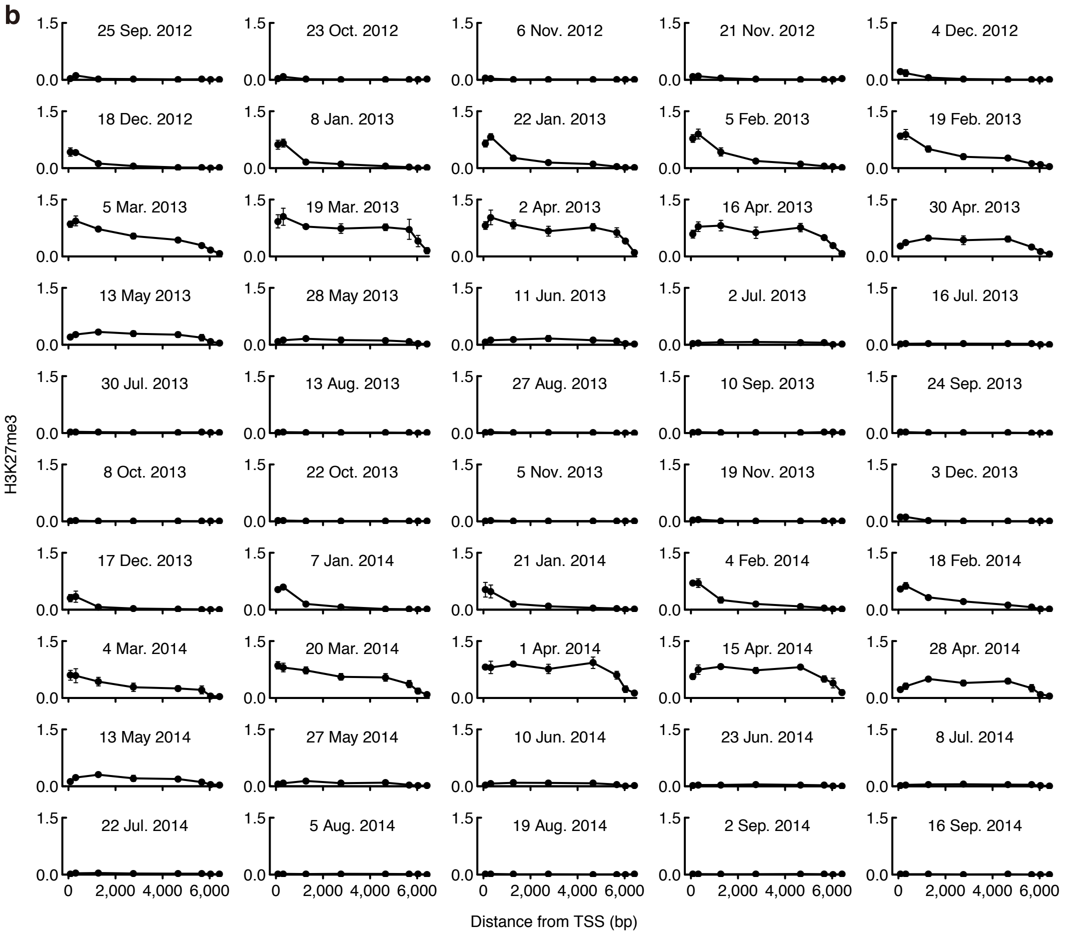
H3K27me3
Distance from TSS (bp)
25 Sep. 2012
23 Oct. 2012
6 Nov. 2012
21 Nov. 2012
4 Dec. 2012
18 Dec. 2012
8 Jan. 2013
22 Jan. 2013
5 Feb. 2013
19 Feb. 2013
5 Mar. 2013
19 Mar. 2013
2 Apr. 2013
16 Apr. 2013
30 Apr. 2013
13 May 2013
28 May 2013
11 Jun. 2013
2 Jul. 2013
16 Jul. 2013
30 Jul. 2013
13 Aug. 2013
27 Aug. 2013
10 Sep. 2013
24 Sep. 2013
8 Oct. 2013
22 Oct. 2013
5 Nov. 2013
19 Nov. 2013
3 Dec. 2013
17 Dec. 2013
7 Jan. 2014
21 Jan. 2014
4 Feb. 2014
18 Feb. 2014
4 Mar. 2014
20 Mar. 2014
1 Apr. 2014
15 Apr. 2014
28 Apr. 2014
13 May 2014
27 May 2014
10 Jun. 2014
23 Jun. 2014
8 Jul. 2014
22 Jul. 2014
5 Aug. 2014
19 Aug. 2014
2 Sep. 2014
16 Sep. 2014
1.5
0.0
0
2,000
4,000
6,000

Supplementary Fig. 4 H3K4me3 and H3K27me3 levels along the *AhgFLC* locus for the two-year measurements in the natural population. **a**, **b** H3K4me3 (**a**) and H3K27me3 (**b**) levels are shown against distance from TSS along the *AhgFLC* locus. Data are represented relative to *AhgACT2* (H3K4me3) and *AhgSTM* (H3K27me3). The means and standard deviations of biological replicates are shown. $n = 3$ for H3K27me3 at amplicons VI−VIII on 18 Dec. 2012 and 19 Mar. 2013, and at amplicon VII on 19 Nov. 2013 and 21 Jan. 2014, and H3K4me3 at amplicons VI−VIII on 4 Dec. 2012 and 18 Dec. 2012, at amplicon VIII on 11 Jun. 2013, and at amplicon VII on19 Nov. 2013. $n = 4$ for the other data points. For each replicate, pool of leaves from ten plants (out of 40 plants) was analysed.

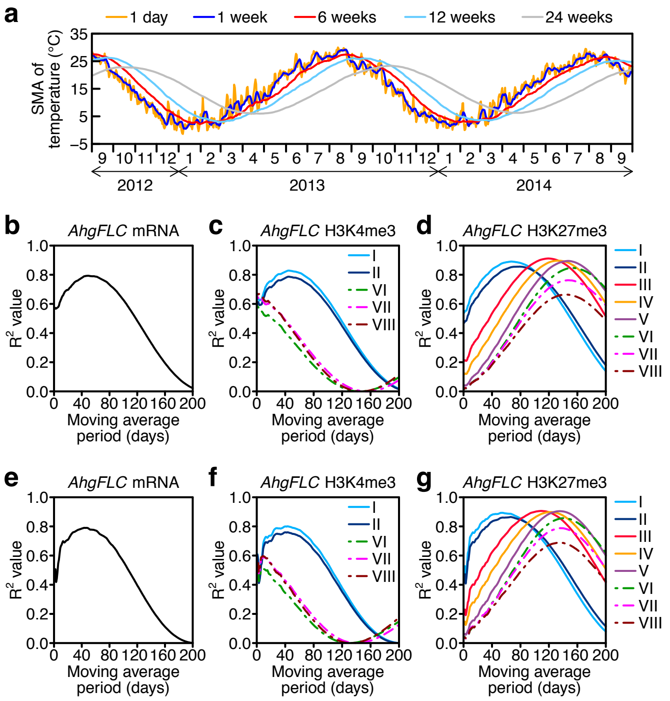

Supplementary Fig. 5 Linear regression of mRNA and histone modification levels on the simple moving averages (SMAs) of past temperature with different window lengths. **a** The SMAs of past temperature with window lengths of 1 day, and 1, 6, 12, and 24 weeks for the study period of two years. **b**−**d** The results of linear regression analyses on the SMAs of the daily maximum temperature with different window lengths. R^2 values for *AhgFLC* mRNA (**b**), H3K4me3 (**c**) and H3K27me3 (**d**) levels are shown. **e**−**g** The results of linear regression analyses on the SMAs of the daily minimum temperature with different window lengths. Data are normalised against *AhgACT2* (mRNA and H3K4me3) and *AhgSTM* (H3K27me3) before regression analyses.

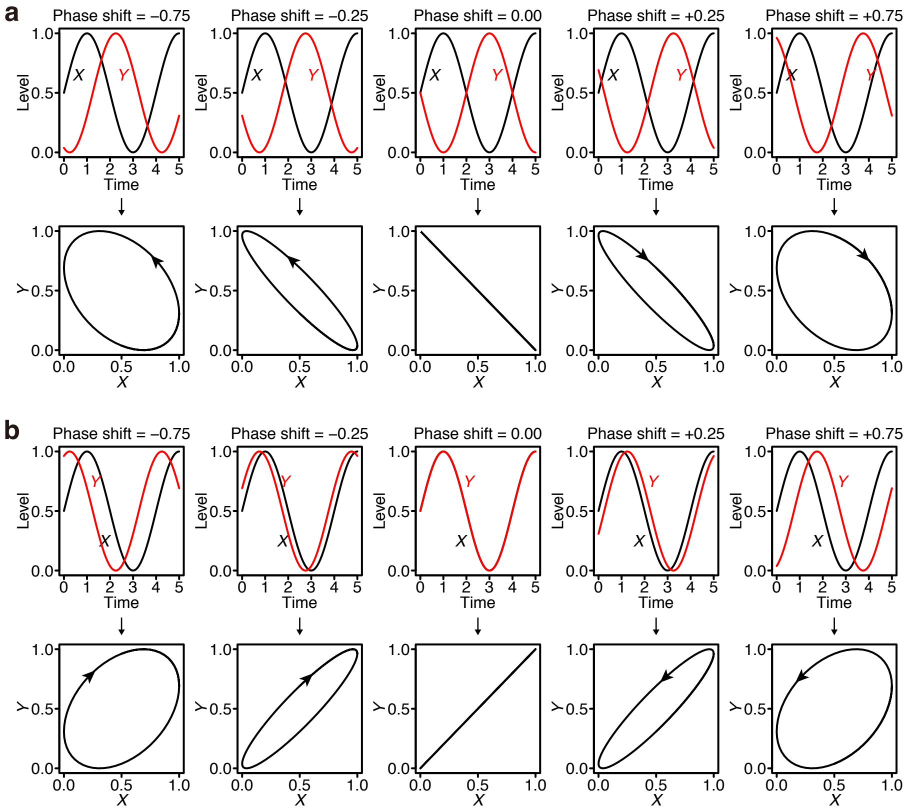

Supplementary Fig. 6 Conceptual diagrams showing the relationship between the degree of phase difference and Lissajous curves. For simplicity, the two variables (*X* and *Y*) were assumed to follow sine curves. When the phase shift is 0, the Lissajous curve shows a linear trajectory and becomes ellipse according to the advance or delay in the phase of *Y*. **a** The comparison between *X* and *Y*, in which 'anti-phase' state was defined to have no phase shift (phase shift = 0.00). When *Y* delays from *X*, it shows anticlockwise rotation; when *Y* precedes *X*, it shows clockwise rotation. **b** The comparison of *X* and *Y*, in which 'in-phase' state was defined to have no phase shift (phase shift = 0.00). When Y delays from *X*, the Lissajous curve shows clockwise rotation; when *Y* precedes *X*, it shows anticlockwise rotation.

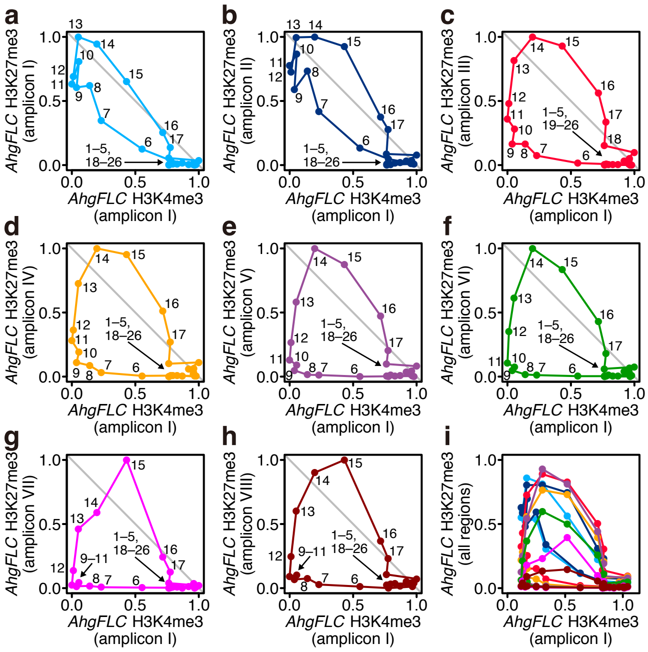

Supplementary Fig. 7 Phase differences between the seasonal dynamics of *AhgFLC* H3K27me3 and H3K4me3 for the second year measurement. **a**–**h** Lissajous curves, i.e. time-series trajectories of two sets of cyclical values, delineated by plotting *AhgFLC* H3K4me3 at amplicon I on the horizontal axis and H3K27me3 at amplicon I (**a**), amplicon II (**b**), amplicon III (**c**), amplicon IV (**d**), amplicon V (**e**), amplicon VI (**f**), amplicon VII (**g**) and amplicon VIII (**h**) on the vertical axis. *AhgFLC* H3K27me3 and H3K4me3 levels are shown as relative values, setting the minimum level to 0 and the maximum level to 1 in **a**–**h**. The numbers next to the data points are chronological ordinals (1: 24 September 2013, 26: 16 September 2014). **i** Lissajous curves drawn using the absolute values for all tested regions. The colour code corresponds to that in Fig. 1a. Data are normalised against *AhgSTM* (H3K27me3) and *AhgACT2* (H3K4me3) and shown as the means of four biological replicates. Source data are provided as a Source Data file.

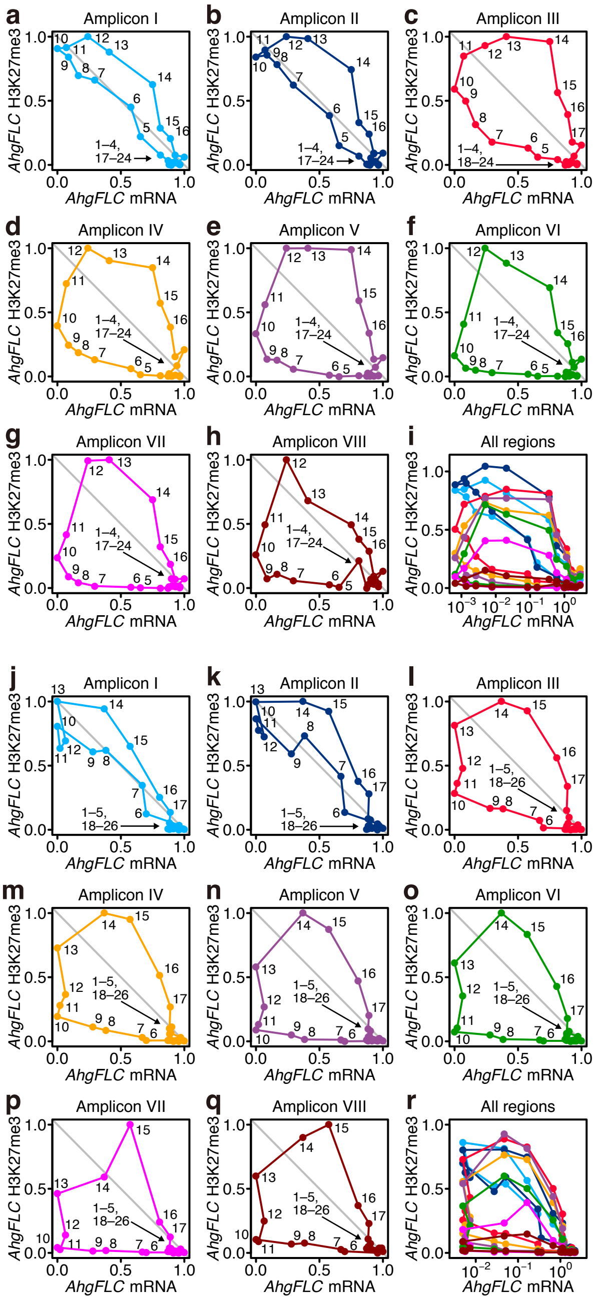

Supplementary Fig. 8 Phase differences between the seasonal dynamics of *AhgFLC* H3K27me3 and mRNA. **a**–**h** Lissajous curves, i.e. time-series trajectories of two sets of cyclical values, delineated by plotting *AhgFLC* mRNA on the horizontal axis and H3K27me3 at amplicon I (**a**), amplicon II (**b**), amplicon III (**c**), amplicon IV (**d**), amplicon V (**e**), amplicon VI (**f**), amplicon VII (**g**) and amplicon VIII (**h**) on the vertical axis for the measurement conducted during the first year. *AhgFLC* H3K27me3 and mRNA levels are shown as relative values, setting the minimum level to 0 and the maximum level to 1 in **a**–**h**. The numbers next to the data points are chronological ordinals (1: 25 September 2012, 24: 10 September 2013). **i** Lissajous curves drawn using the absolute values for all tested regions. **j**–**r** Lissajous curves between *AhgFLC* H3K27me3 and mRNA for the measurement conducted during the second year (1: 24 September 2013, 26: 16 September 2014). Otherwise the same as **a**–**i**. The colour code corresponds to that in Fig. 1a. Data are normalised against *AhgSTM* (H3K27me3) and *AhgACT2* (mRNA) and shown as the means of four biological replicates.

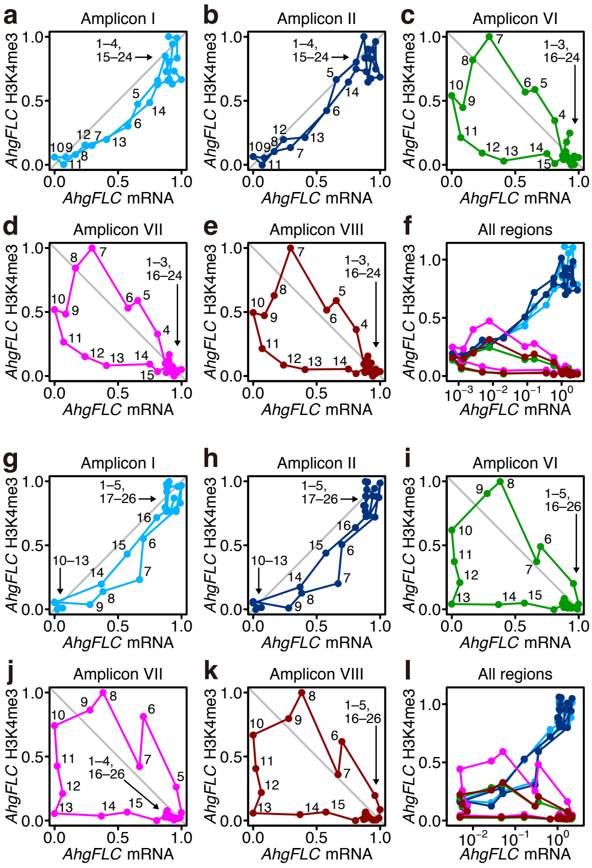

Supplementary Fig. 9 Phase differences between the seasonal dynamics of *AhgFLC* H3K4me3 and mRNA. **a**–**e** Lissajous curves, i.e., time-series trajectories of two sets of cyclical values, delineated by plotting *AhgFLC* mRNA on the horizontal axis and H3K4me3 at amplicon I (**a**), amplicon II (**b**), amplicon VI (**c**), amplicon VII (**d**) and amplicon VIII (**e**) on the vertical axis for the measurement conducted during the first year. *AhgFLC* H3K4me3 and mRNA levels are shown as relative values, setting the minimum level to 0 and the maximum level to 1 in **a**–**e**. The numbers next to the data points are chronological ordinals (1: 25 September 2012, 24: 10 September 2013). **f** Lissajous curves drawn using absolute values for all tested regions. **g**–**l** Lissajous curves between *AhgFLC* H3K4me3 and mRNA for the measurement conducted during the second year (1: 24 September 2013, 26: 16 September 2014). Otherwise the same as **a**–**f**. The colour code corresponds to that in Fig. 1a. Data are normalised against *AhgACT2* (H3K4me3 and mRNA) and shown as the means of four biological replicates.

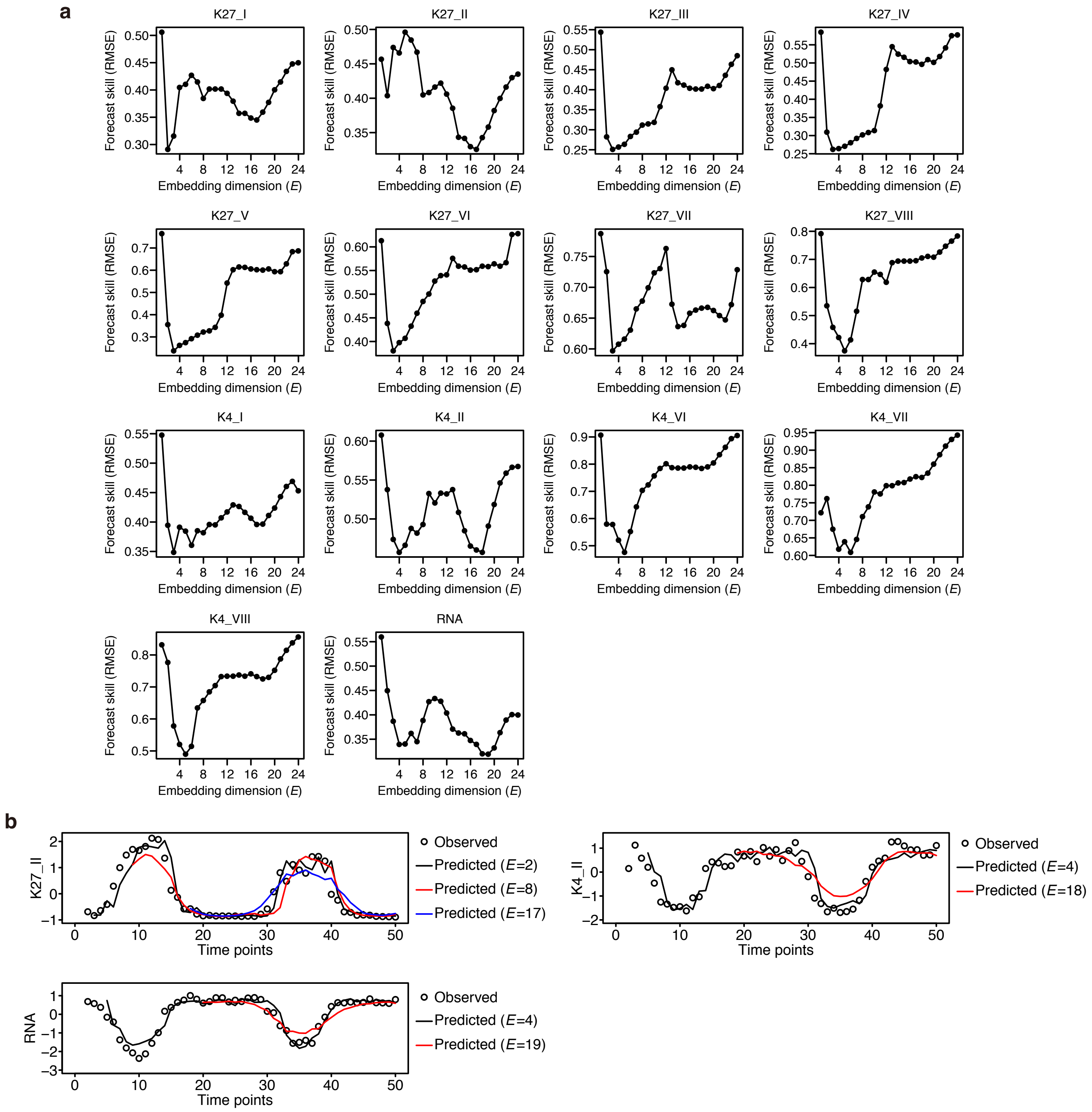

Supplementary Fig. 10 Determination of embedding dimension (*E*) for *AhgFLC* histone modification and mRNA levels. **a** By changing *E* value from 1 to 24, we determined the best *E* value showing the minimum root mean squared error (RMSE) of prediction by using univariate simplex projection. **b** For K27_II, K4_II, and RNA, observed and predicted dynamics were compared, because they showed considerably larger best *E* values than others. Predicted K27_II, K4_II, and RNA dynamics, respectively, with *E* = 2, 4, and 4 fitted the observed values well. The optimal *E* values were, K27_I: 2, K27_II: 2, K27_III: 3, K27_IV: 3, K27_V: 3, K27_VI: 3, K27_VII: 3, K27_VIII: 5, K4_I: 3, K4_II: 4, K4_VI: 5, K4_VII: 6, K4_VIII: 5, and RNA: 4.

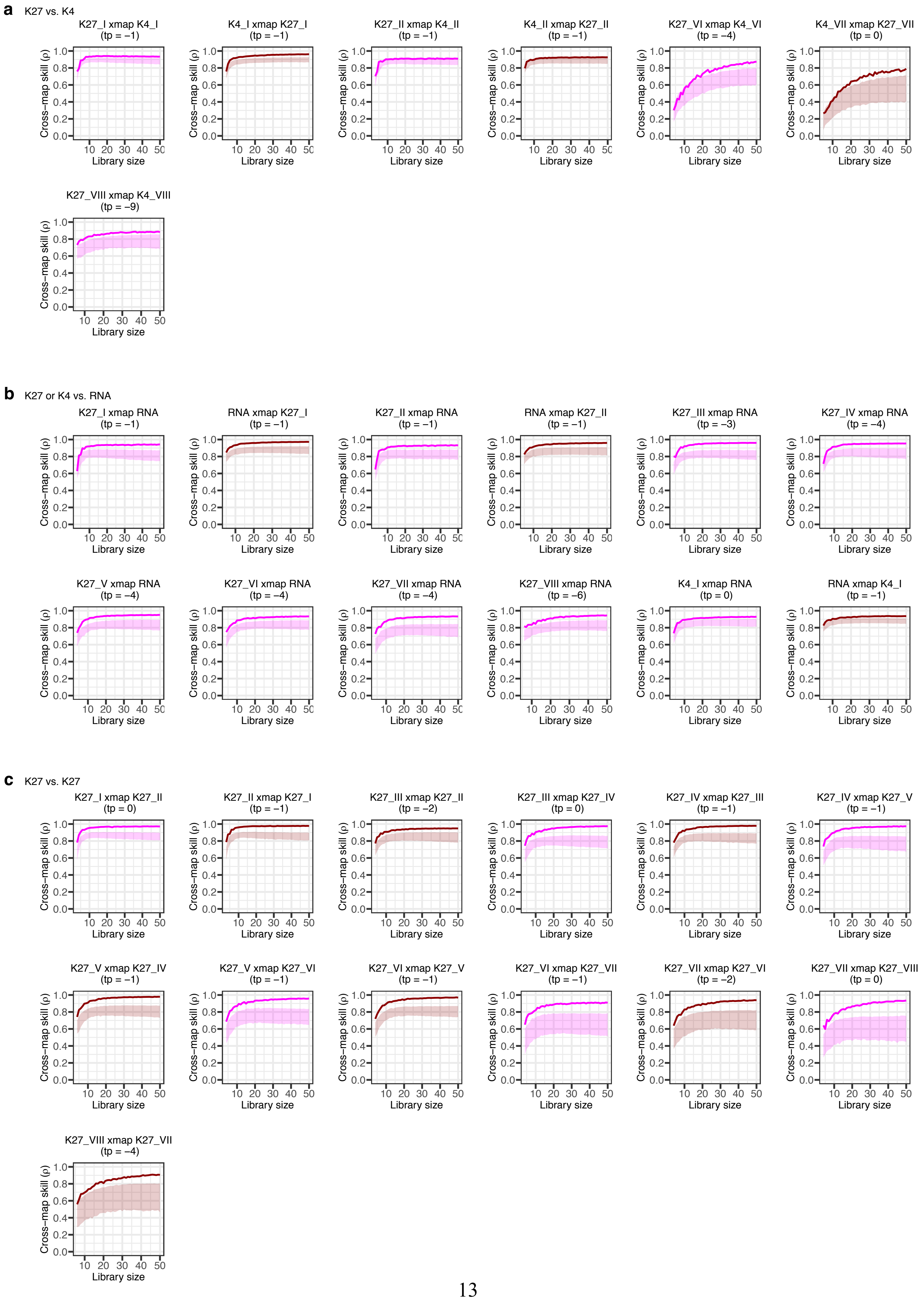
a K27 vs. K4
K27_I xmap K4_I (tp = −1)
K4_I xmap K27_I (tp = −1)
K27_II xmap K4_II (tp = −1)
K4_II xmap K27_II (tp = −1)
K27_VI xmap K4_VI (tp = −4)
K4_VII xmap K27_VII (tp = 0)
K27_VIII xmap K4_VIII (tp = −9)
b K27 or K4 vs. RNA
K27_I xmap RNA (tp = −1)
RNA xmap K27_I (tp = −1)
K27_II xmap RNA (tp = −1)
RNA xmap K27_II (tp = −1)
K27_III xmap RNA (tp = −3)
K27_IV xmap RNA (tp = −4)
K27_V xmap RNA (tp = −4)
K27_VI xmap RNA (tp = −4)
K27_VII xmap RNA (tp = −4)
K27_VIII xmap RNA (tp = −6)
K4_I xmap RNA (tp = 0)
RNA xmap K4_I (tp = −1)
c K27 vs. K27
K27_I xmap K27_II (tp = 0)
K27_II xmap K27_I (tp = −1)
K27_III xmap K27_II (tp = −2)
K27_III xmap K27_IV (tp = 0)
K27_IV xmap K27_III (tp = −1)
K27_IV xmap K27_V (tp = −1)
K27_V xmap K27_IV (tp = −1)
K27_V xmap K27_VI (tp = −1)
K27_VI xmap K27_V (tp = −1)
K27_VI xmap K27_VII (tp = −1)
K27_VII xmap K27_VI (tp = −2)
K27_VII xmap K27_VIII (tp = 0)
K27_VIII xmap K27_VII (tp = −4)
Cross−map skill (ρ)
Library size

d K4 vs. K4

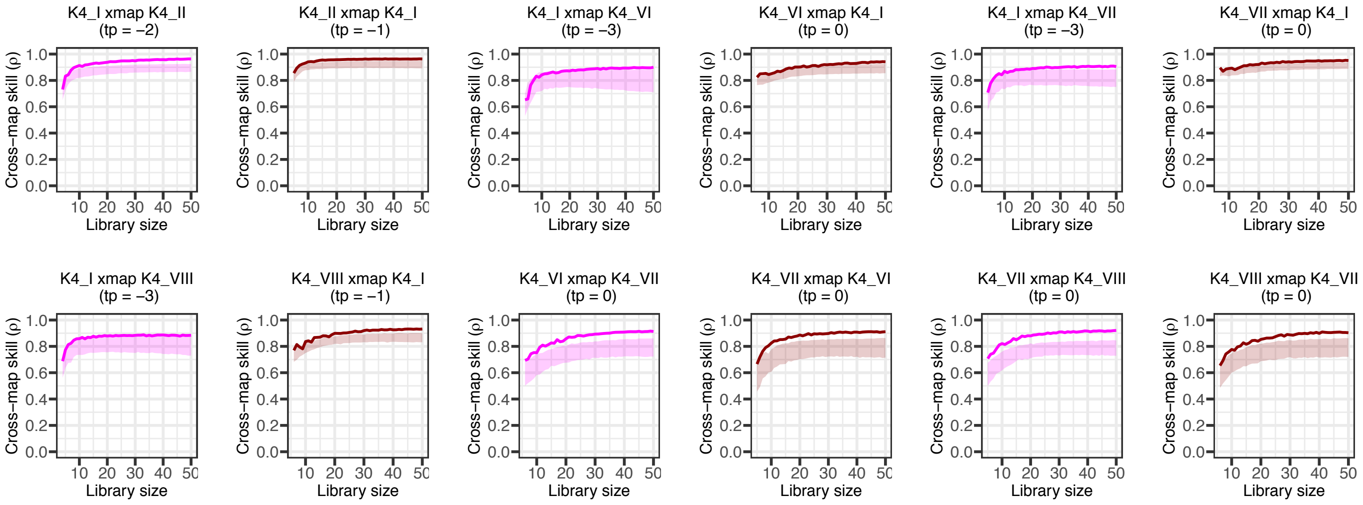

Supplementary Fig. 11 Convergent cross-mapping (CCM) between *AhgFLC* histone modification and mRNA levels. **a** CCM between H3K27me3 at amplicons I−VIII or H3K4me3 at amplicon I, and mRNA. **b** CCM between H3K27me3 and H3K4me3 at each amplicon. **c** CCM between H3K27me3 at adjacent amplicons. **d** CCM between H3K4me3 at amplicon I and other amplicons, and between H3K4me3 at adjacent amplicons. The cross-map skill (ρ) with the best time-to-prediction (tp) value is shown as the function of the library size (i.e. the number of time points used for prediction; see Methods). In the top keys, e.g. 'K27_I xmap K4_I' represents that K27_I cross-map (or cross-predict) K4_I, indicating that the state of K4_I is predicted using the state of K27_I. Because CCM explores the signature of a causal variable in an effect variable, this prediction measures the effect of K4_I on K27_I. Solid lines represent the cross-map skill (ρ), and shaded regions represent the 95% intervals of 100 seasonal-surrogate time series.

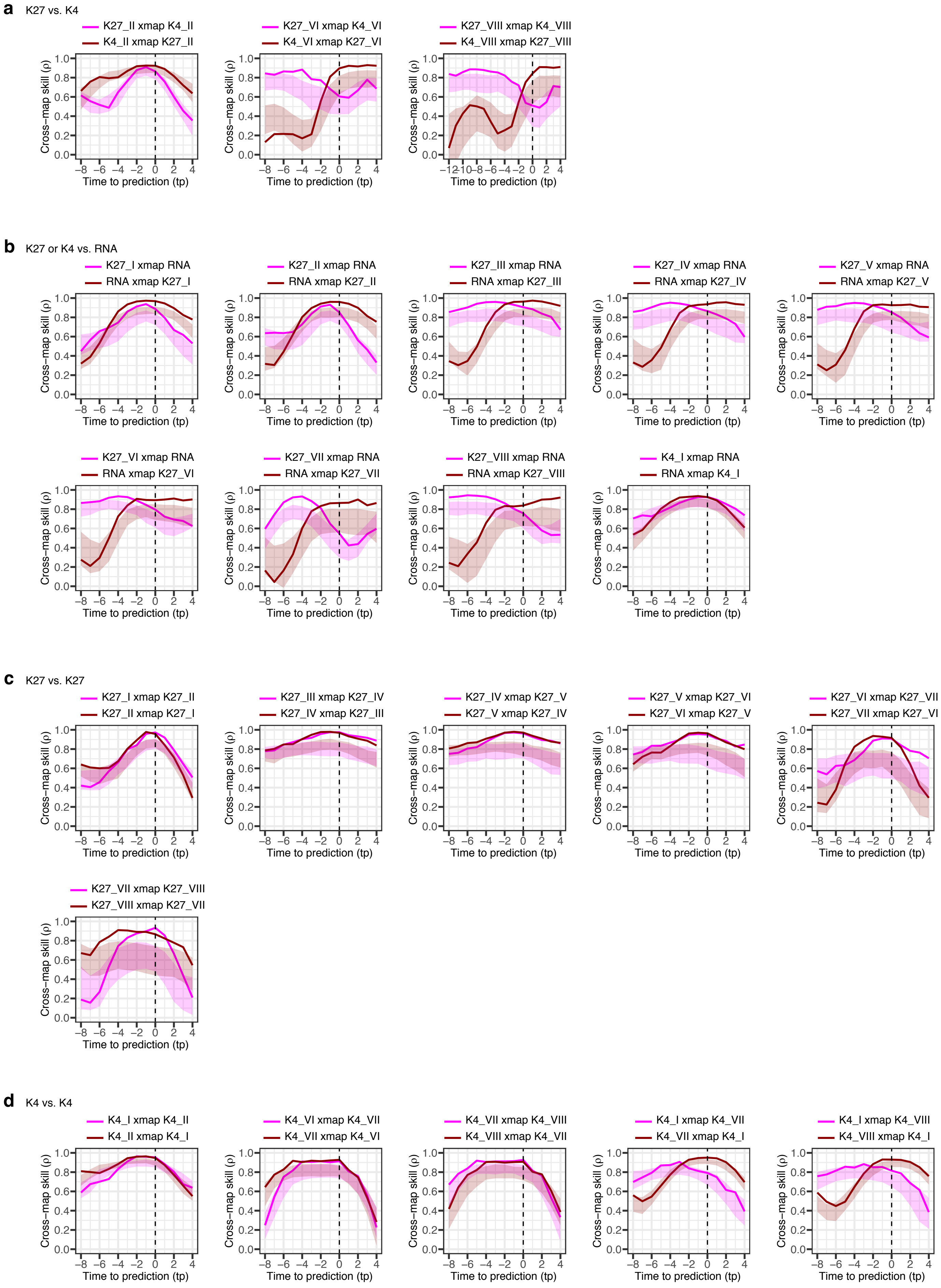
a K27 vs. K4
K27_II xmap K4_II
K4_II xmap K27_II
K27_VI xmap K4_VI
K4_VI xmap K27_VI
K27_VIII xmap K4_VIII
K4_VIII xmap K27_VIII
b K27 or K4 vs. RNA
K27_I xmap RNA
RNA xmap K27_I
K27_II xmap RNA
RNA xmap K27_II
K27_III xmap RNA
RNA xmap K27_III
K27_IV xmap RNA
RNA xmap K27_IV
K27_V xmap RNA
RNA xmap K27_V
K27_VI xmap RNA
RNA xmap K27_VI
K27_VII xmap RNA
RNA xmap K27_VII
K27_VIII xmap RNA
RNA xmap K27_VIII
K4_I xmap RNA
RNA xmap K4_I
c K27 vs. K27
K27_I xmap K27_II
K27_II xmap K27_I
K27_III xmap K27_IV
K27_IV xmap K27_III
K27_IV xmap K27_V
K27_V xmap K27_IV
K27_V xmap K27_VI
K27_VI xmap K27_V
K27_VI xmap K27_VII
K27_VII xmap K27_VI
K27_VII xmap K27_VIII
K27_VIII xmap K27_VII
d K4 vs. K4
K4_I xmap K4_II
K4_II xmap K4_I
K4_VI xmap K4_VII
K4_VII xmap K4_VI
K4_VII xmap K4_VIII
K4_VIII xmap K4_VII
K4_I xmap K4_VII
K4_VII xmap K4_I
K4_I xmap K4_VIII
K4_VIII xmap K4_I
Cross−map skill (ρ)
Time to prediction (tp)

Supplementary Fig. 12 Relationships between the cross-map skills (ρ) at the maximum library size and time to prediction (tp) for convergent cross-mapping (CCM) between *AhgFLC* histone modification and mRNA levels. **a** CCM between H3K27me3 at amplicons I−VIII or H3K4me3 at amplicon I, and mRNA. **b** CCM between H3K27me3 and H3K4me3 at each amplicon. **c** CCM between H3K27me3 at adjacent amplicons. **d** CCM between H3K4me3 at amplicon I and other amplicons, and between H3K4me3 at adjacent amplicons. The cross-map skill (ρ) is shown as the function of time to prediction (tp). In the top keys, e.g. 'K27_II xmap K4_II' represents that K27_II cross-map (or cross-predict) K4_II, indicating that the state of K4_II is predicted using the state of K27_II. Because CCM explores the signature of a causal variable in an effect variable, this prediction measures the effect of K4_II on K27_II. Solid lines represent the cross-map skill (ρ), and shaded regions represent the 95% intervals of 100 seasonal-surrogate time series.

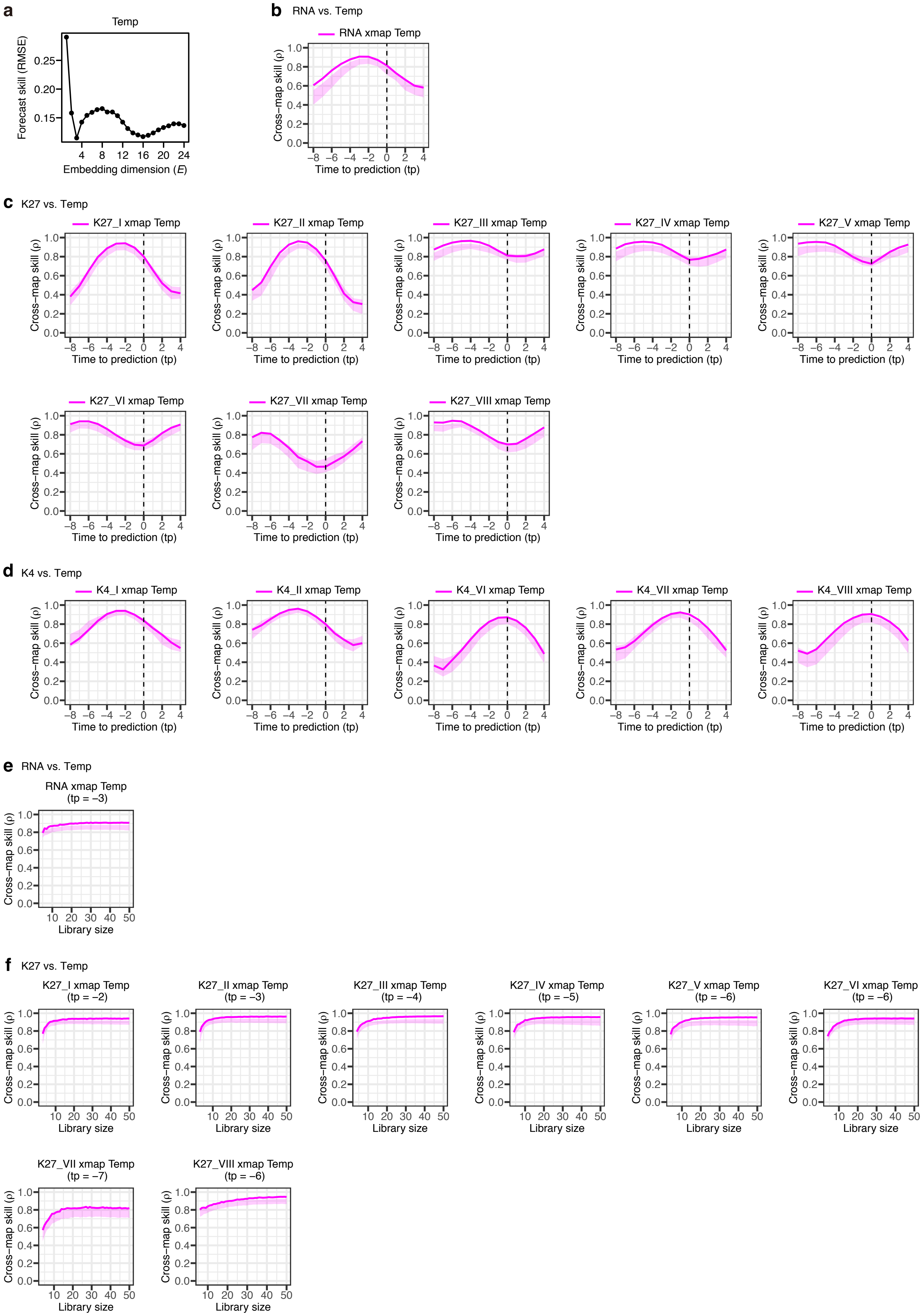
a
Temp
Forecast skill (RMSE)
Embedding dimension (E)
b RNA vs. Temp
RNA xmap Temp
Cross−map skill (ρ)
Time to prediction (tp)
c K27 vs. Temp
K27_I xmap Temp
K27_II xmap Temp
K27_III xmap Temp
K27_IV xmap Temp
K27_V xmap Temp
K27_VI xmap Temp
K27_VII xmap Temp
K27_VIII xmap Temp
d K4 vs. Temp
K4_I xmap Temp
K4_II xmap Temp
K4_VI xmap Temp
K4_VII xmap Temp
K4_VIII xmap Temp
e RNA vs. Temp
RNA xmap Temp (tp = −3)
Library size
f K27 vs. Temp
K27_I xmap Temp (tp = −2)
K27_II xmap Temp (tp = −3)
K27_III xmap Temp (tp = −4)
K27_IV xmap Temp (tp = −5)
K27_V xmap Temp (tp = −6)
K27_VI xmap Temp (tp = −6)
K27_VII xmap Temp (tp = −7)
K27_VIII xmap Temp (tp = −6)

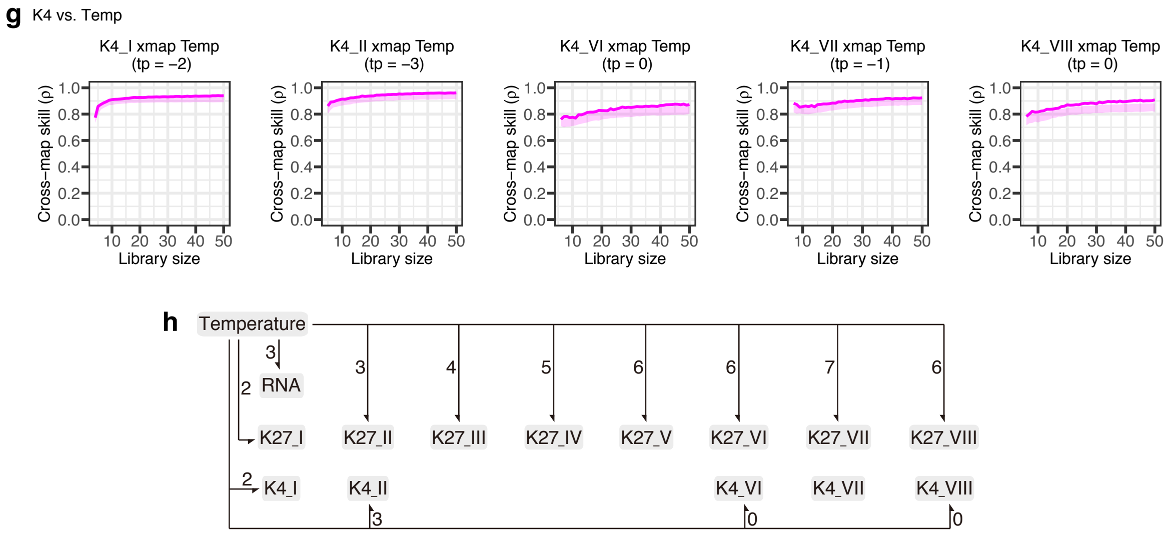

Supplementary Fig. 13 Empirical dynamic modelling for air temperature. **a** By changing *E* value from 1 to 24, we determined the best *E* value showing the minimum root mean squared error (RMSE) of prediction by using univariate simplex projection. The optimal *E* value for temperature was 3. **b**−**d** Relationships between the cross-map skills (ρ) at the maximum library size and effect time lags (i.e. time to prediction, tp) for convergent cross-mapping (CCM). **b** CCM between *AhgFLC* mRNA level and temperature. **c** CCM between H3K27me3 at each amplicon and temperature. **d** CCM between H3K4me3 at each amplicon and temperature. **e**−**g** The cross-map skill (ρ) with the best time-to-prediction (tp) value is shown as the function of the library size (i.e. the number of time points used for prediction; see Methods). **e** CCM between *AhgFLC* mRNA level and temperature. **f** CCM between H3K27me3 at each amplicon and temperature. **g** CCM between H3K4me3 at each amplicon and temperature. **b**−**g** In the top keys, e.g. 'RNA xmap temp' represents that RNA cross-map (or cross-predict) temperature, indicating that the state of temperature is predicted using the state of RNA. Because CCM explores the signature of a causal variable in an effect variable, this prediction measures the effect of temperature on RNA. Solid lines represent the cross-map skill (ρ), and shaded regions represent the 95% intervals of 100 seasonal-surrogate time series. **h** The causality from temperature to each of *AhgFLC* H3K27me3, H3K4me3, and mRNA, illustrated based on the results of CCM. Arrows represent the directions of causality. The umbers next to the arrows represent the time lags (1 time lag = 2 weeks).

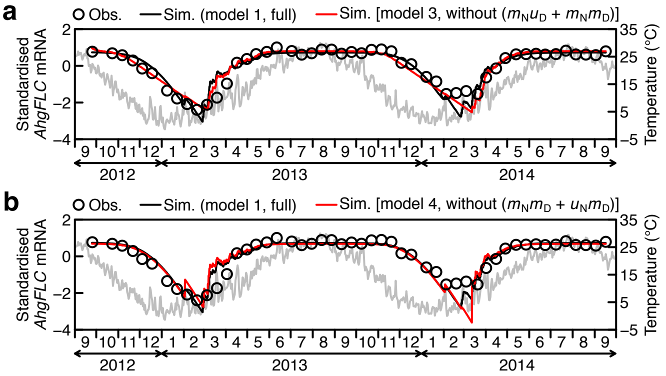

Supplementary Fig. 14 *AhgFLC* mRNA levels modelled by a linear regression with the H3K4me3 level at the nucleation region. **a** *AhgFLC* mRNA levels are compared between model 1 (full model with all three feedbacks; black) and model 3 that lacks the feedback effect from H3K27me3 to H3K4me3 at the nucleation region (red). **b** *AhgFLC* mRNA levels are compared between model 1 (black) and model 4 that lacks the feedback effect from H3K27me3 to H3K4me3 at the distal nucleation region (red). The simulated values (sim.) are shown with the observed values (obs.). The daily means of air temperature are plotted in grey. The observed values are shown as the means of four biological replicates and are represented by circles, whereas the simulated values are represented by lines.

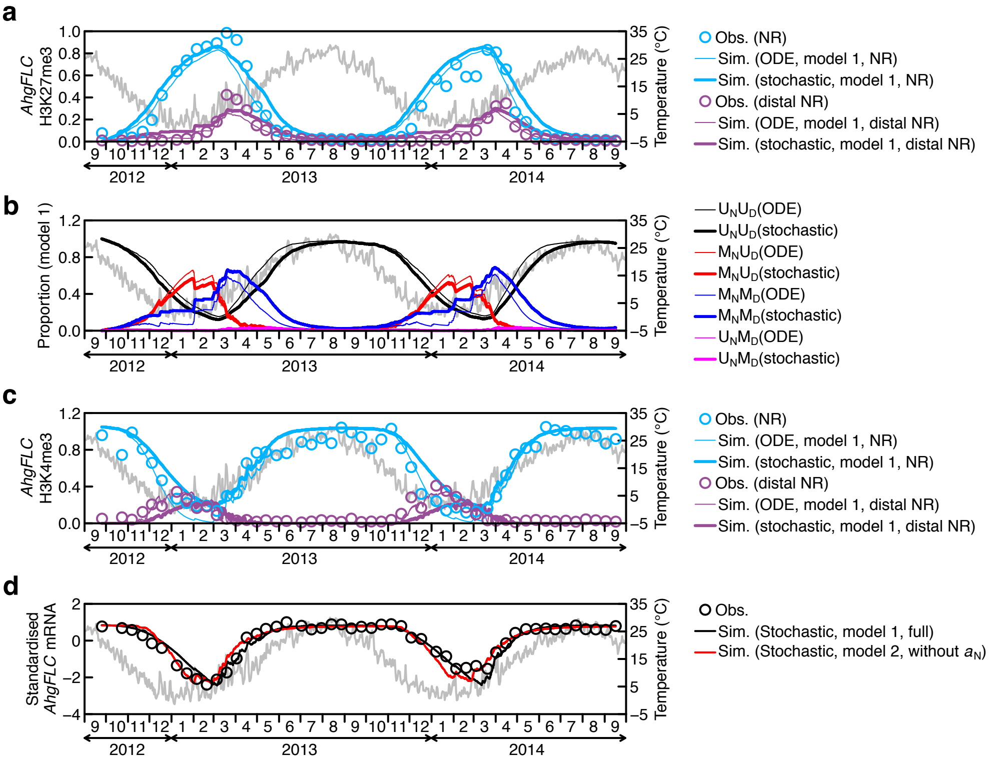

Supplementary Fig. 15 Stochastic modelling of *AhgFLC* histone modification and mRNA levels. **a** The observed (obs.) H3K27me3 levels and those simulated by the ordinary differential equation model (sim., ODE, model 1) and stochastic model (sim., stochastic, model 1) at the nucleation region (NR; blue) and the distal nucleation region (distal NR; purple) of *AhgFLC*. **b** The proportion of the four H3K27me3 states at *AhgFLC* in model 1 simulated by the ordinary differential equation model (ODE) and stochastic model (stochastic). **c** The observed (obs.) H3K4me3 levels and those simulated by the ordinary differential equation model (sim., ODE, model 1) and stochastic model (sim., stochastic, model 1) at the nucleation region (NR; blue) and the distal nucleation region (distal NR; purple) of *AhgFLC*. **d** *AhgFLC* mRNA levels modelled by a linear regression are compared between stochastic model 1 (full model with all three feedbacks; black) and stochastic model 2 that lacks the feedback effect of H3K4me3 on the $M_NM_D \rightarrow U_NM_D$ transition (red) and are shown with the observed values. In **a**–**d**, the daily means of air temperature are plotted in grey. The observed values are shown as the means of four biological replicates and are represented by circles, whereas the simulated values are represented by lines.

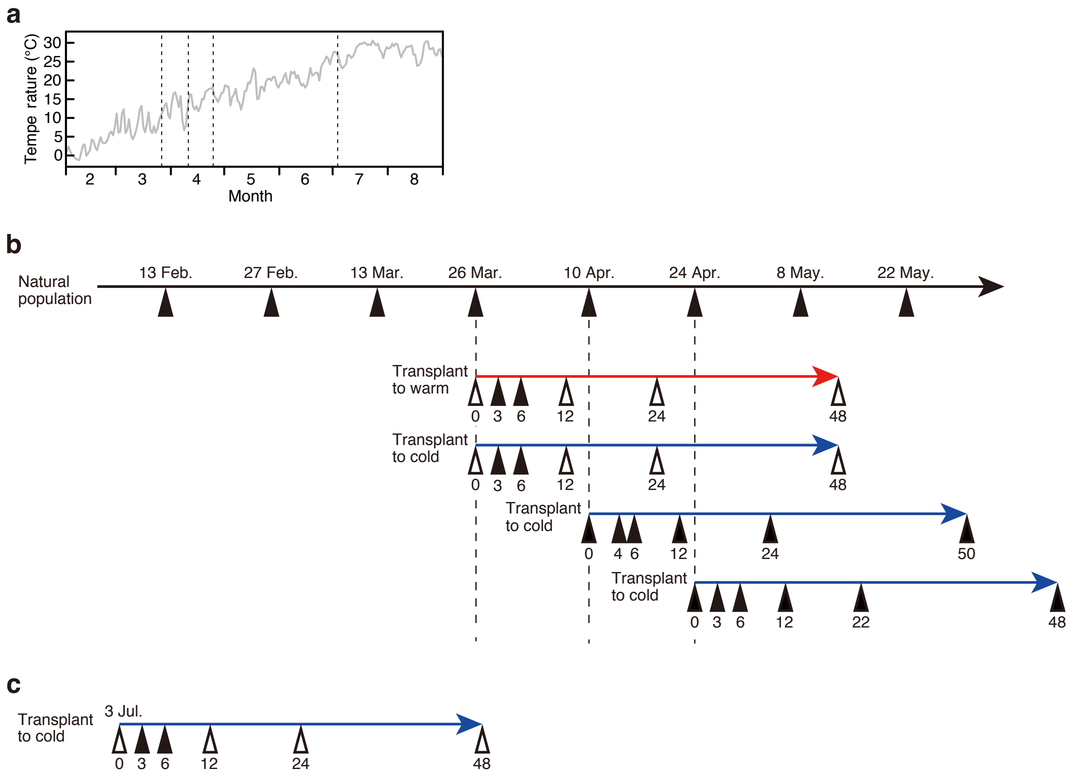

Supplementary Fig. 16 Experimental design of the transplant experiments. **a** The date of transplant (dashed lines) and daily means of air temperature measured at the meteorological station nearest to our field site (Nishiwaki, Hyogo) are shown. **b**, **c** The sampling schedule of plants in the natural population and in the transplant experiments [(**b**) 26 March, 10 April, and 24 April; (**c**) 3 July]. Growth periods in warm and cold conditions are indicated by red and blue arrows, respectively. Open triangles indicate the timing of sampling for ChIP and RNA extraction; filled triangles indicate that for only RNA extraction. The numbers below the triangles indicate the numbers of days from transplanting.

Supplementary Table 1 List of the primers used for qPCR and cloning.

Gene	Experiment	Primer sequence
AhgFLC amplicon I	ChIP-qPCR	5′-AAAAGGAAAGAGAAGAACGCTTAG-3′<br>5′-AGAGGGCTTTATTCCCTAGTTTG-3′
AhgFLC amplicon II	ChIP-qPCR	5′-GTCGCTCTTCTCGTCGTCTC-3′<br>5′-GGGAAATAAATGGAAACCCAGA-3′
AhgFLC amplicon III	ChIP-qPCR	5′-GAAAACCGACAATCACACAACC-3′<br>5′-TAGAGGCACCAAAGAAACAAGG-3′
AhgFLC amplicon IV	ChIP-qPCR	5′-ATGTCATCACTTTGTGGCTCATC-3′<br>5′-CATGTAAACGCAGCCGTAATC-3′
AhgFLC amplicon V	ChIP-qPCR	5′-TCTTAACGAGCTTGCACACATC-3′<br>5′-AAGACCGACACTTTCAGCAAATAC-3′
AhgFLC amplicon VI	ChIP-qPCR	5′-CGAATGGCTCAGTTCCAAAA-3′<br>5′-GGAAGCATGTACCTAAGCTTTTACC-3′
AhgFLC amplicon VII	ChIP-qPCR	5′-CGGCGGTTAAAATCAAAATC-3′<br>5′-CAAACGCTCGCCCTTATC-3′
AhgFLC amplicon VIII	ChIP-qPCR	5′-GTATGGTTGGCATGGGTTTTC-3′<br>5′-CCAAAGAGAAAACATGGAAAGAGG-3′
AhgFLC amplicon A	ChIP-qPCR	5′-TCTTTCCGGCGAATCTCTTG-3′<br>5′-AAACACCGAACCGGGAAAC-3′
AhgFLC amplicon B	ChIP-qPCR	5′-CCCGTAAGTACAAGTTCTAGCTCCA-3′<br>5′-AAGCTTTAGCTTCATCACGACATTG-3′
AhgFLC amplicon C	ChIP-qPCR	5′-CGTGCTCGATGTTGTTTAGTGAAG-3′<br>5′-GCATACAAATCCGAGAGATCCAA-3′
AhgFLC amplicon D	ChIP-qPCR	5′-TAGGATTGATATGGGGTTAATGCTG-3′<br>5′-CCAAGTAATAGGTCCACGAGAAAGA-3′
AhgSTM	ChIP-qPCR	5′-ATCAGTCACACCACCAACAAAGTAG-3′<br>5′-AGAGAATAAGCAGGGGCACAAG-3′
AhgACT2	ChIP-qPCR	5′-GCGACCAGACAGAGAAAGAAGG-3′<br>5′-GATGGAGAAAAGCGGAAGAAGA-3′
AhgFUS3	ChIP-qPCR	5′-ATTCTCAACGGAGCCCAAAC-3′<br>5′-AACCTCCAACGACACTCCTCTC-3′
AhgPP2AA3	ChIP-qPCR	5′-CAGCGTAATCGGTAAAATCTCG-3′<br>5′-CCGTCCCAATCTAGAGAGAAACTAC-3′
AhgFLC	RT-qPCR	5′-CGGCGGTTAAAATCAAAATC-3′<br>5′-CAAACGCTCGCCCTTATC-3′
AhgACT2	RT-qPCR	5′-TCCCTCAGCACATTCCAGCAGAT-3′<br>5′-AACGATTCCTGGACCTGCCTCATC-3′
AhgPP2AA3	RT-qPCR	5′-GTATGCACATGTTTTGCTTCCAC-3′<br>5′-CAACCAAGTCATTCTCCCTCATC-3′
AhgCOOLAIR Class I	Cloning	5′-GGTTGTTAGTCTTAATTGATGG-3′<br>5′-GCAAACACAAGTTTTGGACAGA-3′
AhgCOOLAIR Class II variant 1	Cloning	5′-GTATCTCCGGCGACTTGAAC-3′<br>5′-AGAGCCACGTTTCCTTTGCA-3′
AhgCOOLAIR Class II variant 2	Cloning	5′-GTATCTCCGGCGACTTGAAC-3′<br>5′-AGAGCCACGTTTCCTTTGCA-3′
AhgCOOLAIR Class II variant 3	Cloning	5′-GTATCTCCGGCGACTTGAAC-3′<br>5′-GCAAACACAAGTTTTGGACAGA-3′

Supplementary Table 2 List of the symbols used in the simulation and optimized parameter values.

Symbol	Description	Optimized values in the model 1 (full model)	Optimized values in the model 2 (without a_N)	Optimized values in the model 3 [without ($m_N u_D$+$m_N m_D$)]	Optimized values in the model 4 [without ($m_N m_D$+$u_N m_D$)]
$u_N u_D$	Proportion of the cells in which both two nucleation regions are not modified with H3K27me3				
$m_N u_D$	Proportion of the cells in which only the nucleation region is modified with H3K27me3				
$m_N m_D$	Proportion of the cells in which both two nucleation regions are modified with H3K27me3				
$u_N m_D$	Proportion of the cells in which only the distal nucleation region is modified with H3K27me3				
u_N, a_N	Proportion of the cells in which the nucleation region is not modified and is modified with H3K4me3, respectively				
u_D, a_D	Proportion of the cells in which the distal nucleation region is not modified and is modified with H3K4me3, respectively				
t	Time				
T	Temperature				
$\mu(T)$	Cold-dependent transition of $U_N U_D$-to-$M_N U_D$				
$\nu(T)$	Warm-dependent transition of $M_N U_D$-to-$M_N M_D$				
$\xi(T)$	Warm-dependent transition of U_N-to-A_N				
$\tau(T)$	Cold-dependent transition of U_D-to-A_D				
θ_1	Threshold temperature of $\mu(T)$	5.00	5.00	5.00	5.00
θ_2	Threshold temperature of $\nu(T)$	10.0	10.0	10.0	10.0
θ_3	Threshold temperature of $\xi(T)$	15.0	15.0	15.0	15.0
θ_4	Threshold temperature of $\tau(T)$	5.00	5.00	5.00	5.00
α	Slope of $\mu(T)$	0.158	4.68	1.10	0.161
β	Slope of $\nu(T)$	54.7	15.8	9.82	1.62
γ	Slope of $\xi(T)$	37.6	31.3	0.311	9.46
ε	Slope of $\tau(T)$	0.583	0.0533	10.6	2.77
ζ	Maximum value of $\mu(T)$	2.05	2.75	2.26	2.17
η	Maximum value of $\nu(T)$	9.12	9.45	8.61	8.54
ι	Maximum value of ξ(T)	35.3	29.1	16.1	41.8
ρ	Maximum value of $\tau(T)$	10.5	60.6	9.34	15.0
κ	Dependence on H3K4me3 in the demethylation of H3K27me3 at the nucleation region	5.54	1.64	3.53	5.54
λ	Demethylation rate of H3K27me3 at the distal nucleation region	28.0	5.17	7.42	12.8
ϕ	Dependence on H3K27me3 in the demethylation of H3K4me3 at the nucleation region	5.77	52.6	1.09	7.52
ψ	Dependence on H3K27me3 in the demethylation of H3K4me3 at the distal nucleation region	86.0	63.4	39.4	17.9
σ	Coefficient of the linear regression to model mRNA	3.33	3.33	3.33	3.33
ω	Intercept of the linear regression to model mRNA	0.244	0.244	0.244	0.244

Supplementary Note 1

Derivation of differential equation models from stochastic models

We derived differential equation models from stochastic models to link the tissue-level observations in experiments with the locus-level H3K27me3 and H3K4me3 states. The probabilities that an *AhgFLC* locus in a single cell is in the U_NU_D, M_NU_D, M_NM_D, and U_NM_D H3K27me3 states at time t were designated as $u_Nu_D(t)$, $m_Nu_D(t)$, $m_Nm_D(t)$, and $u_Nm_D(t)$, respectively. The probabilities that an *AhgFLC* locus in a single cell is in the A_N and A_D H3K4me3 states at time t were designated as $a_N(t)$ and $a_D(t)$, respectively.

Stochastic model

We define $S(t)$, $S_N(t)$, and $S_D(t)$ as the H3K27me3 state, the H3K4me3 state at the nucleation region, and the H3K4me3 state at the distal nucleation region at time t, respectively. As mentioned in the main text, we assumed that the H3K27me3 state changes along the path: $U_NU_D \to M_NU_D \to M_NM_D \to U_NM_D \to U_NU_D$. The transition probabilities of $S(t)$ within an infinitesimal time interval Δt, within which at most one state transition occurs, at temperature T are defined as:

$$\mathrm{P}[S(t+\Delta t) = \mathrm{M_N U_D} | S(t) = \mathrm{U_N U_D}] = \mu'(T, \Delta t), \quad (1)$$

$$\mathrm{P}[S(t+\Delta t) = \mathrm{M_N M_D} | S(t) = \mathrm{M_N U_D}] = \nu'(T, \Delta t), \quad (2)$$

$$\mathrm{P}[S(t+\Delta t) = \mathrm{U_N M_D} | S(t) = \mathrm{M_N M_D}] = \kappa'(\Delta t)\, I[S_N(t) = \mathrm{A_N}], \quad (3)$$

$$\mathrm{P}[S(t+\Delta t) = \mathrm{U_N U_D} | S(t) = \mathrm{U_N M_D}] = \lambda'(\Delta t), \quad (4)$$

where $I[S_N(t) = \mathrm{A_N}]$ is the indicator function defined as 1 if the *AhgFLC* locus at time t is in the A_N state, and 0 otherwise. Here, we assumed that the transitions $U_NU_D \to M_NU_D$ and $M_NU_D \to M_NM_D$ depend on temperatures.

The transition probabilities of $S_N(t)$ and $S_D(t)$ are defined as:

$$\mathrm{P}[S_N(t+\Delta t) = \mathrm{A_N} | S_N(t) = \mathrm{U_N}] = \xi'(T, \Delta t)\,(1 - I[S_D(t) = \mathrm{A_D}]), \quad (5)$$

$$\mathrm{P}[S_N(t+\Delta t) = \mathrm{U_N} | S_N(t) = \mathrm{A_N}] = \varphi'(\Delta t) \begin{pmatrix} I[S(t) = \mathrm{M_N U_D}] \\ +I[S(t) = \mathrm{M_N M_D}] \end{pmatrix}, \quad (6)$$

$$\mathrm{P}[S_{\mathrm{D}}(t+\Delta t)=\mathrm{A_D}|S_{\mathrm{D}}(t)=\mathrm{U_D}]=\tau'(T,\Delta t)\,(1-I[S_{\mathrm{N}}(t)=\mathrm{A_N}]), \quad \textbf{(7)}$$

$$\mathrm{P}[S_{\mathrm{D}}(t+\Delta t)=\mathrm{U_D}|S_{\mathrm{D}}(t)=\mathrm{A_D}]=\psi'(\Delta t)\begin{pmatrix} I[S(t)=\mathrm{M_N M_D}] \\ +I[S(t)=\mathrm{U_N M_D}] \end{pmatrix}. \quad \textbf{(8)}$$

Here, we assumed that H3K4me3 at the nucleation region and the distal nucleation region depend on each other.

Differential equation model

For ease of derivation of differential equation models, we assumed that the H3K27me3 state, the H3K4me3 state at the nucleation region, and the H3K4me3 state at the distal nucleation region follow:

$\mathrm{P}[S(t), S_{\mathrm{N}}(t), S_{\mathrm{D}}(t)] = \mathrm{P}[S(t)]\ \mathrm{P}[S_{\mathrm{N}}(t)]\ \mathrm{P}[S_{\mathrm{D}}(t)]$.

Therefore, we assumed that probabilities $\mathrm{P}[S(t)]$, $\mathrm{P}[S_{\mathrm{N}}(t)]$, and $\mathrm{P}[S_{\mathrm{D}}(t)]$ are independent. Although this model assumptions were strong, we confirmed that the results were similar between the original stochastic model and the differential equation model (described later in detail). The differential equations of all modification states were derived as below, separately.

$\mathrm{U_N U_D}$ state

The probability that an *AhgFLC* locus is in the $\mathrm{U_N U_D}$ state at a time ($t + \Delta t$) can be described as:

$$u_{\mathrm{N}}u_{\mathrm{D}}(t+\Delta t)=\lambda'(\Delta t)\,u_N m_{\mathrm{D}}(t)\ +\big(1-\mu'(T,\Delta t)\big)\,u_{\mathrm{N}}u_{\mathrm{D}}(t), \quad \textbf{(9)}$$

where the first term on the right side represents the probability of the $\mathrm{U_N M_D} \rightarrow \mathrm{U_N U_D}$ transition, and the second term represents the probability that $\mathrm{U_N U_D}$ remains unchanged. By subtracting $u_{\mathrm{N}}u_{\mathrm{D}}(t)$ from the both sides of equation (**9**), we get

$$u_{\mathrm{N}}u_{\mathrm{D}}(t+\Delta t)-u_{\mathrm{N}}u_{\mathrm{D}}(t)=\lambda'(\Delta t)\,u_N m_{\mathrm{D}}(t)-\mu'(T,\Delta t)\,u_{\mathrm{N}}u_{\mathrm{D}}(t). \quad \textbf{(10)}$$

Dividing the both sides of equation (**10**) by Δt and taking the limits $\Delta t \rightarrow 0$, we get

$$\frac{du_{\mathrm{N}}u_{\mathrm{D}}(t)}{dt}=\left.\frac{d\lambda'(\Delta t)}{d\Delta t}\right|_{\Delta t=0}u_{\mathrm{N}}m_{\mathrm{D}}(t)-\left.\frac{\partial\mu'(T,\Delta t)}{\partial\Delta t}\right|_{\Delta t=0}u_{\mathrm{N}}u_{\mathrm{D}}(t). \quad \textbf{(11)}$$

Setting $\left.\frac{d\lambda'(\Delta t)}{d\Delta t}\right|_{\Delta t=0}=\lambda$ and $\left.\frac{\partial\mu'(T,\Delta t)}{\partial\Delta t}\right|_{\Delta t=0}=\mu(T)$, we get the differential equation:

$$\frac{du_{\mathrm{N}}u_{\mathrm{D}}}{dt}=\lambda\,u_{\mathrm{N}}m_{\mathrm{D}}-\mu(T)\,u_{\mathrm{N}}u_{\mathrm{D}}\,. \quad \textbf{(12)}$$

M_NU_D state

The probability that an *AhgFLC* locus is in the M_NU_D state at a time ($t + \Delta t$) can be described as:

$$m_N u_D(t+\Delta t) = \mu'(T,\Delta t)\, u_N u_D(t) + \big(1-\nu'(T,\Delta t)\big)\, m_N u_D(t), \tag{13}$$

where the first term on the right side represents the probability of the $U_NU_D \rightarrow M_NU_D$ transition, and the second term represents the probability that M_NU_D remains unchanged. By subtracting $m_N u_D(t)$ from the both sides of equation (**13**), we get

$$m_N u_D(t+\Delta t) - m_N u_D(t) = \mu'(T,\Delta t)\, u_N u_D(t) - \nu'(T,\Delta t)\, m_N u_D(t). \tag{14}$$

Dividing the both sides of equation (**14**) by Δt and taking the limits $\Delta t \rightarrow 0$, we get

$$\frac{dm_N u_D(t)}{dt} = \left.\frac{\partial \mu'(T,\Delta t)}{\partial \Delta t}\right|_{\Delta t=0} u_N u_D(t) - \left.\frac{\partial \nu'(T,\Delta t)}{\partial \Delta t}\right|_{\Delta t=0} m_N u_D(t). \tag{15}$$

Setting $\left.\frac{\partial \mu'(T,\Delta t)}{\partial \Delta t}\right|_{\Delta t=0} = \mu(T)$ and $\left.\frac{\partial \nu'(T,\Delta t)}{\partial \Delta t}\right|_{\Delta t=0} = \nu(T)$, we get the differential equation:

$$\frac{dm_N u_D}{dt} = \mu(T)\, u_N u_D - \nu(T)\, m_N u_D\,. \tag{16}$$

M_NM_D state

We assumed that the probability of being in the M_NM_D state is independent of that of being in the A_N state. The probability that an *AhgFLC* locus is in the M_NM_D state at a time ($t + \Delta t$) can be described as:

$$\begin{aligned} m_N m_D(t+\Delta t) &= \nu'(T,\Delta t)\, m_N u_D(t) \\ &\quad + \Big(\big(1-a_N(t)\big) + (1-\kappa'(\Delta t))\, a_N(t)\Big)\, m_N m_D(t), \end{aligned} \tag{17}$$

where the first term on the right side represents the probability of the $M_NU_D \rightarrow M_NM_D$ transition, and the second term represents the probability that M_NM_D remains unchanged. By subtracting $m_N m_D(t)$ from the both sides of equation (**17**), we get

$$m_N m_D(t+\Delta t) - m_N m_D(t) = \nu'(T,\Delta t)\, m_N u_D(t) - \kappa'(\Delta t)\, a_N(t)\, m_N m_D(t). \tag{18}$$

Dividing the both sides of equation (**18**) by Δt and taking the limits $\Delta t \rightarrow 0$, we get

$$\frac{dm_N m_D(t)}{dt} = \left.\frac{\partial \nu'(T,\Delta t)}{\partial \Delta t}\right|_{\Delta t=0} m_N u_D(t) - \left.\frac{d\kappa'(\Delta t)}{d\Delta t}\right|_{\Delta t=0} a_N(t)\, m_N m_D(t). \tag{19}$$

Setting $\left.\frac{\partial \nu'(T,\Delta t)}{\partial \Delta t}\right|_{\Delta t=0} = \nu(T)$ and $\left.\frac{d\kappa'(\Delta t)}{d\Delta t}\right|_{\Delta t=0} = \kappa$, we get the differential equation:

$$\frac{dm_\mathrm{N}m_\mathrm{D}}{dt} = \nu(T)\, m_\mathrm{N}u_\mathrm{D} - \kappa\, a_\mathrm{N}\, m_\mathrm{N}m_\mathrm{D}\,. \quad \textbf{(20)}$$

U_NM_D state

We assumed that the probability of being in the M_NM_D state is independent of that of being in the A_N state. The probability that an *AhgFLC* locus is in the U_NM_D state at a time ($t + \Delta t$) can be described as:

$$u_\mathrm{N}m_\mathrm{D}(t+\Delta t) = \kappa'(\Delta t)\, a_\mathrm{N}(t)\, m_\mathrm{N}m_\mathrm{D}(t) + \big(1-\lambda'(\Delta t)\big)\, u_\mathrm{N}m_\mathrm{D}(t), \quad \textbf{(21)}$$

where the first term on the right side represents the probability of the M_NM_D→U_NM_D transition, and the second term represents the probability that U_NM_D remains unchanged. By subtracting $u_Nm_D(t)$ from the both sides of equation (**21**), we get

$$\begin{aligned} u_\mathrm{N}m_\mathrm{D}(t+\Delta t) - u_\mathrm{N}m_\mathrm{D}(t) &= \kappa'(\Delta t)\, a_\mathrm{N}(t)\, m_\mathrm{N}m_\mathrm{D}(t) \\ &\quad -\lambda'(\Delta t)\, u_\mathrm{N}m_\mathrm{D}(t). \end{aligned} \quad \textbf{(22)}$$

Dividing the both sides of equation (**22**) by Δt and taking the limits $\Delta t \to 0$, we get

$$\frac{du_\mathrm{N}m_\mathrm{D}(t)}{dt} = \left.\frac{d\kappa'(\Delta t)}{d\Delta t}\right|_{\Delta t=0} a_\mathrm{N}(t)\, m_\mathrm{N}m_\mathrm{D}(t) - \left.\frac{d\lambda'(\Delta t)}{d\Delta t}\right|_{\Delta t=0} u_\mathrm{N}m_\mathrm{D}(t). \quad \textbf{(23)}$$

Setting $\left.\frac{d\kappa'(\Delta t)}{d\Delta t}\right|_{\Delta t=0} = \kappa$ and $\left.\frac{d\lambda'(\Delta t)}{d\Delta t}\right|_{\Delta t=0} = \lambda$, we get the differential equation:

$$\frac{du_\mathrm{N}m_\mathrm{D}}{dt} = \kappa\, a_\mathrm{N}\, m_\mathrm{N}m_\mathrm{D} - \lambda\, u_\mathrm{N}m_\mathrm{D}\,. \quad \textbf{(24)}$$

A_N state

We assumed that the probability of being in the A_N state is independent of those of being in the M_NU_D, M_NM_D and A_D states. The probability that the nucleation region at an *AhgFLC* locus is in the A_N state at a time ($t + \Delta t$) can be described as:

$$\begin{aligned} a_\mathrm{N}(t+\Delta t) &= \xi'(T,\Delta t)\, \big(1-a_\mathrm{D}(t)\big)\, u_\mathrm{N}(t) \\ &\quad + \begin{pmatrix} \big(1-m_\mathrm{N}u_\mathrm{D}(t)-m_\mathrm{N}m_\mathrm{D}(t)\big) \\ +\big(1-\varphi'(\Delta t)\big)\big(m_\mathrm{N}u_\mathrm{D}(t)+m_\mathrm{N}m_\mathrm{D}(t)\big) \end{pmatrix} a_\mathrm{N}(t), \end{aligned} \quad \textbf{(25)}$$

where the first term on the right side represents the probability of the U_N→A_N transition, and the second term represents the probability that A_N remains unchanged. By subtracting $a_N(t)$ from the both sides of equation (**25**), we get

$$\begin{aligned} a_\mathrm{N}(t+\Delta t) - a_\mathrm{N}(t) &= \xi'(T,\Delta t)\, \big(1-a_\mathrm{D}(t)\big)\, u_\mathrm{N}(t) \\ &\quad -\varphi'(\Delta t)\big(m_\mathrm{N}u_\mathrm{D}(t)+m_\mathrm{N}m_\mathrm{D}(t)\big)\, a_\mathrm{N}(t). \end{aligned} \quad \textbf{(26)}$$

Dividing the both sides of equation (**26**) by Δt and taking the limits $\Delta t \to 0$, we get

$$\frac{da_{\mathrm{N}}(t)}{dt} = \left.\frac{\partial \xi'(T,\Delta t)}{\partial \Delta t}\right|_{\Delta t=0} \big(1 - a_{\mathrm{D}}(t)\big)\, u_{\mathrm{N}}(t) - \left.\frac{d\varphi'(\Delta t)}{d\Delta t}\right|_{\Delta t=0} \big(m_{\mathrm{N}} u_{\mathrm{D}}(t) + m_{\mathrm{N}} m_{\mathrm{D}}(t)\big)\, a_{\mathrm{N}}(t). \quad \mathbf{(27)}$$

Setting $\left.\frac{\partial \xi'(T,\Delta t)}{\partial \Delta t}\right|_{\Delta t=0} = \xi(T)$ and $\left.\frac{d\varphi'(\Delta t)}{d\Delta t}\right|_{\Delta t=0} = \varphi$, we get the differential equation:

$$\frac{da_{\mathrm{N}}}{dt} = \xi(T)(1 - a_{\mathrm{D}})\, u_{\mathrm{N}} - \varphi(m_{\mathrm{N}} u_{\mathrm{D}} + m_{\mathrm{N}} m_{\mathrm{D}})\, a_{\mathrm{N}}. \quad \mathbf{(28)}$$

A_D state

We assumed that the probability of being in the A_D state is independent of those of being in the $M_N M_D$, $U_N M_D$ and A_N states. The probability that the distal nucleation region at an *AhgFLC* locus is in the A_D state at a time ($t + \Delta t$) can be described as:

$$a_{\mathrm{D}}(t + \Delta t) = \tau'(T,\Delta t)\,\big(1 - a_{\mathrm{N}}(t)\big)\, u_{\mathrm{D}}(t) + \begin{pmatrix} \big(1 - m_{\mathrm{N}} m_{\mathrm{D}}(t) - u_{\mathrm{N}} m_{\mathrm{D}}(t)\big) \\ + \big(1 - \psi'(\Delta t)\big)\big(m_{\mathrm{N}} m_{\mathrm{D}}(t) + u_{\mathrm{N}} m_{\mathrm{D}}(t)\big) \end{pmatrix} a_{\mathrm{D}}(t), \quad \mathbf{(29)}$$

where the first term on the right side represents the probability of the $U_D \to A_D$ transition, and the second term represents the probability that A_D remains unchanged. By subtracting $a_D(t)$ from the both sides of equation (**29**), we get

$$a_{\mathrm{D}}(t + \Delta t) - a_{\mathrm{D}}(t) = \tau'(T,\Delta t)\,\big(1 - a_{\mathrm{N}}(t)\big)\, u_{\mathrm{D}}(t) - \psi'(\Delta t)\big(m_{\mathrm{N}} m_{\mathrm{D}}(t) + u_{\mathrm{N}} m_{\mathrm{D}}(t)\big)\, a_{\mathrm{D}}(t). \quad \mathbf{(30)}$$

Dividing the both sides of equation (**30**) by Δt and taking the limits $\Delta t \to 0$, we get

$$\frac{da_{\mathrm{D}}(t)}{dt} = \left.\frac{\partial \tau'(T,\Delta t)}{\partial \Delta t}\right|_{\Delta t=0} \big(1 - a_{\mathrm{N}}(t)\big)\, u_{\mathrm{D}}(t) - \left.\frac{d\psi'(\Delta t)}{d\Delta t}\right|_{\Delta t=0} \big(m_{\mathrm{N}} m_{\mathrm{D}}(t) + u_{\mathrm{N}} m_{\mathrm{D}}(t)\big)\, a_{\mathrm{D}}(t). \quad \mathbf{(31)}$$

Setting $\left.\frac{\partial \tau'(T,\Delta t)}{\partial \Delta t}\right|_{\Delta t=0} = \tau(T)$ and $\left.\frac{d\psi'(\Delta t)}{d\Delta t}\right|_{\Delta t=0} = \psi$, we get the differential equation:

$$\frac{da_{\mathrm{D}}}{dt} = \tau(T)(1 - a_{\mathrm{N}})\, u_{\mathrm{D}} - \psi(m_{\mathrm{N}} m_{\mathrm{D}} + u_{\mathrm{N}} m_{\mathrm{D}})\, a_{\mathrm{D}}\,. \quad \mathbf{(32)}$$

Evaluation of the model assumptions

To evaluate the influence of the model assumptions on our conclusion, we performed the simulation of the original stochastic models (equations **1**−**8**) using the parameters optimised for

the differential equation models (Supplementary Fig. 15). We performed the simulation for 10,000 times in which the H3K27me3 and H3K4me3 states at each individual *AhgFLC* locus change with the probabilities defined in equations **1**−**8** at one hour intervals. We confirmed that the results were similar between the stochastic model and the differential equation model for the H3K27me3 state. In the stochastic model, the H3K4me3 dynamics were delayed relative to those in the differential equation model at both the nucleation and distal nucleation regions. However, in the stochastic model, the mRNA level in model 1 agreed well with the observed data, and the difference in the mRNA dynamics between models 1 and 2 was similar to that in the differential equation model. Thus, we confirmed that the influence of the model assumptions on our conclusion of the modelling was minimal.
