# Supplementary material for: Repressive chromatin modification underpins the long-term expression trend of a perennial flowering gene in nature
Source: Nat Commun. 2020 May 1;11:2065. doi: 10.1038/s41467-020-15896-4 (PMC7195410; doi:10.1038/s41467-020-15896-4)
Supplement: Supplementary file 3 — Reporting summary [file 41467_2020_15896_MOESM3_ESM.pdf]

nature research

Corresponding author(s): Haruki Nishio & Hiroshi Kudoh

Last updated by author(s): Mar 30, 2020

Reporting Summary

Nature Research wishes to improve the reproducibility of the work that we publish. This form provides structure for consistency and transparency in reporting. For further information on Nature Research policies, see Authors & Referees and the Editorial Policy Checklist .

Statistics

For all statistical analyses, confirm that the following items are present in the figure legend, table legend, main text, or Methods section.

n/a	Confirmed	
☐	☒	The exact sample size (*n*) for each experimental group/condition, given as a discrete number and unit of measurement
☐	☒	A statement on whether measurements were taken from distinct samples or whether the same sample was measured repeatedly
☐	☒	The statistical test(s) used AND whether they are one- or two-sided<br>*Only common tests should be described solely by name; describe more complex techniques in the Methods section.*
☐	☒	A description of all covariates tested
☐	☒	A description of any assumptions or corrections, such as tests of normality and adjustment for multiple comparisons
☐	☒	A full description of the statistical parameters including central tendency (e.g. means) or other basic estimates (e.g. regression coefficient) AND variation (e.g. standard deviation) or associated estimates of uncertainty (e.g. confidence intervals)
☐	☒	For null hypothesis testing, the test statistic (e.g. *F*, *t*, *r*) with confidence intervals, effect sizes, degrees of freedom and *P* value noted<br>*Give P values as exact values whenever suitable.*
☒	☐	For Bayesian analysis, information on the choice of priors and Markov chain Monte Carlo settings
☒	☐	For hierarchical and complex designs, identification of the appropriate level for tests and full reporting of outcomes
☐	☒	Estimates of effect sizes (e.g. Cohen's *d*, Pearson's *r*), indicating how they were calculated

Our web collection on statistics for biologists contains articles on many of the points above.

Software and code

Policy information about availability of computer code

Data collection	To obtain ChIP-/RT- qPCR data, 7300 System SDS Software v1.3 was used.
Data analysis	To perform statistical analyses and generate graphs, R v3.2.1 was used. The detailed explanations are given in the Methods section.<br>R codes used in this study are available at http://sohi.ecology.kyoto-u.ac.jp/AhgRNAseq/Nishio_script_200330.zip.

For manuscripts utilizing custom algorithms or software that are central to the research but not yet described in published literature, software must be made available to editors/reviewers. We strongly encourage code deposition in a community repository (e.g. GitHub). See the Nature Research guidelines for submitting code & software for further information.

Data

Policy information about availability of data

All manuscripts must include a data availability statement. This statement should provide the following information, where applicable:

- Accession codes, unique identifiers, or web links for publicly available datasets
- A list of figures that have associated raw data
- A description of any restrictions on data availability

All data in this study is available at http://sohi.ecology.kyoto-u.ac.jp/AhgRNAseq/Nishio_script_200330.zip.

Field-specific reporting

Please select the one below that is the best fit for your research. If you are not sure, read the appropriate sections before making your selection.

☒ Life sciences ☐ Behavioural & social sciences ☐ Ecological, evolutionary & environmental sciences

Life sciences study design

All studies must disclose on these points even when the disclosure is negative.

Sample size	For field samples, we determined the number of study plants to be the maximum in terms of representing the whole population (see the Randomization section) while minimizing the whole sampling time (<1.5 hour). We previously confirmed that reliable data can be produced within two hours of sampling (Nishio et al., 2016 Genes Genet. Syst. 91, 15). Then we determined 40 plants on each sampling date to fulfill both requirements. To assure the DNA amount enough for ChIP-qPCR, leaves from ten individuals were pooled. For both RT-qPCR and ChIP-qPCR, biweekly two-year samples were analysed to achieve sufficiently high temporal resolution to apply modelling approaches: linear regression analyses, empirical dynamic modelling, and mathematical modelling.
Data exclusions	No data were excluded in this study.
Replication	RT-qPCR of AhgFLC for naturally growing plants:<br>Four replicates per sampling date.<br>For each replicate, one leaf from a single plant was analysed.<br><br>RT-qPCR of AhgFLC for transplanted plants:<br>Three replicates per sampling date.<br>For each replicate, one leaf from a single plant was analysed.<br><br>ChIP-qPCR of AhgFLC for naturally growing plants:<br>Four replicates per sampling date for H3K4me3 and H3K27me3 at amplicons I–V.<br>Three or four replicates (average, >3.9) per sampling date for H3K4me3 and H3K27me3 at amplicons VI–VIII.<br>For each replicate, a pool of leaves from ten plants (out of 40 plants) was analysed.<br><br>ChIP-qPCR of AhgFLC for transplanted plants:<br>Three replicates per sampling date for H3K4me3 and H3K27me3 at all amplicons.<br>For each replicate, a pool of leaves from two plants (out of six plants) was analysed.<br><br>The qPCR experiments were performed in duplicates for all samples. To verify the reproducibility of the qPCR experiments, we set the measure in which the differences in the Ct values between qPCR duplicates have to be under 0.8. The values from duplicates were averaged to represent one sample. For the samples which did not meet this measure, we repeated the qPCR experiments.
Randomization	The sampled plants were selected to represent the natural populations. We set three rectangular plots (ca. 20 m × 40 m for each) in the study site. Pooled plants in each replicate were chosen from the three plots in proportion to the density of plants within the plots. When it is required to select multiple plants per plot, we chose plants far from each other as much as possible.<br>Time-series samples were analysed in a replicate-base order to control covariates, that is, to randomize experiment-wise bias. For example, replicate 1 for eight months were handled in an experiment rather than handling all replicates for a particular month.
Blinding	Investigators were not blinded to group allocation during experiments, to follow the randomization strategy described above.

Reporting for specific materials, systems and methods

We require information from authors about some types of materials, experimental systems and methods used in many studies. Here, indicate whether each material, system or method listed is relevant to your study. If you are not sure if a list item applies to your research, read the appropriate section before selecting a response.

Materials & experimental systems

n/a	Involved in the study	
☐	☒	Antibodies
☒	☐	Eukaryotic cell lines
☒	☐	Palaeontology
☒	☐	Animals and other organisms
☒	☐	Human research participants
☒	☐	Clinical data

Methods

n/a	Involved in the study	
☒	☐	ChIP-seq
☒	☐	Flow cytometry
☒	☐	MRI-based neuroimaging

Antibodies

Antibodies used	For ChIP, following three antibodies were used.<br>anti-H3K27me3 (07-449, Millipore, rabbit polyclonal, dilution 1:500)<br>anti-H3K4me3 (07-473, Millipore, rabbit polyclonal, dilution 1:500)

anti-H3 (ab1791, Abcam, rabbit polyclonal, dilution 1:1000)

Validation

All antibodies used in this study is commercially available and have been validated for application to ChIP by suppliers and in the referenced studies on the suppliers' homepages: https://www.merckmillipore.com/JP/ja/product/Anti-trimethyl-Histone-H3-Lys27-Antibody,MM_NF-07-449 (anti-H3K27me3), https://www.merckmillipore.com/JP/ja/product/Anti-trimethyl-Histone-H3-Lys4-Antibody,MM_NF-07-473 (anti-H3K4me3), and https://www.abcam.com/epigenetics/advantages-of-our-anti-histone-h3-antibody (anti-H3). In the supplier's homepages, there are validation statements, "Anti-trimethyl-Histone H3 (Lys27), also known as Anti-H3K27me3, is a highly published Rabbit Polyclonal Antibody. This protein A purified antibody is dot blot tested for trimethylated lysine 27 specificity and validated in WB, ICC, IP", "Anti-trimethyl-Histone H3 (Lys4) Antibody is a rabbit polyclonal antibody for detection of Histone H3 trimethylated at lysine 4. Also known as Anti-H3K4me3, this highly specific and well published antibody has been validated in ChIP, DB, WB, PIA, ChIP-seq", "Cited in hundreds of publications and validated in multiple applications and species, our anti-histone H3 antibody (ab1791) is the gold standard for histone H3 research. No need for addition validation; get straight to your research. Tested in ChIP, WB, IHC and ICC. See our data". These antibodies were used for ChIP in Arabidopsis in many studies, for example, Angel et al., 2011 Nature (anti-H3K27me3 and anti-H3), Yang et al., 2015 Nat. Commun. (anti-H3K27me3), Kang et al., 2015 Sci. Rep. (anti-H3K27me3 and anti-H3K4me3), and Liu et al., 2019 Plant Cell (anti-H3K4me3 and anti-H3).
