# Supplementary material for: Repressive chromatin modification underpins the long-term expression trend of a perennial flowering gene in nature
Source: Nat Commun. 2020 May 1;11:2065. doi: 10.1038/s41467-020-15896-4 (PMC7195410; doi:10.1038/s41467-020-15896-4)
Supplement: Supplementary file 2 — Peer Review [file 41467_2020_15896_MOESM2_ESM.pdf]

Reviewers' comments:

Reviewer #1 (Remarks to the Author):

This is an interesting paper showing a very large and impressive dataset with extensive analysis that is well placed in the context of the recent literature. The data is consistent with current knowledge and there are also some surprising and exciting findings. In most cases, the conclusions are well supported by the data in a clear way. I do comment on one case (comment on lines 257-259, 267-269) where the connection between the results and conclusion is not clear in the text. There is also a possible error in the conceptual underpinning of the mathematical model (see comment on Fig.5a below) that needs to be addressed. I also include some more minor comments below.

Comments on scientific content:

line 110: The authors say K4 "followed" the mRNA, but actually K4 seems to drop earlier in Fig.1b,c.

line 149: what do the authors mean by the period being "colinear"?

line 169: What is meant by the word "advanced" in this sentence? Is this related to the above point? In general, for this section on Lissajous curves, what has been learned here that could not have been learned from a detailed inspection of Fig. 1?

lines 208-212: Is the UM state necessary for the model? Is it reasonable? Also based on line 117 earlier, the drop in K27 is synchronised, which seems not to support the existence of this state.

Fig.5a: The transition MM->UM depends on transcription but according to the way it is defined in the model, transcription in trans is assumed to affect this transition. This means that transcription from other loci (and also in other cells) is somehow affecting the removal of K27 at the MM loci. Previous work has shown links to transcription but this is "in cis", meaning at the same locus.

lines 228-238: I don't really understand this paragraph. More intuition would be helpful. Furthermore, the model with/without feedback does not seem to be fully defined anywhere.

lines 247-248: What is this speculation based on? If it is based on Fig.4c, that is not clear in the text and it should be stated and explained.

line 254: Did K4 really decrease? It seems to be noisy (going first up then down), without a clear trend.

line 255-257: K4 in distal nucleation region is reported to respond to cold earlier than at the nucleation region, but in Fig.1 it seems to respond later.

lines 257-259, 267-269: What is the evidence of the H3K27 role here? There is no reference to the CCM results; is that the evidence? I would instead suggest postponing speculation about H3K27 (and COOLAIR, as you already have done) to the discussion section.

SMA analysis: This analysis is using only mean temperatures. But in A. thaliana it was shown that daily maxima and minima are key in temperature sensing for vernalization. Could you comment on this choice?

EDM-CCM analysis: This is described clearly and seems reasonable but, for full disclosure, I am not an expert on this type of modelling.

line 328-329: How was it completed in half an hour if it started at 12 and finished at 13:30? Not 1.5h?

Fig.4c: If this panel is important, you could make it a bit bigger and clearer.

lines 230-233: Sentence unclear.

Comments on language and typographical errors:

line 30: “Furthermore, we show that the long-term…”
line 80: “revealed” -> “measured”?
line 95: “enables”
lines 148: “best explained” (no "the")
line 172: “performed a causality”
line 229: “affects”
line 235: “best explained” (no "the")
line 238: “to the long-term”
line 242: “similarity”
line 303: “fluctuations”

Reviewer #2 (Remarks to the Author):

FLC is a potent regulator of flowering time in both annual and perennial species within the Brassicacae. In this manuscript the authors have carried out a comprehensive analysis of FLC expression and chromatin environment, in the perennial Arabidopsis halleri, over a 2 year period under natural conditions. Modelling demonstrated that there is a bi-directional relationship between transcription and H3K37me3 modification of AhgFLC. In addition, marking of AhgFLC chromatin with H3K27me3 allows reversible FLC expression over the life cycle of this perennial plant but ensures that transcription of AhgFLC is insensitive to cold temperatures during the up-regulation phase in spring. This is a nice piece of work using plants that are grown under natural conditions over two years, as well as experiments that perturb the normal seasonal changes in temperature allowing the authors to gain some novel insights into the regulation of the much studied FLC locus over the natural life cycle. These experiments were done in a perennial species, and so contribute to our understanding of how the perennial life cycle is regulated. I have a few criticisms of the manuscript which I have outlined below:
1. I am concerned by the use of Arabidopsis as an abbreviation in both the title and throughout the text as this I misleading. While A. halleri is a member of the Arabidopsis genus its life cycle is very different from that of A. thaliana with which most readers will be more familiar. For clarity I strongly recommend that the authors stick with the nomenclature A. halleri throughout.
2. P7-8, “Function of AhgFLC H2K27me3 in the seasonal gene regulation”; in this section the author use a mathematical model to predict H3K27me3 and mRNA dynamics under seasonal variation in temperature. They assume that transcription enhances the removal of H3K27me3 and that there are unidirectional circular transitions between the four modification states (UU to MU to MM to UM to UU). The predicted H3K27me3 levels agree well with observed data leading the authors to conclude that their “simple model explains the major dynamic over weeks without previously assumed interaction with active modifications”. I may be missing something but as transcriptional activity is closely linked with the level of H3K4me3 at the nucleation region (see Fig 1 b,c) I do not think that the authors can exclude the possibility that it is the level of H3K4me3, rather than transcription per se, that influences H3K27me3 levels. The Lissajous plots for HK4me3 (amplicons 1 & 2) against mRNA support my comments.
3. I strongly recommend that the authors provide Lissajous curves for the correlation between H3K4me3 (at the nucleation region) and H3K27me3.
4. I suggest that the authors define more explicitly the abbreviations UU, MU, MM and UM so that it is clear what they refer to. Initially I wondered if the two letters referred to the status of H3 at the two

FLC alleles, or to the two different molecules of H3 in a single nucleosome but finally decided that the first letter probably refers to the status of H3 modification in the nucleation region and the second letter to the linker region.
5. The significance of the second conclusion – that H3K27me3 at the distal nucleation region prevents downregulation of AhgFLC during cold weather in spring is relevant to perennial rather than annual species of Arabidopsis. This needs to be clearly stated as it is the seasonal upregulation of FLC after winter that is essential for a perennial life cycle.
6. P8, line 236; Here the authors refer to Fig 5e, but there is no such figure in the version I received.
7. One wonders why the authors did not check COOLAIR transcription (P10 lines 293-299) given that they had RNA available to do this.
8. I don’t really understand what the authors are proposing in the last sentence – please explain or delete.
Minor comments:
P9 line 281; I suggest replacing the word ‘innate” with “endogenous”

Reviewer #3 (Remarks to the Author):

The manuscript by Nishio and colleagues describes an exceptional data set: the authors have conducted a long term (2-years) field study on a natural population of the perennial plant species Arabidopsis halleri and collected in a fine-grained time course samples for chromatin immunoprecipitation and expression analysis. The authors focussed on the kinetics of A. halleri FLC, measuring expression and the dynamics of H3K27me3 and H3K4me3 across the locus. The authors then analysed their data by mathematical approaches that include linear regression comparison between measured traits and windows of past temperature, empirical dynamic modelling using established causality test and ODE-based modelling of chromatin states. Despite the real interesting dataset, the modelling approach is a little disappointing in terms of novelty and doesn’t seem to give full justice to the great data set. Maybe the authors can go back to the model, or explain the points listed below to my satisfaction.
FLC is a well-studied locus in the annual model A. thaliana, but has also been studied in perennial Brassica species. The modelling approaches confirmed existing models and predictions on the A. thaliana FLC locus concerning down-regulation of the gene in response to cold temperatures (vernalization). Novel aspects that are typical to perennial FLC regulation would be a better understanding of the processes involved in FLC upregulation after flowering. The authors describe a distal nucleation region, located downstream of FLC that may correspond to the promoter/TSS of the anti-sense transcript COOLAIR. Unfortunately, the ODE approach excludes the H3K4me3 modification, which fluctuates at the distal nucleation region; therefore the observation is not actually addressed in the mathematical model.
Major points:
1. Paragraph on co-linearity between regions and temperature medians across windows. Everything is quite coherent until the conclusion. Different temperature sensors are implied for both nucleation regions. The proximal region appears to react to a short-term trend while TSS nucleation and spreading integrates long-term trends. That is rather interesting and could mean that if cold periods are interrupted often enough, the COOLAIR promoter cannot fire anymore to prevent FLC upregulation, possibly because a specific transcription factor is not present. But why go from there to the statement “—we conclude that the H3K27me3 levels are indexing the seasonal temperature throughout the year…” where is the novelty in this statement?
2. Analysis of Lissajou’s curves for H3K4me3 at the distal nucleation region suggests that H3K4me3 dynamics advance FLC expression levels. CCM analysis of causality is well discussed for the TSS nucleation region and spreading regions, for these regions there is limited novelty. For the exciting part, the fact that both nucleation regions are independent of each other, the description remains too vague (Line 194 ff). The causality analysis for both marks at the distal is unclear when looking at the

graph and not actually described in the text.
3. The distal nucleation regions may be controlled independently and H3K4me3 appears to be influencing H3K27me3 (or the other way around). However, the mathematical model is limited to the H3K27me3 modification. The dynamics of H3K27me3 at FLC for both annual and perennial models have been presented before, it remains unclear wherein the novelty in the approach can be found.
4. Distal H3K27me3 as stabilizer of H3K4me3. The presented shift data are very interesting. However, the hypothesis that distal H3K27me3 stabilizes H3K4me3 comes out of the blue, it has not been discussed as a result of the modelling (see comments above). The hypothesis seems to ignore the conclusion from above that the temperature sensor in independent for both nucleation regions as well as assumes a causality that was not actually presented by CCM (or not clearly discussed). The conclusion "..H3K27me3 antagonized H3K4me3 at the distal region, which enables irreversible upregulation of AhgFLC in spring …" is not made clear to the reader.
5. Discussion 296: If H3K27me3 renders the locus insensitive to the cold; how can the distal region have a short term temperature memory?
Smaller points:
1. Introduction 54ff discussion on the role of COOLAIR. The function of COOLAIR in FLC repression is a bit more controversial as the authors suggest. Data from Helliwell et al. 2011 should be included in the introduction.
2. Note that not all manuscripts listed in line 66 do actually discuss or model DNA-replication. For e.g. Angel et al. 2011 excluded replication-based spreading from the mathematical model.
3. The last summary sentence 93 is far from clear. It is in part very general, it may also be wrong as it is not shown that H3K27me3 is actually causal for the lack of temperature responsiveness.
4. 287 inputs not input

Reviewer #4 (Remarks to the Author):

This manuscript describes the correlation between AhgFLC histone modifications and expression by using nonlinear time series analyses and the function of H3K27me3 in the
seasonal regulation of AhgFLC by using mathematical modelling. The causality between H3K27me3 and expression of FLC was resolved by using EDM-CCM. EDM-CCM was previously used for mathematical modeling of ecological variations (Sugihara et al, 2012).

The main conclusion was that antagonistic relationship between H3K4me3 and H3K27me3 at "distal nucleation" region enables irreversible upregulation of AhgFLC which overcomes temperature fluctuations.

Mathematical modeling itself is very robust and could be very useful to address the different effects of histone modifications on the perennial behavior of FLC orthologs in natural setting. But there are a few shortcomings authors need to address.

1. Although authors defined "distal nucleation" based on the peaks of both H3K4me3 and H3K27me3, it is expected them to be as the region is the promoter regions of coolair anti-sense transcripts. Transient enrichment of H3K4me3 at this region was very well documented and correlates with the expression of coolair (not FLC). This aspect was not addressed.
2. Previous studies showed that H3K36me3, but not H3K4me3, anti-correlates with H3K27me3. Unfortunately, authors did not examine the level of H3K36me3. Inclusion of this modification may result in different conclusion of the modeling.

Reviewer #5 (Remarks to the Author):

My expertise lies in mathematical modeling and time series analysis. Please note this when reading my review.

Kudoh and coworkers present results of a field study in which the dynamics of methylation at the floral repressor locus C in a natural Arabidopsis halleri population was investigated. Over a period of two years, samples of Arabidopsis halleri leaves were taken every two weeks and analyzed by molecular biology. In this way, the authors were able to create a unique dataset containing time series of transcription and methylation. The time series were then analyzed using modern methods of time-series analysis (convergent cross-mapping). In particular, dynamic coupling between variables ("causality") was tested. As a result, the authors present an explanation for the seasonal dynamics of gene expression at the flowering repressor locus. This explanation is formalized by a mathematical model. Further empirical evidence is given by transplantation experiments.

MAIN COMMENTS

(1) I found that an excellent study. In general, the manuscript is well-written (but see my comment below). The most novel aspect seems to me to be the use of nonlinear time series analysis to elucidate the methylation dynamics in natural populations.

(2) The authors need to describe in more detail how sampling was conducted. It is not clear from the methods whether (i) 40 plants were randomly selected at the beginning of the field study, and only leaves from these 40 individuals were taken during the entire two-year sampling period, or (ii) 40 plants were randomly selected every two weeks, so that the removed leaves came from different plants on different sampling days. This distinction is critical (see next two comments).

(3) If the sampling had taken place as described in (2)(i), there are 40 independent time series data sets describing the methylation/transcription dynamics of 40 different plant individuals. Each of the 40 data sets would deserve its own analysis, and these results should be shown.

(4) If the sampling had taken place as described in (2)(ii), there is one time series data set describing the methylation/transcription dynamics of the whole Arabidopsis halleri population (and NOT of Arabidopsis halleri plant individuals). This is because samples were taken randomly from the population. The reconstructed attractor would therefore describe the population wide methylation dynamics. This would suggest that transcription/methylation within the population is dynamically coupled. The authors would need to discuss if results are better explained by a common external driver (e.g., temperature) or some hidden coupling between plant individuals (e.g., plant volatiles).

(5) The mathematical model is reasonable and intuitive. I have only minor comments.
Please explain better what it meant with model with feedback and model without feedback.
Please explain in more detail how simulations were conducted. What software was used?
The model consists of a set of ordinary differential equations. Time is assumed to be continuous.
Please explain how the discrete temperature data were incorporated.

(6) This is a great study. Please try to make it more appealing to a general audience.
The manuscript would benefit from 1-2 general paragraphs in the introduction as well as an outlook in the discussion.
The authors could highlight the advantages of their plant system over animal systems. I can not imagine an animal system in which the methylation dynamics in a natural population can be examined similarly well.
In systems biology, the circadian oscillator has become a major system for studying gene regulation.
After reading the manuscript, I think that the seasonal dynamics of flowering loci may play a similarly prominent role in the study of methylation dynamics in natural populations.

SPECIFIC COMMENTS

Line 2-3. Please try to find a better title. The current title is not very appealing for a general audience. Please try to avoid abbreviations such as „H3K27me3".

Line 27. Better to use „short-term FLUCTUATIONS of temperature" instead of „short-term NOISES of temperature". The term „noise" has a precise mathematical definition. „Fluctuation" is more general and includes cycles, random fluctuations (noise), and deterministic chaos.

line 329. Change 13:30 to 12:30.

line 330-340. Please split the paragraph in two.

line 339-340. Please clarify the meaning of cross-linking.

Response letter

We appreciate comments on our manuscript by which we are possible to largely improve our manuscript. In the following letter, original comments are in italics and we re-numbered the comments (in bold face letters). Our replies are listed following each numbered comment in plain letters. The line numbers in our replies correspond with those in the final version of the uploaded file.

Reply to the Reviewers' comments:

1. *Reviewer #1 (Remarks to the Author):*
This is an interesting paper showing a very large and impressive dataset with extensive analysis that is well placed in the context of the recent literature. The data is consistent with current knowledge and there are also some surprising and exciting findings. In most cases, the conclusions are well supported by the data in a clear way. I do comment on one case (comment on lines 257-259, 267-269) where the connection between the results and conclusion is not clear in the text. There is also a possible error in the conceptual underpinning of the mathematical model (see comment on Fig.5a below) that needs to be addressed. I also include some more minor comments below.

Thank you for evaluating our manuscript. We revised our manuscript in response to all the comments. Please see below for further details.

2. *Comments on scientific content:*
line 110: The authors say K4 "followed" the mRNA, but actually K4 seems to drop earlier in Fig.1b,c.

We replaced the word “followed” with “was similar to”. The reason why K4 seems to drop earlier when we compare Fig.1b and c is partly because mRNA and K4 were plotted in logarithmic and linear scales, respectively. We intended to say that the two patterns were similar, but the original sentence was misleading as the referee commented. The similarity of dynamics of mRNA and K4 at the nucleation region is later stated at lines 184−187 in the revised manuscript, and tested by using the Lissajous curves in Supplementary Fig. 9a, b, g, h.

3. *line 149: what do the authors mean by the period being “colinear”?*

We replaced "was collinear with" by "positively depended on" (line 166).

4. *line 169: What is meant by the word "advanced" in this sentence? Is this related to the above point? In general, for this section on Lissajous curves, what has been learned here that could not have been learned from a detailed inspection of Fig. 1?*

We replaced "advanced H3K4me3" by "the phase advances of H3K4me3 at these amplicons" (line 189). In the original sentence, we meant the phase advances of H3K4me3 at amplicons VI−VIII relative to mRNA. The usage of Lissajous curves show the phase differences between the variables more clearly than just comparing seasonal dynamics in Fig. 1.

5. *lines 208-212: Is the UM state necessary for the model? Is it reasonable? Also based on line 117 earlier, the drop in K27 is synchronised, which seems not to support the existence of this state.*

As the reviewer pointed out, we observed a simultaneous decrease in the H3K27me3 levels between different regions except for the start timing of decrease. H3K27me3 at nucleation region started to decrease 1−2 time points earlier than the other regions as we see it in the Lissajous curves especially in the measurement conducted during the second year (Supplementary Fig. 7 and 8j−r). We added a sentence to explain this result (lines 244−247). Based on these results, we incorporated The UM state in the mathematical model. Although UM state appeared to be transient in our modelling, exclusion of UM state failed to reproduce the difference in initiation timing of H3K27me3 decrease between different regions.

6. *Fig.5a: The transition MM->UM depends on transcription but according to the way it is defined in the model, transcription in trans is assumed to affect this transition. This means that transcription from other loci (and also in other cells) is somehow affecting the removal of K27 at the MM loci. Previous work has shown links to transcription but this is "in cis", meaning at the same locus.*

In the presented model, H3K27me3 and transcription were modelled at the tissue level, not the cellular or locus level because we have the data on leaf samples. Therefore, each state (UU, MU, MM or UM) can take continuous value between 0 and 1. We consider that the differential equations are suitable for this purpose. We added "at the tissue level" for clarity at lines 236−237.

7. *lines 228-238: I don't really understand this paragraph. More intuition would be helpful. Furthermore, the model with/without feedback does not seem to be fully defined anywhere.*

Because we updated the models, we rewrote the paragraph, and explained the models without the feedback in detail (lines 271−282).

8. *lines 247-248: What is this speculation based on? If it is based on Fig.4c, that is not clear in the text and it should be stated and explained.*

We explained what the speculation is based on (lines 290−294).

9. *line 254: Did K4 really decrease? It seems to be noisy (going first up then down), without a clear trend.*

As the reviewer points out, the trend is ambiguous. Thus, we edited the sentence for accuracy (line 299).

10. *line 255-257: K4 in distal nucleation region is reported to respond to cold earlier than at the nucleation region, but in Fig.1 it seems to respond later.*

The comment is reasonable, and it is difficult to judge whether the H3K4me3 level at the distal nucleation region responded to cold earlier than that at the nucleation region in terms of the initiation timings of the response. Therefore, we removed the corresponding sentence for accuracy. The entire dynamics of the H3K4me3 level at the distal nucleation region was advanced in phase relative to that of mRNA level (the phase difference analyses in Supplementary Fig. 9c−e, i−k), and the pattern was described earlier in the manuscript (line 187−190).

11. *lines 257-259, 267-269: What is the evidence of the H3K27 role here? There is no reference to the CCM results; is that the evidence? I would instead suggest postponing speculation about H3K27 (and COOLAIR, as you already have done) to the discussion section.*

Following the suggestion, we removed the first sentence and modified the second sentence and speculation about H3K27me3 (lines 290−294 and 309−311).

12. *SMA analysis: This analysis is using only mean temperatures. But in A. thaliana it was shown that daily maxima and minima are key in temperature sensing for vernalization. Could you comment on this choice?*

Because temperature sensing during *FLC* upregulation in perennial is unknown, we used the daily mean temperature for the SMA analyses for the purpose to examine the response period of expression and histone modifications. Following the comment, we performed the SMA analyses for the daily maximum and minimum temperatures and obtained similar results to those for the daily mean temperature, and we added the results in the revised version (Supplementary Fig. 5b−g, lines 166−168).

13. *EDM-CCM analysis: This is described clearly and seems reasonable but, for full disclosure, I am not an expert on this type of modelling.*

Please see the responses to the comments **31**, **43−46**.

14. *line 328-329: How was it completed in half an hour if it started at 12 and finished at 13:30? Not 1.5h?*

This is our mistake in English. We replaced "within half an hour" with "within one and a half hours" (lines 374−375).

15. *Fig.4c: If this panel is important, you could make it a bit bigger and clearer.*

We enlarged the panel following the comment (Fig. 4e).

16. *lines 230-233: Sentence unclear.*

We modified the sentence for clarity (lines 276−278).

17. *Comments on language and typographical errors:*
line 30: "Furthermore, we show that the long-term…"
line 80: "revealed" -> "measured"?
line 95: "enables"
lines 148: "best explained" (no "the")
line 172: "performed a causality"

line 229: "affects"
line 235: "best explained" (no "the")
line 238: "to the long-term"
line 242: "similarity"
line 303: "fluctuations"

Thank you for the corrections. We modified them accordingly.

18. *Reviewer #2 (Remarks to the Author):*
FLC is a potent regulator of flowering time in both annual and perennial species within the Brassicacae. In this manuscript the authors have carried out a comprehensive analysis of FLC expression and chromatin environment, in the perennial Arabidopsis halleri, over a 2 year period under natural conditions. Modelling demonstrated that there is a bi-directional relationship between transcription and H3K37me3 modification of AhgFLC. In addition, marking of AhgFLC chromatin with H3K27me3 allows reversible FLC expression over the life cycle of this perennial plant but ensures that transcription of AhgFLC is insensitive to cold temperatures during the up-regulation phase in spring.
This is a nice piece of work using plants that are grown under natural conditions over two years, as well as experiments that perturb the normal seasonal changes in temperature allowing the authors to gain some novel insights into the regulation of the much studied FLC locus over the natural life cycle. These experiments were done in a perennial species, and so contribute to our understanding of how the perennial life cycle is regulated. I have a few criticisms of the manuscript which I have outlined below:

Thank you for evaluating our manuscript. We revised our manuscript in response to all the comments. Please see below for further details.

19. *1. I am concerned by the use of Arabidopsis as an abbreviation in both the title and throughout the text as this I misleading. While A. halleri is a member of the Arabidopsis genus its life cycle is very different from that of A. thaliana with which most readers will be more familiar. For clarity I strongly recommend that the authors stick with the nomenclature A. halleri throughout.*

As the reviewer recommend, we changed the "*Arabidopsis FLC*" to "*AhgFLC*" at three locations: lines 31, 317, and 361 in the revised manuscript. For the title, we revised it following a comment made by reviewer #5 (see the comment **49**), and the new title does not contain

“*Arabidopsis*”.

20. *2. P7-8, “Function of AhgFLC H2K27me3 in the seasonal gene regulation”; in this section the author use a mathematical model to predict H3K27me3 and mRNA dynamics under seasonal variation in temperature. They assume that transcription enhances the removal of H3K27me3 and that there are unidirectional circular transitions between the four modification states (UU to MU to MM to UM to UU). The predicted H3K27me3 levels agree well with observed data leading the authors to conclude that their “simple model explains the major dynamic over weeks without previously assumed interaction with active modifications”. I may be missing something but as transcriptional activity is closely linked with the level of H3K4me3 at the nucleation region (see Fig 1 b,c) I do not think that the authors can exclude the possibility that it is the level of H3K4me3, rather than transcription per se, that influences H3K27me3 levels. The Lissajous plots for HK4me3 (amplicons 1 & 2) against mRNA support my comments.*

Following this comment and the comment **29**, we updated the models to include the H3K4me3 dynamics at the two nucleation regions (lines 234–282, Fig. 5). The results suggested that the feedback from H3K4me3 to H3K27me3 at the nucleation region is required for *AhgFLC* mRNA to respond to the seasonal trend of temperature. Thus, the results were similar to those when we assumed that transcription antagonises H3K27me3 as in the original model. We chose the model with H3K4me3 following this and next comments.

21. *3. I strongly recommend that the authors provide Lissajous curves for the correlation between H3K4me3 (at the nucleation region) and H3K27me3.*

We revised Fig. 3 and added a new Supplementary Fig. 7 to show the Lissajous curves for the correlation between H3K4me3 (at amplicon I) and H3K27me3 and added a sentence explaining the result (lines 178–183).

22. *4. I suggest that the authors define more explicitly the abbreviations UU, MU, MM and UM so that it is clear what they refer to. Initially I wondered if the two letters referred to the status of H3 at the two FLC alleles, or to the two different molecules of H3 in a single nucleosome but finally decided that the first letter probably refers to the status of H3 modification in the nucleation region and the second letter to the linker region.*

We changed the symbols UU, MU, MM and UM to U_NU_D, M_NU_D, M_NM_D and U_NM_D, to better represent that the first letter refers to the nucleation region and the second letter refers to the

distal nucleation region (Fig. 5a). We also added the explanation in the text (lines 239−242).

23. *5. The significance of the second conclusion – that H3K27me3 at the distal nucleation region prevents downregulation of AhgFLC during cold weather in spring is relevant to perennial rather than annual species of Arabidopsis. This needs to be clearly stated as it is the seasonal upregulation of FLC after winter that is essential for a perennial life cycle.*

We added a sentence to clearly state the point (lines 335−337).

24. *6. P8, line 236; Here the authors refer to Fig 5e, but there is no such figure in the version I received.*

In the previous version, Fig. 5e was placed next to Fig. 5a on the top of the figure 5. We removed Fig. 5e to avoid redundancy.

25. *7. One wonders why the authors did not check COOLAIR transcription (P10 lines 293-299) given that they had RNA available to do this.*

We confirmed the presence of *AhgCOOLAIR* transcripts by cloning the sequences (Supplementary Fig. 1). The remaining time-series RNA samples are not enough for the analyses of *AhgCOOLAIR*. Thus, we are planning the experiments to analyse the dynamics of *AhgCOOLAIR*, which will be included in a future paper.

26. *8. I don't really understand what the authors are proposing in the last sentence – please explain or delete.*

We revised the last sentence to make the suggestion clear (lines 365−368).

27. *Minor comments:*
P9 line 281; I suggest replacing the word 'innate" with "endogenous"

We replaced the word accordingly (line 324).

28. *Reviewer #3 (Remarks to the Author):*
The manuscript by Nishio and colleagues describes an exceptional data set: the authors have conducted a long term (2-years) field study on a natural population of the perennial plant

species Arabidopsis halleri and collected in a fine-grained time course samples for chromatin immunoprecipitation and expression analysis. The authors focussed on the kinetics of A. halleri FLC, measuring expression and the dynamics of H3K27me3 and H3K4me3 across the locus. The authors then analysed their data by mathematical approaches that include linear regression comparison between measured traits and windows of past temperature, empirical dynamic modelling using established causality test and ODE-based modelling of chromatin states. Despite the real interesting dataset, the modelling approach is a little disappointing in terms of novelty and doesn't seem to give full justice to the great data set. Maybe the authors can go back to the model, or explain the points listed below to my satisfaction.

Thank you for evaluating our manuscript. We revised our manuscript in response to all the comments. Please see below for further details.

29. *FLC is a well-studied locus in the annual model A. thaliana, but has also been studied in perennial Brassica species. The modelling approaches confirmed existing models and predictions on the A. thaliana FLC locus concerning down-regulation of the gene in response to cold temperatures (vernalization). Novel aspects that are typical to perennial FLC regulation would be a better understanding of the processes involved in FLC upregulation after flowering. The authors describe a distal nucleation region, located downstream of FLC that may correspond to the promoter/TSS of the anti-sense transcript COOLAIR. Unfortunately, the ODE approach excludes the H3K4me3 modification, which fluctuates at the distal nucleation region; therefore the observation is not actually addressed in the mathematical model.*

Following this comment and the comment **20**, we revised the model to include the H3K4me3 dynamics at the two nucleation regions (lines 234−282, Fig. 5). The results suggested that the feedback from H3K4me3 to H3K27me3 at the nucleation region is required for *AhgFLC* mRNA to respond to the seasonal trend of temperature. The novelties of our approaches are: 1. modelling the responses of histone modifications to the natural fluctuating temperature, 2. modelling histone modifications in both down- and up- regulation of a perennial *FLC* locus, 3. modelling histone modifications at the distal nucleation region.

30. *Major points:*

1. Paragraph on co-linearity between regions and temperature medians across windows. Everything is quite coherent until the conclusion. Different temperature sensors are implied for both nucleation regions. The proximal region appears to react to a short-term trend while TSS nucleation and spreading integrates long-term trends. That is rather interesting and could mean

that if cold periods are interrupted often enough, the COOLAIR promoter cannot fire anymore to prevent FLC upregulation, possibly because a specific transcription factor is not present. But why go from there to the statement "—we conclude that the H3K27me3 levels are indexing the seasonal temperature throughout the year…" where is the novelty in this statement?

The comment is reasonable and we revised the paragraph (lines 149−170). We removed the last sentence and added a sentence to explain that the two nucleation regions represent distinct functional units (lines 168−170).

31. *2. Analysis of Lissajou's curves for H3K4me3 at the distal nucleation region suggests that H3K4me3 dynamics advance FLC expression levels. CCM analysis of causality is well discussed for the TSS nucleation region and spreading regions, for these regions there is limited novelty. For the exciting part, the fact that both nucleation regions are independent of each other, the description remains too vague (Line 194 ff). The causality analysis for both marks at the distal is unclear when looking at the graph and not actually described in the text.*

Following the comments, we added the results of CCM for H3K4me3 between the proximal and distal nucleation regions, and added a paragraph to fully explain the findings for the distal nucleation region (lines 208−218).

32. *3. The distal nucleation regions may be controlled independently and H3K4me3 appears to be influencing H3K27me3 (or the other way around). However, the mathematical model is limited to the H3K27me3 modification. The dynamics of H3K27me3 at FLC for both annual and perennial models have been presented before, it remains unclear wherein the novelty in the approach can be found.*

We revised the model as we mentioned in the response to the comment **29**.

33. *4. Distal H3K27me3 as stabilizer of H3K4me3. The presented shift data are very interesting. However, the hypothesis that distal H3K27me3 stabilizes H3K4me3 comes out of the blue, it has not been discussed as a result of the modelling (see comments above). The hypothesis seems to ignore the conclusion from above that the temperature sensor in independent for both nucleation regions as well as assumes a causality that was not actually presented by CCM (or not clearly discussed). The conclusion "..H3K27me3 antagonized H3K4me3 at the distal region, which enables irreversible upregulation of AhgFLC in spring …" is not made clear to the reader.*

Following the comment, we added the explanations for the hypothesis to make the reasoning clear (lines 290−294). Besides, we revised the concluding sentence in this paragraph to make it more straightforward (lines 309−311).

34. *5. Discussion 296: If H3K27me3 renders the locus insensitive to the cold; how can the distal region have a short term temperature memory?*

H3K27me3 represses *AhgCOOLAIR* and renders sense transcription insensitive to cold in the upregulation phase. In contrast, when H3K27me3 is absent from the region in winter, H3K4me3 at the distal nucleation region accumulated within a short period. We edited the sentences to make these points clear (lines 342−346).

35. *Smaller points:*
1. Introduction 54ff discussion on the role of COOLAIR. The function of COOLAIR in FLC repression is a bit more controversial as the authors suggest. Data from Helliwell et al. 2011 should be included in the introduction.

Following the comment, we added the reference at the location (line 59).

36. *2. Note that not all manuscripts listed in line 66 do actually discuss or model DNA-replication. For e.g. Angel et al. 2011 excluded replication-based spreading from the mathematical model.*

The comment is reasonable and we moved and cited some of the references at the appropriate place.

37. *3. The last summary sentence 93 is far from clear. It is in part very general, it may also be wrong as it is not shown that H3K27me3 is actually causal for the lack of temperature responsiveness.*

Following the comment, we revised the sentence (lines 96−99).

38. *4. 287 inputs not input*

We modified it accordingly.

39. *Reviewer #4 (Remarks to the Author):*

This manuscript describes the correlation between AhgFLC histone modifications and expression by using nonlinear time series analyses and the function of H3K27me3 in the seasonal regulation of AhgFLC by using mathematical modelling. The causality between H3K27me3 and expression of FLC was resolved by using EDM-CCM. EDM-CCM was previously used for mathematical modeling of ecological variations (Sugihara et al, 2012).

The main conclusion was that antagonistic relationship between H3K4me3 and H3K27me3 at "distal nucleation" region enables irreversible upregulation of AhgFLC which overcomes temperature fluctuations.

Mathematical modeling itself is very robust and could be very useful to address the different effects of histone modifications on the perennial behavior of FLC orthologs in natural setting. But there are a few shortcomings authors need to address.

Thank you for evaluating our manuscript. We revised our manuscript in response to all the comments. Please see below for further details.

40. *1. Although authors defined "distal nucleation" based on the peaks of both H3K4me3 and H3K27me3, it is expected them to be as the region is the promoter regions of coolair anti-sense transcripts. Transient enrichment of H3K4me3 at this region was very well documented and correlates with the expression of coolair (not FLC). This aspect was not addressed.*

Following the comment, we revised the paragraph to define the three regions based on previous knowledges at lines 104−110 in the revised manuscript.

41. *2. Previous studies showed that H3K36me3, but not H3K4me3, anti-correlates with H3K27me3. Unfortunately, authors did not examine the level of H3K36me3. Inclusion of this modification may result in different conclusion of the modeling.*

As the reviewer recognise, one of the novelties is modelling H3K4me3 at the two nucleation regions. According to Yang et al., 2014, H3K4me3 accumulation level is high at both two nucleation regions, but H3K36me3 accumulation level is low at the distal nucleation region. In updated mathematical model, the feedback regulation between H3K27me3 and H3K4me3 at the two nucleation regions could reproduce the seasonal trend of *AhgFLC* expression. As the reviewer suggests, inclusion of H3K36me3 will lead to additional findings. We discussed this point in the revised manuscript (lines 330−333).

42. *Reviewer #5 (Remarks to the Author):*

My expertise lies in mathematical modeling and time series analysis. Please note this when reading my review.

Kudoh and coworkers present results of a field study in which the dynamics of methylation at the floral repressor locus C in a natural Arabidopsis halleri population was investigated. Over a period of two years, samples of Arabidopsis halleri leaves were taken every two weeks and analyzed by molecular biology. In this way, the authors were able to create a unique dataset containing time series of transcription and methylation. The time series were then analyzed using modern methods of time-series analysis (convergent cross-mapping). In particular, dynamic coupling between variables ("causality") was tested. As a result, the authors present an explanation for the seasonal dynamics of gene expression at the flowering repressor locus. This explanation is formalized by a mathematical model. Further empirical evidence is given by transplantation experiments.

Thank you for evaluating our manuscript. We revised our manuscript in response to all the comments. Please see below for further details.

43. *MAIN COMMENTS*

(1) I found that an excellent study. In general, the manuscript is well-written (but see my comment below). The most novel aspect seems to me to be the use of nonlinear time series analysis to elucidate the methylation dynamics in natural populations.

Following the comment, we revised sentences in the introduction section to highlight this novelty at lines 89−92 in the revised manuscript.

44. *(2) The authors need to describe in more detail how sampling was conducted. It is not clear from the methods whether (i) 40 plants were randomly selected at the beginning of the field study, and only leaves from these 40 individuals were taken during the entire two-year sampling period, or (ii) 40 plants were randomly selected every two weeks, so that the removed leaves came from different plants on different sampling days. This distinction is critical (see next two comments).*

Our case is similar to (ii), and 40 plants were newly selected every two weeks. We revised the explanation of the field sampling in the Methods section for accuracy (lines 376−383).

45. *(3) If the sampling had taken place as described in (2)(i), there are 40 independent time series data sets describing the methylation/transcription dynamics of 40 different plant individuals. Each of the 40 data sets would deserve its own analysis, and these results should be shown.*

This is not our case. Please see below. Because a large amount of leaf tissue was necessary for ChIP experiments, ten leaves were pooled (1 g per replicate) and we prepared four biological replicates.

46. *(4) If the sampling had taken place as described in (2)(ii), there is one time series data set describing the methylation/transcription dynamics of the whole Arabidopsis halleri population (and NOT of Arabidopsis halleri plant individuals). This is because samples were taken randomly from the population. The reconstructed attractor would therefore describe the population wide methylation dynamics. This would suggest that transcription/methylation within the population is dynamically coupled. The authors would need to discuss if results are better explained by a common external driver (e.g., temperature) or some hidden coupling between plant individuals (e.g., plant volatiles).*

Following the comments, we analysed causality from temperature to each of mRNA, H3K4me3, and H3K27me3 using CCM. We added the results (Supplementary Fig. 13) and explained them in the text (lines 223−230). We explained that this common driver caused coupled response between multiple plant individuals within the population (lines 230−232).

47. *(5) The mathematical model is reasonable and intuitive. I have only minor comments.*
Please explain better what it meant with model with feedback and model without feedback.
Please explain in more detail how simulations were conducted. What software was used?
The model consists of a set of ordinary differential equations. Time is assumed to be continuous.
Please explain how the discrete temperature data were incorporated.

Following the comments **20** and **29**, we revised the model to include the H3K4me3 dynamics at the two nucleation regions (lines 234−282, Fig. 5). We included the detailed explanation of the models (lines 271−282). We also added a more detailed explanation about the simulation including the software in Methods section (lines 555−567). The temperature data was interpolated by the approxfun function of R before the simulation. We added the explanation in Methods section (lines 566−567).

48. *(6) This is a great study. Please try to make it more appealing to a general audience. The manuscript would benefit from 1-2 general paragraphs in the introduction as well as an outlook in the discussion.*
The authors could highlight the advantages of their plant system over animal systems. I can not imagine an animal system in which the methylation dynamics in a natural population can be examined similarly well.
In systems biology, the circadian oscillator has become a major system for studying gene regulation. After reading the manuscript, I think that the seasonal dynamics of flowering loci may play a similarly prominent role in the study of methylation dynamics in natural populations.

Following the comment, we added sentences to highlight the advantages of our plant system over animal systems in the introduction section (lines 35−39) and modified the last sentence in the discussion section to include an outlook (lines 365−368).

49. *SPECIFIC COMMENTS*
Line 2-3. Please try to find a better title. The current title is not very appealing for a general audience. Please try to avoid abbreviations such as „H3K27me3“.

We revised the title to make it more appealing to general readers.

50. *Line 27. Better to use „short-term FLUCTUATIONS of temperature“ instead of „short-term NOISES of temperature“. The term „noise“ has a precise mathematical definition. „Fluctuation“ is more general and includes cycles, random fluctuations (noise), and deterministic chaos.*

Following the comment, we replaced the word “noise” with “fluctuation” (lines 48 and 319).

51. *line 329. Change 13:30 to 12:30.*

This is our mistake in English. The sampling was conducted from 12.00 to 13.30. We replaced “within half an hour” with “within one and a half hours” (line 375).

52. *line 330-340. Please split the paragraph in two.*

We split the paragraph in two (lines 376−391)

53. *line 339-340. Please clarify the meaning of cross-linking.*

We replaced “cross-linking was performed” with “samples were fixed with formaldehyde” (lines 389–391).

The end of the letter.

Reviewers' comments:

Reviewer #1 (Remarks to the Author):

The authors have responded to most of my comments and the manuscript has improved. However, one item has not been satisfactorily resolved: the transition MM->UM in the model depends on transcription from other loci, including in other cells, and this then affects removal of K27 at MM loci. There is no evidence to my knowledge that this is true; only transcription at the individual locus in question will affect removal of K27me3, not from other loci. I therefore remain concerned that the equations contain a flaw. Just because the experiments and model are constructed at a tissue level does not address this particular point.

Reviewer #2 (Remarks to the Author):

The authors have addressed my criticisms well during the revision of this manuscript. I have only two very minor suggestions for changes in wording.
1. line 37; I suggest "plants are sessile" rather than "plants live sessile"
2. line 43-44; I suggest "temperatires greatly fluctuate in the natural enviromnent of A halleri" rather than "in the natural population of A. halleri"

Reviewer #3 (Remarks to the Author):

It was a real pleasure to read the revised version of the manuscript. The authors have answered all points raised in my first revision to my satisfaction. In particular, the extension of the modelling approach to the distal nucleation region added novel aspects and provides a mechanism for the "ratchet-like" regulation of FLC in perennial Brassicaceae.

Reviewer #4 (Remarks to the Author):

No further comments

Reviewer #5 (Remarks to the Author):

Thanks for revising the manuscript. I do not have any further comment. I recommend publication.

Response letter

We appreciate comments on our manuscript by which we are possible to largely improve our manuscript. In the following letter, original comments are in italics and we re-numbered the comments (in bold face letters). Our replies are listed following each numbered comment in plain letters. The line numbers in our replies correspond with those in the final version of the uploaded file.

Reply to the Reviewers' comments:

1. *Reviewer #1 (Remarks to the Author):*

The authors have responded to most of my comments and the manuscript has improved. However, one item has not been satisfactorily resolved: the transition MM->UM in the model depends on transcription from other loci, including in other cells, and this then affects removal of K27 at MM loci. There is no evidence to my knowledge that this is true; only transcription at the individual locus in question will affect removal of K27me3, not from other loci. I therefore remain concerned that the equations contain a flaw. Just because the experiments and model are constructed at a tissue level does not address this particular point.

Thank you for evaluating our revised manuscript. As the reviewer pointed out, the assumption of cis regulation of *FLC* was not clearly stated in our previous manuscript. We added Supplementary Note (page 23–27 of the Supplementary Information file) to explain how we derived differential equation models from stochastic models that assumes the independent regulation of *AhgFLC* at each locus, to link the tissue-level observations in experiments with the locus-level H3K27me3 and H3K4me3 states. The probabilities that an *AhgFLC* locus in a single cell is in the U_NU_D, M_NU_D, M_NM_D, and U_NM_D states can be considered as the proportion of cells in these states in a tissue composed of a large number of cells. Furthermore, we revised the result and method sections to explain these points (lines 246–251 and 551–555).

2. *Reviewer #2 (Remarks to the Author):*

The authors have addressed my criticisms well during the revision of this manuscript. I have only two very minor suggestions for changes in wording.

1. line 37; I suggest "plants are sessile" rather than "plants live sessile"

2. line 43-44; I suggest "temperatires greatly fluctuate in the natural enviromnent of A halleri" rather than "in the natural population of A. halleri"

Thank you for evaluating our revised manuscript. We modified them accordingly.

3. *Reviewer #3 (Remarks to the Author):*

It was a real pleasure to read the revised version of the manuscript. The authors have answered all points raised in my first revision to my satisfaction. In particular, the extension of the modelling approach to the distal nucleation region added novel aspects and provides a mechanism for the "ratchet-like" regulation of FLC in perennial Brassicaceae.

Thank you for evaluating our revised manuscript.

4. *Reviewer #4 (Remarks to the Author):*

No further comments

Thank you for evaluating our revised manuscript.

5. *Reviewer #5 (Remarks to the Author):*

Thanks for revising the manuscript. I do not have any further comment. I recommend publication.

Thank you for evaluating our revised manuscript.

The end of the letter.

Reviewers' comments:

Reviewer #1 (Remarks to the Author):

The authors have now provided a much expanded discussion of the derivation of the differential equations. This is a big improvement and hopefully readers will now be in a better position to understand the assumptions built into the model. There is, however, still one difficulty I have. This still stems from the term representing the transition MM->UM. In the original stochastic model this arises from multiplying the probabilities together for being in the MM state and being in the An state. However, such a product is only correct if the two probabilities are uncorrelated with each other, in other words the probability of being in the MM state is independent of being in the An state. I don't think this is true in the model the authors propose. At the very least the procedure the authors apply is an assumption (technically a mean field assumption) that may not be entirely correct. This assumption must at the very least be stated.

Response letter

We appreciate a comment on our manuscript by which we are possible to largely improve our manuscript. In the following letter, the original comment is in italics. Our reply follows the comment in plain letters. The line numbers for the main text in our reply correspond with those in the final version of the uploaded file. Following the comment, we added a new analysis (Supplementary Fig. 15). We attached an additional file for Supplementary Information (SI_Nat.Commun_200309_highlighted.pdf), in which edited sentences are highlighted in yellow.

Reply to the Reviewers' comments:

1. *Reviewer #1 (Remarks to the Author):*

The authors have now provided a much expanded discussion of the derivation of the differential equations. This is a big improvement and hopefully readers will now be in a better position to understand the assumptions built into the model. There is, however, still one difficulty I have. This still stems from the term representing the transition MM->UM. In the original stochastic model this arises from multiplying the probabilities together for being in the MM state and being in the An state. However, such a product is only correct if the two probabilities are uncorrelated with each other, in other words the probability of being in the MM state is independent of being in the An state. I don't think this is true in the model the authors propose. At the very least the procedure the authors apply is an assumption (technically a mean field assumption) that may not be entirely correct. This assumption must at the very least be stated.

Thank you for evaluating our revised manuscript. As the reviewer pointed out, the assumption that the probability of being in the M_NM_D state is independent of that of being in the A_N state was not stated in our previous manuscript. We added sentences to main text (lines 245−250 and 536−537) and to Supplementary Note (page 24−29 of the Supplementary Information file) to describe that we derived differential equation models with the assumption for ease of derivation of differential equation models. To evaluate the influence of the model assumptions on our conclusion, we performed the simulation of the original stochastic models (equations S1−S8 in Supplementary Note) using the parameters optimised for the differential equation models (Supplementary Fig. 15). We confirmed that the influence of the assumptions on our conclusion of the modelling was minimal (explained in detail in page 28−29 of the Supplementary Information file). We attached an additional file for Supplementary Information

(SI_Nat.Commun_200309_highlighted.pdf), in which edited sentences are highlighted in yellow.

The end of the letter.

REVIEWERS' COMMENTS:

Reviewer #1 (Remarks to the Author):

The authors have now clarified their assumptions and I am now happy to see the manuscript published.

Response to the reviewers' comment

Reviewer #1 (Remarks to the Author):

The authors have now clarified their assumptions and I am now happy to see the manuscript published.

Thank you for evaluating our manuscript, and we really appreciate your time and effort.
